# Design, Synthesis, and Biological Evaluation of Methoxy-Substituted Chalcones and Gypsogenin-Based Hybrids as Anticancer and Antimicrobial Agents

**DOI:** 10.1021/acsomega.5c13679

**Published:** 2026-04-21

**Authors:** Nafia Gökçe Ulusoy, Safiye Emirdağ, Nuran Kahriman, Fatma Demir, Vildan Serdaroğlu, Nurettin Yaylı, Burçin Türkmenoğlu

**Affiliations:** † Ege University, Faculty of Science, Chemistry Department, Bornova, Izmir 35100, Turkiye; ‡ 52976 Karadeniz Technical University, Faculty of Science, Department of Chemistry, Trabzon 61080, Türkiye; § Erzincan Binali Yıldırım University, Department of Pharmaceutical Basic Sciences, Faculty of Pharmacy, Erzincan 24002, Türkiye

## Abstract

Chalcones and natural product-based hybrids represent important scaffolds in the development of biologically active small molecules. In this study, a series of methoxy-substituted chalcone derivatives (**1a**–**1i**) and acetyl gypsogenin-based hybrid compounds (**4a**–**4i**) were synthesized and fully characterized by spectroscopic methods. The cytotoxic activities of all compounds were evaluated against five human cancer cell lines (PANC-1, MDA-MB-231, HeLa, A549, and SH-SY5Y) and the normal HEK293 cell line using the MTT assay. Several chalcone derivatives exhibited pronounced antiproliferative activity, whereas the corresponding gypsogenin-based hybrids generally showed reduced cytotoxic effects. Tumor selectivity was further assessed by calculating the selectivity index values. Antimicrobial activity was determined using minimum inhibitory concentration assays against selected bacterial and fungal strains, revealing moderate inhibitory effects for some compounds. In addition, molecular docking studies of the most active chalcones (**1g** and **1h**) were performed to explore potential interactions with key cancer-related targets. Overall, the results highlight the chalcone scaffold as the main contributor to biological activity in this series and demonstrate that hybridization with gypsogenin did not enhance the cytotoxic potency.

## Graphical abstract

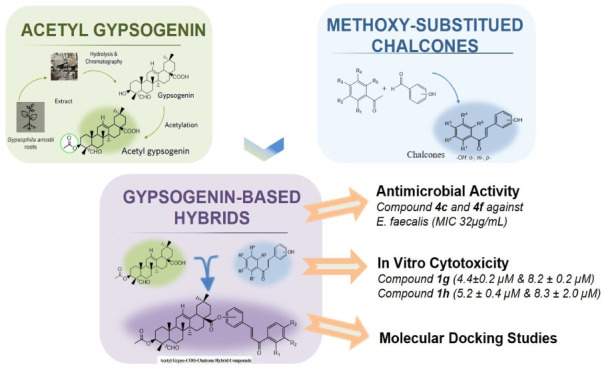

## 1 Introduction

Natural products are the richest source of compounds with a wide range of biological and pharmacological activities, characterized by diverse structural arrangements of functional groups.^1^ Research on natural products and semisynthetic approaches involves the chemical modification of naturally derived compounds to create new drug candidates with optimized properties. Natural products are excellent starting materials for synthesis and are considered valuable precursors for modern drug discovery.^2,3^

Modern research has clearly demonstrated that natural products and their semisynthetic derivatives constitute an important reservoir for the discovery of biologically active molecules with therapeutic potential.^2–4^

Chalcones, the most fundamental and widespread members of the flavonoid compounds isolated from plants, are common natural pigments that exhibit a wide range of biological activities.^5,6^ Chalcone derivatives have been reported to exhibit a broad spectrum of biological activities, including antidiabetic, anti-inflammatory, antioxidant, antimicrobial, and anticancer effects.^5,7,8^ Recent studies have further demonstrated the anticancer and antimicrobial potential of structurally diverse chalcone derivatives, highlighting their suitability as versatile bioactive scaffolds.^5,7,8^ Some chalcone derivatives, including structures closely related to compound **1h**, have previously been reported to exhibit antitumor activity. However, most earlier studies focused on single cell lines or limited biological end points, whereas the present work provides a systematic and comparative evaluation across multiple cancer cell lines under identical experimental conditions, supported by selectivity analysis and molecular docking studies.^17^

Among these, methoxy-substituted chalcones have attracted particular attention, as methoxy groups are known to modulate lipophilicity, membrane permeability, and target-binding affinity, thereby influencing biological activity.^9^

The wide-ranging biological activities and synthetic accessibility of chalcones have encouraged the development of hybrid molecules, in which the chalcone scaffold is combined with other bioactive natural product cores.^5^ In this context, triterpenoid saponins and their aglycones have emerged as attractive partners for molecular hybridization strategies.^10^ Saponins and their aglycone forms have been extensively reported to exhibit antiproliferative, cytotoxic, antimicrobial, and signaling pathway-modulating activities, particularly in cancer-related studies.^11,12^

Gypsogenin, a pentacyclic triterpenoid aglycone, is a major constituent of saponins isolated from plants of the *Gypsophila* genus.^10,13^ Gypsogenin and its derivatives have been shown to possess noteworthy cytotoxic and antimicrobial activities, and structural modification of this scaffold has been demonstrated to significantly influence biological performance.^14–16,18,19^ The plant *Gypsophila arrostii* was selected as the natural source of gypsogenin in this study because it is a well-documented member of the Caryophyllaceae family, known to be particularly rich in triterpenoid saponins and gypsogenin-type aglycones, making it a suitable and reliable source for semisynthetic investigations.^13^

Given the increasing global burden of cancer and antimicrobial resistance, the identification of small molecules exhibiting both anticancer and antimicrobial activities remains an important research objective. Chalcones and triterpenoid aglycones have independently demonstrated activity in both therapeutic areas, suggesting that their combination within a single molecular framework could provide valuable insights into structure–activity relationships and biological performance.^5,15,16^

In this study, chalcone derivatives (**1a**–**1i**) (Scheme 1) were first synthesized, followed by the preparation of acetylated gypsogenin (**3**) from gypsogenin aglycone (**2**) (Scheme 2). Subsequently, these two compounds were combined to afford a series of novel gypsogenin–COO–chalcone hybrid compounds (**4a**–**4i**). The synthesized compounds were evaluated for their in vitro antimicrobial and cytotoxic activities against selected microbial strains and human cancer cell lines (Tables 1–3). To further support the experimental findings and gain insight into possible molecular mechanisms, in silico molecular docking studies were performed on selected active compounds against key cancer-related targets.

**Scheme 1:**
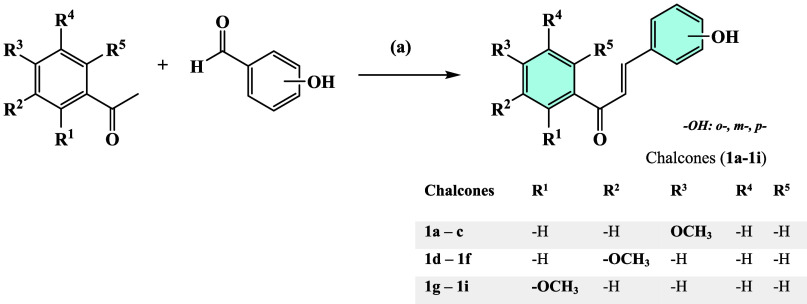
Reagents and Conditions: (a) NaOH, H_2_O/EtOH, RT

**Scheme 2:**
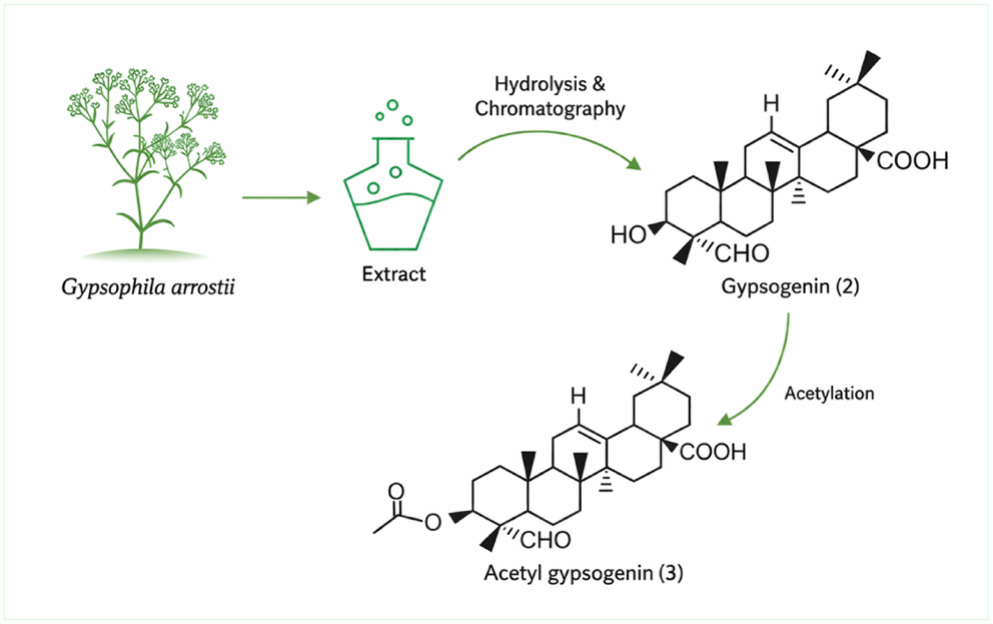
Isolation of Gypsogenin (2) and Its Acetylation to Afford Acetyl Gypsogenin (**3**)

**Table 1:** Minimum Inhibitory Concentration (MIC) Values (μg/mL) of Semi-Synthetic Compounds (**4a**–**4i**) against Microorganisms^c^

	**Gram-Positive**		Gram-Negative			Yeast
Compound No.	*B. subtilis*	*E. faecalis*	*S. aureus*	*E. coli*	*P. aeruginosa*	*C. albicans*
4a	512	256	128	>1024	512	>1024
4b	256	256	512	>1024	512	>1024
4c	64	32	128	512	>1024	>1024
4d	256	64	128	>1024	512	>1024
4e	256	512	128	1024	512	>1024
4f	128	32	256	1024	1024	>1024
4g	256	64	>1024	1024	1024	>1024
4h	512	64	>1024	>1024	>1024	>1024
4i	256	256	512	1024	512	>1024
Gypsogenin	256	64	64	>1024	1024	>1024
Acetyl-Gypso	128	64	128	>1024	1024	>1024
Ciprofloxacin	0.06	0.25 (0.25–1)^a^	0.25 (0.12–0.5)^a^	0.015	0.25 (0.25–1)^a^	-
Flukonazol	-	-	-	-	-	1 (0.25–1)^b^
DMSO	ED	ED	ED	ED	ED	ED

a Acceptable quality control of ciprofloxacin for reference bacteria MIC limit values.

b Reference for Candida acceptable quality control of fluconazole MIC limit values.

c Note: DMSO, used as the solvent for the tested compounds (denoted as ED), was included as a control and showed no inhibitory activity.

## 2 Results and Discussion

### 2.1 Chemistry

The study was carried out in three main stages. In the first stage, the root extract of *Gypsophila arrostii*, obtained commercially, was sequentially hydrolyzed under basic and acidic conditions in ethanol using KOH and HCl. The hydrolyzed mixture was processed to isolate the pure gypsogenin compound from the CH_2_Cl_2_ phase. Subsequently, acetylation of the −OH group located at the C-3 position of the purified gypsogenin compound (Scheme 2) was performed at room temperature using acetic anhydride and DMAP in THF, followed by purification. The purification procedure was described in detail in our previous study.^18^

In the second stage, chalcone derivatives were synthesized through base-catalyzed Claisen–Schmidt condensation using commercially available monosubstituted acetophenones as the starting material (Scheme 1). Chalcones (**1a**–**1i**) were prepared by dissolving methoxyacetophenone and ortho-, meta-, and parahydroxybenzaldehydes in a basic ethanol solution.

In the final stage, the target compounds (**4a**–**4i**) were synthesized by reacting chalcone derivatives with the acetyl-gypso compound (**3**). Initially, acetyl-gypso (**3**) and chalcone derivatives (**1a–1i**) were dissolved in dichloromethane under a nitrogen atmosphere. Subsequently, DMAP and DCC were added, leading to the formation of novel gypsogenin-COO-chalcone hybrid derivatives (**4a**–**4i**) (Scheme 3).

**Scheme 3:**
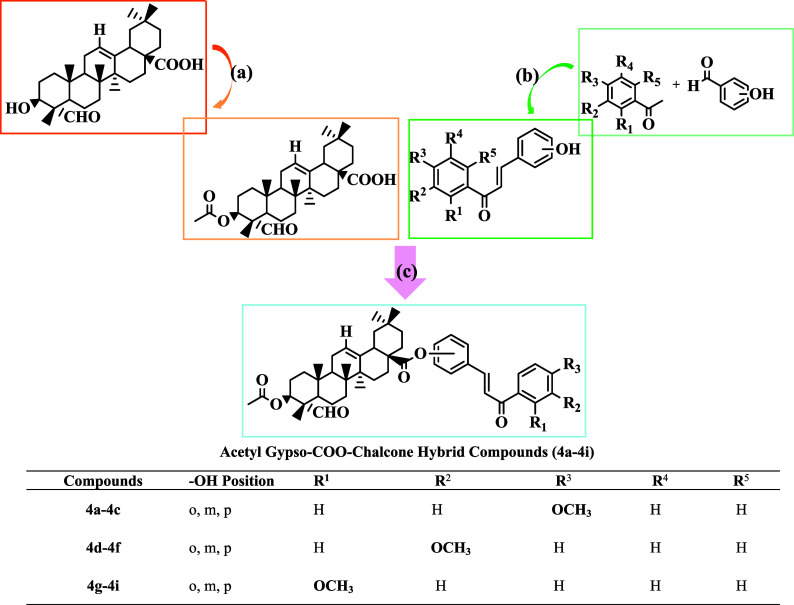
Synthesis of Acetyl Gypso–COO–Chalcone Hybrid Compounds^a^

a Reagents and conditions: (a) acetic anhydride, THF, RT; (b) substituted benzaldehydes, NaOH, EtOH; (c) DCC, DMAP, CH_2_Cl_2_, under N_2_.

The structures of these newly synthesized hybrid compounds were characterized using a range of spectroscopic techniques (^1^H NMR, ^13^C/APT-NMR, ^1^H–^1^H COSY-NMR, HMQC-NMR, FT-IR, and LC-MS/MS). The results were found to be consistent with the proposed structures.

In the obtained^1^ H NMR spectra, two doublet peaks observed in the range 7.5–8.5 ppm, with a coupling constant of approximately J*=* 16 Hz, are characteristic of the H-2 and H-3 protons of chalcones. These peaks correspond to an AB spin system, confirming the chalcone framework. Similarly, the most prominent peak in the ^13^C NMR spectra of these compounds corresponds to the quaternary carbonyl carbon of the *α,β-*unsaturated carbonyl skeleton, appearing around 190 ppm. This signal was consistently observed in all of the chalcone spectra, further validating their structures. Additionally, the intense stretching band detected in the FT-IR spectra in the range 1630–1650 cm^–1^, corresponding to the carbonyl group, provides further spectroscopic evidence supporting the structures. The −OH peak in the chalcone compounds appears as a broad singlet in the range of 5.0–6.0 ppm in the ^1^H NMR spectra. For some compounds, when deuterated methanol (CD_3_OD) was used as the solvent during spectrum acquisition, the −OH proton underwent deuterium exchange, resulting in reduced or completely absent OH signals in the ^1^H NMR spectra. Similarly, the stretching band at approximately 3330 cm^–1^ in the FT-IR spectra, associated with the −OH group, further supports the proposed structures.

In the second stage of the study, hydrolysis processes (basic and acidic hydrolysis) were applied to the *Gypsophila arrostii* root extract (aqueous extract of *Gypsophila arrostii* roots), followed by the isolation of the gypsogenin aglycone from the dichloromethane (DCM) phase. In the^1^ H NMR spectra of the gypsogenin aglycone, the peak corresponding to H-3 appears at approximately 5.5 ppm, H-12 at 5.7 ppm (brs), and H-18 at around 3.6 ppm as a dd peak. Additionally, the aldehyde peak of H-23 in gypsogenin aglycone is observed as a singlet around 9.7 ppm. In the APT spectra, the aldehyde carbon (C-23) appears at approximately 204 ppm, while C-12 at 122 ppm and C-3 at 74 ppm give peaks in the same direction as the solvent (CH_2_Cl_2_). Conversely, C-28 at approximately 183 ppm and C-13 at 143 ppm exhibit peaks in the opposite direction of the solvent. Also, the isolated gypsogenin aglycone (**2**) reacted with acetic anhydride and DMAP in THF to produce the acetyl-gypso compound (**3**). In the spectroscopic data for this compound, new proton peaks corresponding to a methyl group (CH_3_–COO) were observed in the ^1^H NMR spectra around 1.9–2.1 ppm. In the APT spectra, a carbon peak corresponding to the ester carbon (CH_3_–COO-Gypso) was observed at approximately 170 ppm, while a new methyl carbon peak (CH_3_–COO-Gypso) appeared at around 20–21 ppm.

In the third stage, the synthesized acetyl-gypso compound (**3**) was reacted with the obtained chalcone compounds (**1a**–**1i**) in the presence of DCC/DMAP under inert conditions in dry dichloromethane (DCM) to produce new semisynthetic derivatives. Based on the spectroscopic data of the newly synthesized derivatives, the peak observed at approximately 184 ppm in the APT spectra for the C-28 position of the starting Acetyl-Gypso compound shifted to 175 ppm following the esterification reaction. Furthermore, the disappearance of the free −OH peaks corresponding to the starting chalcones was evident in the ^1^H NMR spectra. All data from both chalcones and the Acetyl-Gypso compound were clearly observed in the ^1^H NMR spectra. Additionally, the sharp peaks in the FT-IR spectra in the frequency range of approximately 1650–1750 cm^–1^ confirmed the presence of C=O groups. In the LC-MS/MS spectra, the major peaks corresponded primarily to either [M]^+^ or [M+1]^+^ ions. According to the results, all structural analyses support the structures.

The reaction conditions yielded between 10% and 70% for all newly synthesized semisynthetic compounds. The incorporation of substituents at different positions in these derivatives led to notable differences in both the synthetic yield and biological performance.

### 2.2 Biological Activity

#### 2.2.1 Antimicrobial Activity

The minimum inhibitory concentration (MIC) determination was performed using the microdilution method^19^ for chalcones (**1a**–**1i**), gypsogenin (**2**), acetyl-gypso compound (**3**), and gypsogenin-COO-chalcone hybrid compounds (**4a**–**4i**). The antimicrobial activities of these compounds were assessed against different microbial strains. The analysis included three Gram-positive bacterial strains (*Bacillus subtilis* RSKK 02021, *Enterococcus faecalis* ATCC 29212, *Staphylococcus aureus* ATCC 29213), two Gram-negative strains (*Escherichia coli* ATCC 25922, *Pseudomonas aeruginosa* ATCC 27853), and one yeast strain (*Candida albicans* ATCC 90028). The results of the in vitro antimicrobial activities against five bacteria and one yeast of the tested semisynthetic compounds (**4a**–**4i**) were reported as MIC values and given in Table 1.

When the antimicrobial activity results presented in Table 1 were analyzed, it was observed that compounds **4b**, **4c**, **4d**, **4e**, **4f**, **4g**, **4h**, and **4i** inhibited the growth of Gram-positive bacteria at concentrations ranging from 16 to 256 μg/mL, whereas compounds **4a**, **4b**, **4c**, **4d**, and **4i** inhibited the growth of Gram-negative bacteria at concentrations between 256 and 512 μg/mL. These results indicate a preferential activity of the hybrid compounds toward Gram-positive bacteria. The newly synthesized hybrid compounds (**4a**–**4i**) did not exhibit significant activity against the yeast *Candida albicans* ATCC 90028.

Comparing the activities of the newly synthesized semisynthetic compounds with the parent gypsogenin aglycone, it was observed that compounds **4c** and **4f** showed significant selectivity toward Gram-positive bacteria, while compounds **4a**, **4b**, **4d**, **4e**, and **4i** were more effective against Gram-negative bacteria. According to the data obtained from the antimicrobial activity assays, the most effective compounds, **4c** and **4f**, inhibited the growth of Gram-positive bacteria at a concentration of 16 μg/mL.

When the antimicrobial activity was examined in relation to the chemical structures, it was observed that semisynthetic compounds exhibiting higher activity were associated with the presence of −OH groups at the meta and para positions of the chalcone structure.

#### 2.2.2 In Vitro Cytotoxicity

All synthesized chalcone derivatives (**1a**–**1i**) and the acetyl gypso-COO-chalcone hybrid compounds (**4a**–**4i**) were evaluated for their in vitro cytotoxic activities against five human cancer cell lines, including PANC-1 (human pancreatic cancer), MDA-MB-231 (human breast cancer), HeLa (human cervical cancer), A549 (human lung cancer), and SH-SY5Y (human neuroblastoma cancer), as well as the normal HEK293 cell line, using the MTT assay, with doxorubicin as the reference drug. The IC_50_ values expressed in micromolar units (μM) are summarized in Tables 2 and 3, and the comparative cytotoxic profiles are illustrated in Figure 1A and B.

**Figure 1:**
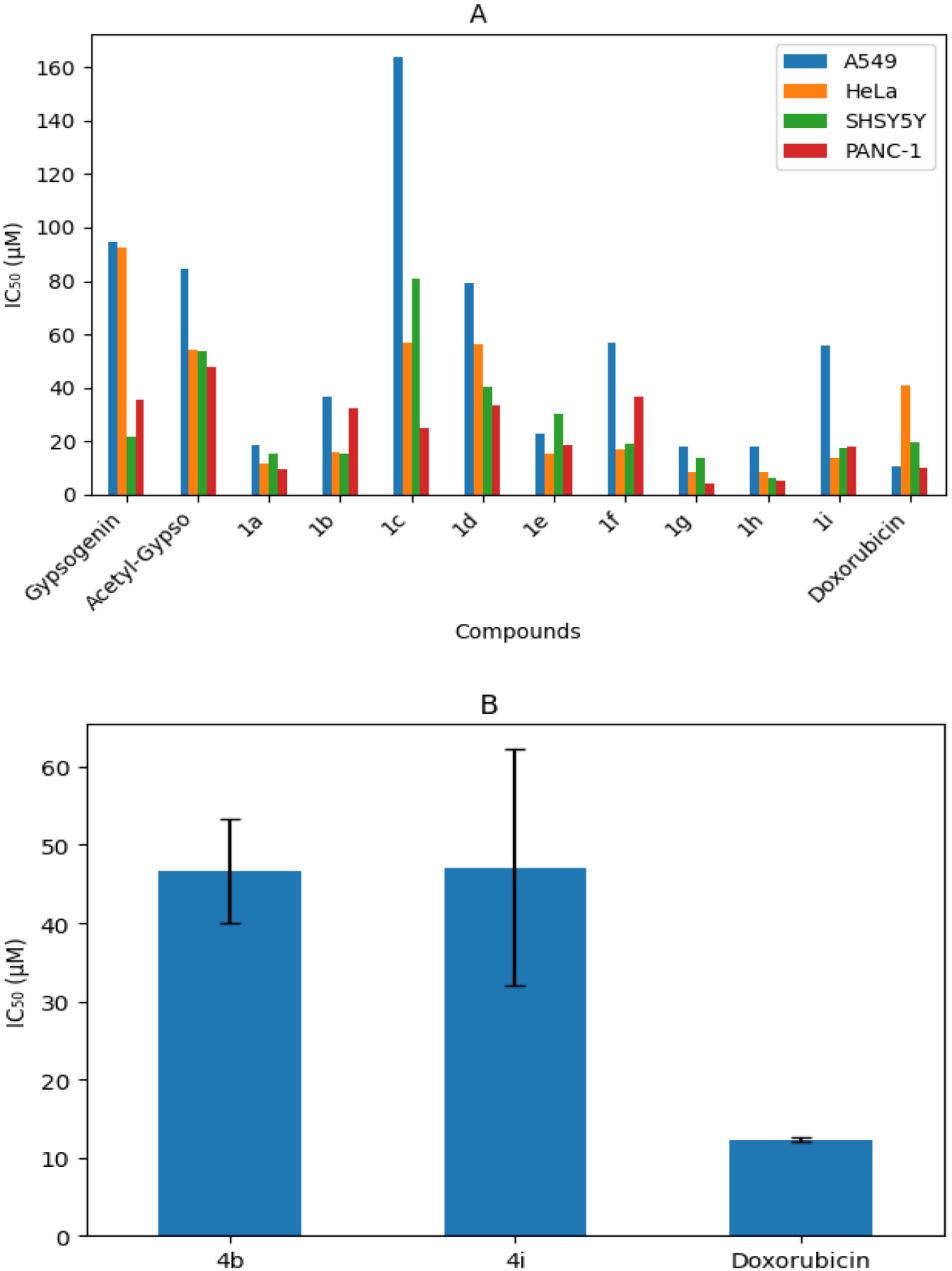
(A) IC_50_ values of the tested chalcone derivatives and control compounds against different cancer cell lines expressed in micromolar (μM). (B) IC_50_ values of the acetyl gypsogenin–chalcone hybrid compounds **4b** and **4i** against PANC-1 cells. Doxorubicin was used as a positive control.

**Table 2:** IC_50_ Values of Chalcone Compounds (**1a**–**1i**) Determined by the MTT Assay^a^

IC_50_(μM)						
Chalcones	A549	HeLa	SHSY5Y	MDA-MB231	PANC-1	HEK293
Gypsogenin	51.3 ± 12.7	50.2 ± 3.8	11.6 ± 1.1	19.3 ± 1.6	19.2 ± 1.1	33.5 ± 7.0
Acetyl-Gypso	42.1 ± 2.3	26.9 ± 5.1	26.7 ± 5.3	20.2 ± 3.8	23.8 ± 1.5	29.7 ± 0.4
**1a**	18.3 ± 2.9	11.6 ± 1.2	15.4 ± 2.8	35.3 ± 0.9	9.4 ± 1.5	8.5 ± 1.4
**1b**	36.5 ± 13.7	15.8 ± 1.7	15.6 ± 4.3	87.4 ± 0.4	32.6 ± 3.6	9.8 ± 1.6
**1c**	164.0 ± 23.8	56.7 ± 7.0	80.8 ± 1.1	96.0 ± 0.7	24.8 ± 1.9	60.3 ± 9.4
**1d**	79.4 ± 26.3	56.2 ± 4.4	40.4 ± 2.6	86.6 ± 4.8	33.2 ± 2.2	33.2 ± 8.5
**1e**	22.6 ± 3.1	15.6 ± 0.2	30.3 ± 16.8	37.5 ± 1.1	18.6 ± 1.6	18.0 ± 8.2
**1f**	57.1 ± 1.5	16.9 ± 0.9	19.1 ± 0.6	-	36.9 ± 1.7	30.2 ± 2.5
**1g**	17.9 ± 2.4	8.2 ± 0.2	13.5 ± 5.7	-	4.4 ± 0.2	7.8 ± 0.4
**1h**	17.8 ± 3.7	8.3 ± 2.0	6.5 ± 1.0	-	5.2 ± 0.4	6.6 ± 0.8
**1i**	55.9 ± 3.8	13.8 ± 1.3	17.6 ± 0.0	-	18.2 ± 1.0	32.7 ± 0.2
Doxorubicin	10.4 ± 1.7	40.9 ± 2.3	19.4 ± 1.4	15.8 ± 1.9	10.3 ± 2.2	4.6 ± 0.9

a Notes: A549 (human lung adenocarcinoma), HeLa (human cervical carcinoma), SH-SY5Y (human neuroblastoma), MDA-MB-231 (human breast adenocarcinoma), PANC-1 (human pancreatic carcinoma), and HEK293 (normal human embryonic kidney) cell lines were used.

**Table 3:** IC_50_ Values of Acetyl Gypso–COO–Chalcone Hybrid Compounds (**4a**–**4i**) Determined by the MTT Assay^a^

IC_50_(μM)						
Chalcones	A549	HeLa	SHSY5Y	MDA-MB231	PANC-1	HEK293
Gypsogenin	24.05 ± 1.89	19.99 ± 0.53	11.25 ± 1.43	32.82 ± 2.25	28.90 ± 2.43	16.19 ± 2.41
Acetyl-Gypso	7.50 ± 0.94	6.58 ± 0.39	3.79 ± 0.25	10.60 ± 3.85	9.88 ± 1.60	13.86 ± 0.55
**4a**	-	-	-	-	-	-
**4b**	-	-	-	-	46.59 ± 6.64	-
**4c**	-	-	-	-	-	-
**4d**	-	-	-	-	-	-
**4e**	-	-	-	-	-	-
**4f**	-	-	-	-	-	-
**4g**	-	-	-	-	-	-
**4h**	-	-	-	-	-	-
**4i**	-	-	-	-	47.14 ± 15.18	-
Doxorubicin	10.38 ± 1.69	40.85 ± 2.32	19.31 ± 1.40	31.17 ± 1.36	12.24 ± 0.28	9.94 ± 0.02

a Notes: A549 (human lung adenocarcinoma), HeLa (human cervical carcinoma), SH-SY5Y (human neuroblastoma), MDA-MB-231 (human breast adenocarcinoma), PANC-1 (human pancreatic carcinoma), and HEK293 (normal human embryonic kidney) cell lines were used.

As shown in Table 2 and Figure 1A, several chalcone derivatives displayed pronounced cytotoxic activity, with IC_50_ values in the low micromolar range. In particular, compound **1g** demonstrated strong antiproliferative effects against HeLa cells (IC_50_ = 8.2 ± 0.2 μM) and PANC-1 cells (IC_50_ = 4.4 ± 0.2 μM), while compound **1h** showed notable activity against HEK293 cells (IC_50_ = 6.6 ± 0.8 μM) and SH-SY5Y cells (IC_50_ = 6.5 ± 1.0 μM). Several other derivatives, including **1a**, **1b**, **1e**, **1f**, and **1i**, also displayed enhanced cytotoxic effects in HeLa and SH-SY5Y cell lines compared with those of doxorubicin. In PANC-1 cells, compounds **1a**, **1g**, and **1h** exhibited stronger antiproliferative activity than the reference drug, whereas none of the tested chalcones surpassed doxorubicin in the A549 or MDA-MB-231 cells. Doxorubicin remained more potent in the normal HEK293 cell line. It is noteworthy that compound **1a** has previously been synthesized and reported to exhibit anticancer activity, including against the A549 cell line.^20^ In the present study, compound **1a** showed cytotoxic activity across five different cancer cell lines in addition to the normal HEK293 cell line under identical experimental conditions, enabling a more comprehensive evaluation of its antiproliferative profile.

The acetyl gypsogenin–chalcone hybrid compounds (**4a**–**4i**) generally exhibited weak or negligible cytotoxic activity across the tested cancer cell lines (Table 3 and Figure 1B). Only compounds **4b** and **4i** showed measurable inhibitory effects against PANC-1 cells, while the remaining hybrids were largely inactive, indicating that the formation of acetyl gypsogenin–chalcone hybrid structures did not improve antiproliferative potency in this compound series.

Overall, the cytotoxicity results demonstrate that the chalcone structure plays a central role in the observed antiproliferative activity, whereas the incorporation of the acetyl gypsogenin unit led to a reduced biological efficacy.

Tumor selectivity was quantified as the Tumor Selectivity Index (TSI) relative to the normal HEK293 cell line, calculated from IC_50_ ratios. Gypsogenin showed pronounced selectivity toward SHSY5Y neuroblastoma cells (TSI = 2.88) and moderate selectivity in MDA-MB-231 and PANC-1 cells (TSI ≈ 1.7–1.8), indicating a favorable cancer-preferential cytotoxic profile. Among the synthetic derivatives, compound **1c** showed strong selectivity in PANC-1 cells (TSI = 2.43), while **1i** exhibited marked selectivity in HeLa cells (TSI = 2.36) and retained moderate selectivity in SHSY5Y and PANC-1 cells (TSI ≈ 1.8). Several additional compounds, including Acetyl-Gypsogenin and **1f**, displayed intermediate selectivity (TSI = 1.1–1.8) in selected cancer cell lines, whereas most compounds showed limited selectivity in A549 cells (TSI < 1). In contrast, doxorubicin exhibited TSI values consistently below 1 across all tested cancer cell lines, indicating a lack of tumor selectivity relative to that of HEK293 cells (Table 4).

**Table 4:** Tumor Selectivity Index (TSI) Values of Selected Compounds Calculated from IC_50_ Ratios Between Cancer Cell Lines and the Normal HEK293 Cell Line^a^

TSI value	A549	HeLa	SHSY5Y	MDA-MB231	PANC-1
Gypsogenin	0.65	0.67	**2.88**	**1.73**	**1.75**
Acetyl-Gypso	0.71	**1.10**	**1.11**	**1.47**	**1.25**
**1a**	0.47	0.74	0.55	0.24	0.90
**1b**	0.27	0.62	0.63	0.11	0.30
**1c**	0.37	**1.06**	0.75	0.63	**2.43**
**1d**	0.42	0.59	0.82	0.38	**1.00**
**1e**	0.79	**1.15**	0.59	0.48	0.97
**1f**	0.53	**1.78**	**1.58**	–	0.82
**1g**	0.44	0.95	0.58	–	**1.79**
**1h**	0.37	0.79	**1.01**	–	**1.28**
**1i**	0.59	**2.36**	**1.86**	–	**1.79**
Doxorubicin	0.44	0.11	0.24	0.29	0.45

a Notes: TSI values were calculated as the ratio of IC_50_ values in the normal HEK293 cell line to those in the corresponding cancer cell lines. TSI values were calculated only for compounds showing measurable IC_50_ values in cytotoxicity assays.

### 2.3 Molecular Docking Studies

To investigate possible interaction modes between compounds **1g** and **1h** and multiple cancer-related targets, including the epidermal growth factor receptor (EGFR), Akt-1, PI3K, human estrogen receptor, tubulin, and vascular endothelial growth factor receptor (VEGFR-2) (PDB IDs: 4HJO,^21^ 3MV5,^22^ 5XGJ,^23^ 3ERT,^24^ 1SA0,^25^ 4ASD^26^), molecular docking studies were performed using the Schrödinger Maestro 14.1 software.^27^ In addition, molecular docking analyses were used to compare the interactions of **1g** and **1h** with the reference drug doxorubicin (Table 5).

**Table 5:** Docking Results of Compounds **1g**, **1h**, and Doxorubicin at Their Binding Sites against the Predicted Targets^a^

Target	PDB ID	RMSD	Compounds	Docking Score	Glide emodel	Glide Energy
EGFR Tyrosine Kinase	4HJO	0.137	**1g**	–7.010	–54.206	–38.921
			**1h**	–7.276	–57.503	–41.858
			Doxorubicin	–7.181	–72.405	–54.129
Akt-1	3MV5	0.130	**1g**	–4.932	–44.827	–36.963
			**1h**	–5.707	–46.186	–34.338
			Doxorubicin	–5.386	–82.429	–61.433
PI3K	5XGJ	0.100	**1g**	–6.674	–49.378	–36.102
			**1h**	–6.220	–52.843	–38.828
			Doxorubicin	–6.525	–67.828	–46.656
Human Estrogen Receptor	3ERT	0.129	**1g**	–6.622	–47.759	–35.591
			**1h**	–8.405	–50.157	–40.058
			Doxorubicin	–6.645	–71.194	–52.985
Tubulin	1SA0	0.143	**1g**	–6.201	–48.787	–36.207
			**1h**	–7.175	–52.684	–37.678
			Doxorubicin	–5.933	–57.382	–44.141
VEGFR-2	4ASD	0.100	**1g**	–8.196	–55.097	–37.694
			**1h**	–8.659	–57.466	–39.592
			Doxorubicin	–5.603	–49.508	–54.480

a Notes: Glide emodel and Glide energy values calculated on the 4HJO crystal structure in Table 5 are −54.206 and −38.921 kcal/mol for 1g, −57.503 and −41.858 kcal/mol for **1h**, −72.405 and −54.129 kcal/mol for doxorubicin. The 2D diagram of the interaction of these three compounds with the crystal structure of EGFR is shown in Figure 2. In Figure 2B, **1h**, which has the best binding mode with 4HJO, interacted with the Asp831 amino acid residue via the −OH in the compound and with Met769, one of the important amino acids, via the = O group via hydrogen bonding.

Given that anticancer activity may involve multiple molecular targets, six different targets were selected for the molecular docking analysis to better rationalize the experimental cytotoxicity results. During target selection, crystal structures relevant to signaling pathways associated with the tested cancer cell lines were considered.

Compounds **1g** and **1h**, which exhibited the most pronounced in vitro activity, were therefore evaluated in silico against the selected protein targets. For comparison purposes, the docking results of doxorubicin were also calculated.

As the first docking target, EGFR, a key regulator of cancer cell proliferation and survival, was evaluated, and binding parameter values were calculated. When the initial data in Table 5 were examined, compound **1h** showed a docking result slightly more favorable than that of doxorubicin. The reference drug for EGFR, doxorubicin, has a docking score of −7.181 kcal/mol, while the value for **1h** is −7.276 kcal/mol. It can be said that **1h** has a better value in terms of theoretical binding.

Glide emodel and Glide energy values calculated on the 4HJO crystal structure in Table 4 are −54.206 and −38.921 kcal/mol for **1g**; −57.503 and −41.858 kcal/mol for **1h**; and −72.405 and −54.129 kcal/mol for doxorubicin. The two-dimensional interaction diagrams of these three compounds with the crystal structure of EGFR are shown in Figure 2. In Figure 2B, compound **1h**, which has the best binding mode with 4HJO, interacts with the Asp831 amino acid residue via −OH in the compound and with Met769, one of the important amino acids, via the =O group through hydrogen bonding. Figure 2C presents the 2D interaction diagram of doxorubicin used as a reference in molecular docking analysis. Figure 2C shows hydrogen bonds between the amino acid residue Asp831 and amino acid NH_3_, between the amino acid residue Arg817 and amino acid −OH, and between the residue Asn818 and the −NH_3_ group. Furthermore, the compound doxorubicin exhibits strong hydrophobic interactions. Figure 2B highlights the interaction features responsible for the improved binding of compound **1h** to the EGFR target.

**Figure 2:**
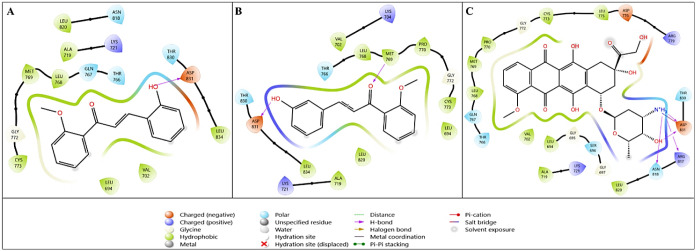
Two-dimensional interaction diagrams of lead compounds (A) **1g** and (B) **1h** and the reference compound (C) doxorubicin with the EGFR target (PDB ID: 4HJO).

Compound **1h** showed high binding affinity in the binding pocket of Akt-1 (PDB ID: 3MV5). It was determined that compound **1h** interacted with amino acid residues better than doxorubicin did in the binding site of 3MV5 (Figure 3). The docking results of this interaction showed that compound **1h** had a high docking interaction value of −5.707 kcal/mol, while the reference compound doxorubicin was calculated as −5.386 kcal/mol and **1g** as −4.932 kcal/mol.

**Figure 3:**
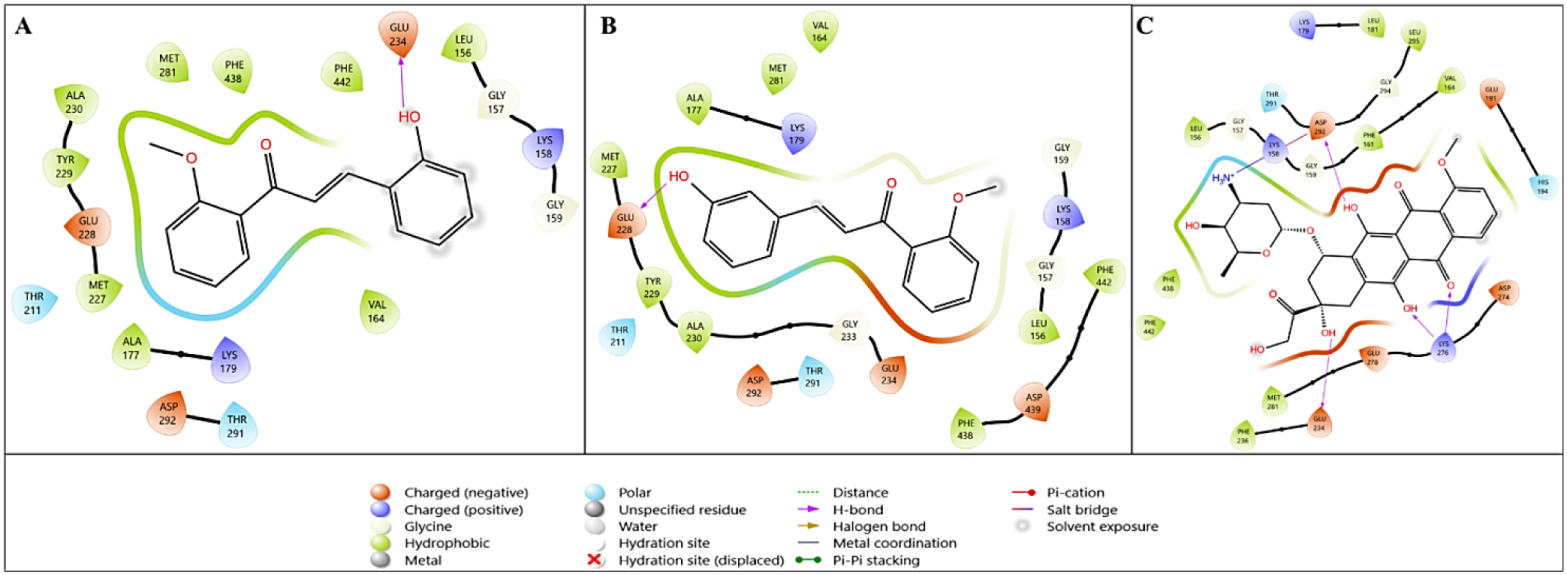
Two-dimensional interaction diagrams of lead compounds (A) **1g** and (B) **1h** and the reference compound (C) doxorubicin with the Akt-1 target (PDB ID: 3MV5).

The interaction diagram of compound **1h**, which best approaches the active pocket region of the crystal structure of Akt-1, is shown in Figure 3B. Among compounds **1g**, **1h**, and doxorubicin, compound **1h** exhibits the most favorable interaction with the Akt-1 target. Compound **1h**, which shows a stronger binding profile than the reference compound doxorubicin, interacts with the active pocket region through hydrogen bonding between its −OH group and the key amino acid Glu228, as well as through hydrophobic, charged, and polar interactions with Glu234, Tyr229, Gly157, and Met227 (Figure 3B). A detailed examination of the 2D interaction diagram of doxorubicin with the 3MV5 crystal structure reveals a salt bridge interaction between Asp292 and the −NH_3_ group, along with hydrogen bonding interactions involving Lys276 (−OH and =O atoms) and Glu234.

Another target that is active in cancer cells is PI3K. PI3K crystal structure (5XGJ) compounds **1g**, **1h**, and reference drug doxorubicin were interacted *in silico*, respectively. Numerical values of this interaction are presented in Table 5, and the visualized modes are presented in Figure 4. Table 3 shows that compound **1g** has a better binding score than the reference compound doxorubicin on 5XGJ. For this target, **1g** has a docking score of −6.674, a Glide emodel value of −49.378, and a Glide energy value of −36.102 kcal/mol, while these values for doxorubicin are −6.525, −67.828, and −46.656 kcal/mol, respectively. When Figure 4 is examined in detail, it is determined that both compounds **1g** and **1h** are properly placed in the active pocket region of the crystal structure of PI3K. Figure 4A shows that hydrogen bonds are formed between three different amino acids (Asp810, Tyr836, and Asp933) via the −OH group in compound **1g**, and a π–π bond interaction occurs with the amino acid Trp780 via the phenyl group. Figure 4B shows the diagrammatic interaction of compound **1h** with the crystal structure of PI3K (PDB ID: 5XGJ). Figure 4B shows that compound **1h** is in the active pocket region of the 5XGJ crystal structure, exhibits hydrogen bonding interactions with Tyr836 and Asp810 via the −OH group, and also has hydrophobic interactions in the active pocket region. Figure 4C presents the interaction between the reference compound and the PI3K target.

**Figure 4:**
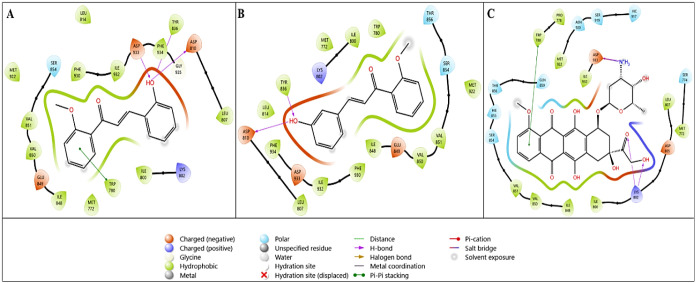
Two-dimensional interaction diagrams of lead compounds (A) **1g** and (B) **1h,** and the reference compound. (C) Doxorubicin with the PI3K target (PDB ID: 5XGJ).

Compounds that may be active with the human estrogen receptor (PDB ID: 3ERT) were evaluated by molecular docking as compound **1g** and compound **1h**. As a result of this interaction, the docking score for compound **1g** was −6.622 kcal/mol, for compound **1h**, it was −8.405 kcal/mol, and for doxorubicin, it was −6.645 kcal/mol. As determined in Table 5, compound **1h** has a higher binding affinity for the human estrogen receptor. The visualization of this calculated result is presented in Figure 5. Figure 5A presents the interaction diagram for compound **1g**, Figure 5B shows the interaction diagram for compound **1h**, and Figure 5C shows the interaction diagram for the reference compound doxorubicin. The ligand **1g**, which is positioned in the active pocket region of the estrogen receptor target, exhibits intense hydrophobic and polar interactions, while also interacting with the Thr347 amino acid residue via hydrogen bonding through the −OH group (Figure 5A). The compound **1h**, which has a strong hydrophobic interaction in Figure 5B, has a hydrogen bond interaction with the amino acid residues Arg394 and Glu353 via the −OH group.

**Figure 5:**
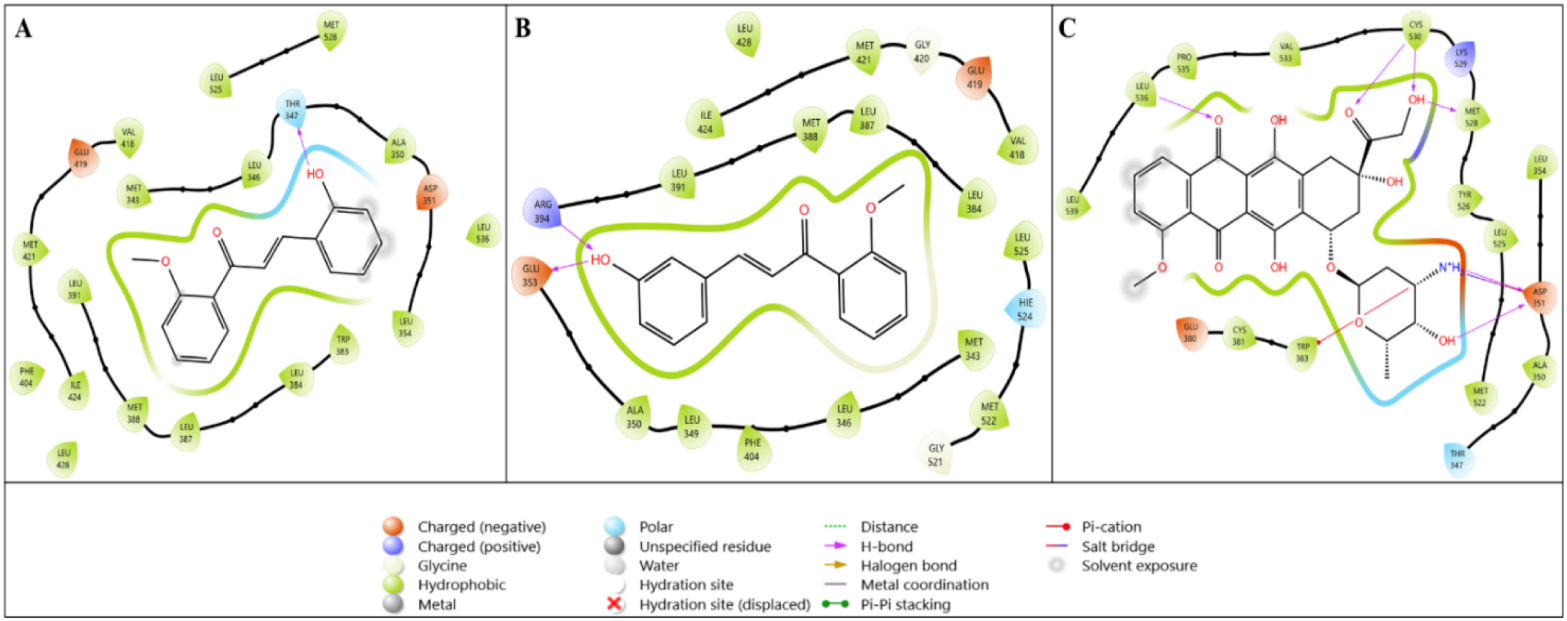
Two-dimensional interaction diagrams of lead compounds (A) **1g** and (B) **1h** and the reference compound. (C) Doxorubicin with the tubulin target (PDB ID: 1SA0).

Table 5 presents the binding parameter value results of the compounds interacting with the crystal structure of tubulin, which was determined as the other target. What is striking here is that both **1g** and **1h** have better binding scores than the reference doxorubicin. In Table 5, the binding interaction values with the 1SA0 crystal structure, docking score, Glide emodel, and Glide energy values were calculated as −6.201, −48.787, and −36.207 for **1g**; −7.175, −52.684, and −37.678 for **1h,** and −5.933, −57.382, and −44.141 kcal/mol for doxorubicin, respectively. It was determined that compound **1h** had the best binding value, while the values of compound **1g** were calculated to be close to it.

The two-dimensional interaction diagrams corresponding to these binding parameter values are presented in Figure 6. In Figure 6A, it was determined that compound **1g** fits perfectly into the 1SA0 crystal structure and exhibits a hydrophobic interaction. In Figure 6B, compound **1h** fits into the active pocket region and forms a hydrogen bond with the Val315 amino acid residue via the −OH group. Figure 6C presents the interaction of the reference compound doxorubicin with the tubulin target. The reference compound forms hydrogen bonds with the Thr753 amino acid residue via the −OH group and with the Asp329 amino acid residue via the −NH_3_ and −OH groups.

**Figure 6:**
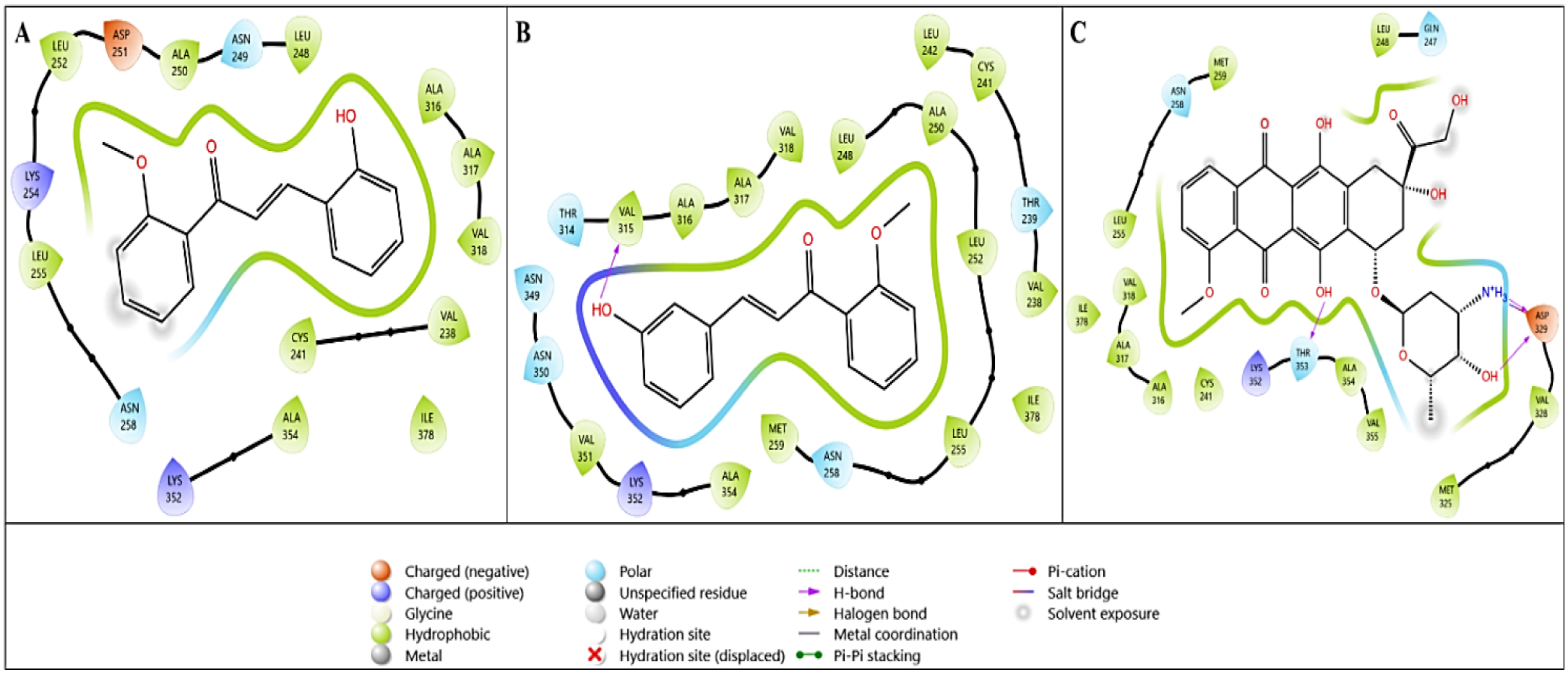
Two-dimensional interaction diagrams of lead compounds (A) **1g** and (B) **1h** and the reference compound (C) doxorubicin with the tubulin target (PDB ID: 1SA0).

Since VEGFR-2 overexpression is seen in various types of cancer, such as breast cancer, cervical cancer, nonsmall cell lung cancer, hepatocellular carcinoma, and kidney carcinoma, it is important to examine the crystal structure of this target in silico approaches.^28^

As the last target in Table 5, therefore, the interactions of VEGFR-2 on compounds **1g**, **1h**, and doxorubicin were analyzed. The docking score of compound **1g**, which interacted with 4ASD determined as the crystal structure of VEGFR-2, was −8.196 kcal/mol, the docking score of **1h** was −8.659 kcal/mol, and this value was lower for doxorubicin −5.603 kcal/mol. When these values were analyzed, it is shown in Table 5 that both compounds had better binding scores than doxorubicin.

The interaction diagrams of compounds **1g** and **1h**, which exhibit improved binding interactions with VEGFR-2 in silico, are presented in Figures 7A and B. Detailed examination of the 2D diagrams indicates that both compounds display hydrophobic and charged (electrostatic) interactions. In Figure 7A, compound **1g** forms a π-cation interaction with the Lys868 amino acid residue via the phenyl ring and a hydrogen bond with Glu917 through the −OH group. In Figure 7B, compound **1h** forms a hydrogen bond with the key amino acid residue Cys919 via the =O group.

**Figure 7:**
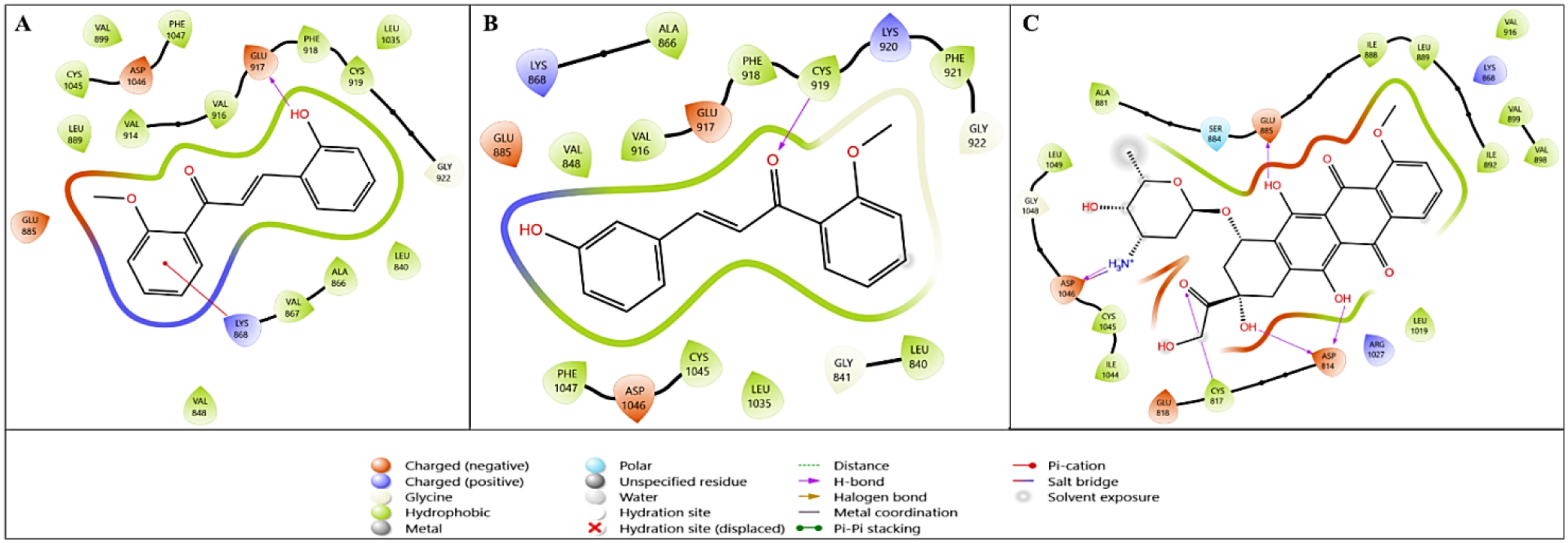
Two-dimensional interaction diagrams of lead compounds (A) **1g** and (B) **1h** and the reference compound (C) doxorubicin with the VEGFR-2 target (PDB ID: 4ASD).

Figure 7C illustrates the interaction of the reference compound with the 4ASD crystal structure, showing hydrogen bonding with Asp814 and Glu885 via −OH groups, with Cys817 via the =O atom, and with Asp1046 via the −NH_3_ group. The observed binding parameter values are closely related to the corresponding binding modes. Comparative analysis of both docking scores and interaction diagrams of the ligands and reference compounds is essential for understanding target–ligand interactions. Accordingly, each target was evaluated in detail in terms of both numerical affinity and binding mode.

## 3 Conclusion

In summary, a series of methoxy-substituted chalcone derivatives and acetyl gypsogenin-based hybrid compounds were successfully synthesized and evaluated for their anticancer and antimicrobial activities. Several chalcone derivatives, particularly compounds **1g** and **1h**, exhibited pronounced cytotoxic effects against selected cancer cell lines, including PANC-1 and MDA-MB-231, while showing reduced toxicity toward the normal HEK293 cell line, indicating a favorable selectivity profile. In contrast, the acetyl gypsogenin–chalcone hybrid compounds generally displayed weak or negligible cytotoxic activity under the tested conditions. Moderate antimicrobial activity was observed for some compounds, highlighting the contribution of the chalcone scaffold to the biological effects of this series. Molecular docking studies further supported the experimental findings by revealing plausible interactions of the most active chalcones with cancer-related molecular targets. Overall, the results demonstrate that the chalcone group plays a central role in the observed biological activity, while hybridization with acetyl gypsogenin did not enhance the antiproliferative potency in this study. These findings provide valuable structure–activity relationship insights that may guide future optimization of chalcone-based compounds.

## 4 Experimental Section

### 4.1 Chemistry

#### 4.1.1 General

For all compounds, melting points (mp) were detected using a Gallen-kamp electrothermal melting point apparatus (uncorrected ± 0.1 °C). Infrared spectra were measured on a PerkinElmer Frontier FT/IR spectrometer. LC-MS was recorded on a Thermo Scientific/Surveyor MSQ spectrometer and the electrospray ionization (ESI) method. ^1^H NMR (600 MHz) and ^13^C NMR (150 MHz) spectra were recorded on a Bruker spectrometer. Solvents used for NMR spectra acquisition included CDCl_3_, CD_3_OD, pyridine-d_5_, and DMSO-*d*_6_. Chemical shifts are reported in ppm relative to tetramethylsilane (TMS) (0 ppm) as a reference. Data are reported as chemical shifts, multiplicity (s = singlet, d = doublet, t = triplet, q = quartet, m = multiplet). Samples were placed in quartz NMR tubes for measurement.

All solvents used were dried and distilled prior to use. Column chromatography was performed by using 60 Å silica gel (Merck 7734). The progress of all chemical reactions was monitored by thin-layer chromatography (TLC), and TLC was performed using 60 Å silica gel on F254 aluminum plates (Merck 5554).

#### 4.1.2 General Procedure for the Synthesis of Compounds (**1a**–**1i**)

The synthetic procedure of methoxy-substituted chalcones is figured in Scheme 1. Chalcone compounds (**1a**–**1i**) were synthesized by base-catalyzed Claisen–Schmidt condensation^29^ using appropriately substituted acetophenone with commercially available benzaldehyde. A mixture of methoxy-acetophenone derivative (10 mmol) and hydroxy-benzaldehyde derivative (10 mmol) was dissolved in ethanol and 40% alcoholic NaOH (15 mL) was added and was stirred at 5–10 °C by using an ice bath. The progress of the reaction was monitored by TLC. Thereafter, after completion of reaction, the reaction mixture was poured into ice water and acidified with dilute hydrochloric acid. The precipitated solid was filtered, washed thoroughly with distilled water, and recrystallized from aqueous ethanol.

#### 4.1.3 Characterization of Compounds (**1a**–**1i**)

The atom numbering used for NMR analysis of compounds (**1a**–**1i**) is shown in Scheme 4.

**Scheme 4:**
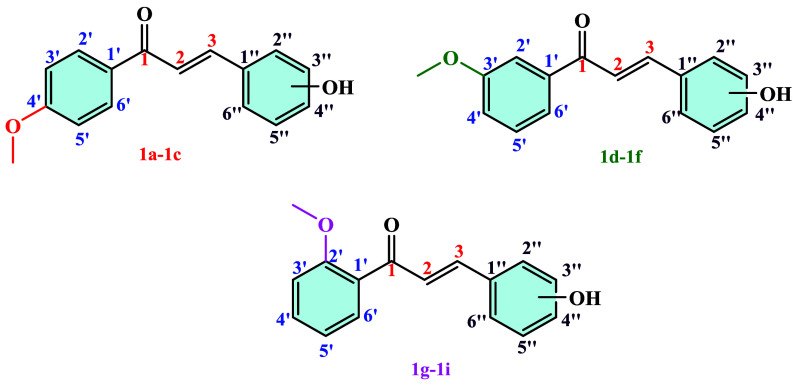
Numbering of Atoms of Compound **1a**–**1i** for NMR Analysis

##### 4.1.3.1 (2E)-3-(2-Hydroxyphenyl)-1-(4-methoxyphenyl)­prop-2-en-1-one (**1a**)

Rf: 0.54 (Hexane-Diethyl ether: 1:3); Yield: 82%; mp 145–149 °C; FT-IR KBr (cm^–1^): 3220, 3008, 2972, 1641, 1595, 1582, 1559, 1457, 1340, 751;

^1^H NMR (400 MHz, CDCl_3_/CD_3_OD) δ 8.10 (d, J = 16.0 Hz, 1H), 8.00 (d, *J =* 8.0 Hz, 1H), 8.00 (d, *J =* 8.0 Hz, 1H), 7.80 (d, *J =* 16.0 Hz, 1H), 7.60 (d, *J =* 8.0 Hz, 1H), 7.20 (t, *J =* 8.0 Hz, 1H), 7.00 (d, *J =* 8.0 Hz, 2H), 6.90 (d, *J =* 8.0 Hz, 1H), 6.80 (t, *J =* 8.0 Hz, 1H), 3.90 (s, 3H).

^13^C NMR (100 MHz, CD_3_OD) δ: 190.6, 163.8, 157.4, 140.3, 131.5, 130.9, 130.6, 130.6, 129.0, 121.9, 121.1, 119.5, 115.7, 113.6, 54.6. LC-MS/MS (positive mode) *m*/*z*: 304 [M–OCH_3_+2K+3]^+^, 237 [M–OH]^+^.

##### 4.1.3.2 (2E)-3-(3-Hydroxyphenyl)-1-(4-methoxyphenyl)­prop-2-en-1-one (**1b**)

Rf: 0.50 (Hexane-Diethyl ether: 1:3); Yield: 91%; mp 159–162 °C; FT-IR KBr (cm^–1^): 3323, 2969, 1650, 1583, 1563, 1264, 1166, 975, 830, 768;

^1^H NMR (400 MHz, CDCl_3_/CD_3_OD) δ 8.00 (d, *J =* 8.0 Hz, 1H), 8.00 (d, *J =* 8.0 Hz, 1H), 7.70 (d, *J =* 16.0 Hz, 1H), 7.50 (d, *J =* 16.0 Hz, 1H), 7.20 (t, *J =* 8.0 Hz, 1H), 7.10 (d, *J =* 8.0 Hz, 1H), 7.00 (s, 1H), 6.90 (d, *J =* 8.0 Hz, 2H), 6.80 (dd, *J =* 8.0, 1.4 Hz, 1H), 3.90 (s, 3H).

^13^C NMR (100 MHz, CD_3_OD) δ: 189.6, 163.6, 157.2, 144.6, 136.2, 130.9, 130.9, 130.8, 129.9, 121.8, 120.0, 117.7, 114.9, 113.9, 55.4. LC-MS/MS (positive mode) *m*/*z*: 304 [M–OCH_3_+2K+3]^+^, 255 [M + H]^+^, 171 [M–methoxyphenyl+Na]^+^, and 168 [M–(methoxyphenyl+OH)+K]^+^.

##### 4.1.3.3 (2E)-3-(4-Hydroxyphenyl)-1-(4-methoxyphenyl)­prop-2-en-1-one (**1c**)

Rf: 0.46 (Hexane-Diethyl ether: 1:3); Yield: 89%; mp 180–183 °C; FT-IR KBr (cm^–1^): 3153, 2967, 1642, 1599, 1578, 1553, 1512, 1435, 1343, 1223, 1164, 1020, 827, 751;

^1^H NMR (400 MHz, CDCl_3_/CD_3_OD) δ 8.10 (d, *J =* 8.0 Hz, 2H), 7.74 (d, *J =* 16.0 Hz, 1H), 7.61 (d, *J =* 16.0 Hz, 1H), 7.61 (d, *J =* 8.0 Hz, 2H), 7.07 (d, *J =* 8.0 Hz, 2H), 6.86 (d, *J =* 8.0 Hz, 2H), 3.91 (s, 3H).

^13^C NMR (100 MHz, CD_3_OD) δ: 189.4, 163.8, 160.3, 144.7, 130.9, 130.5, 130.5, 130.3, 126.5, 118.2, 115.7, 115.7, 113.6, 54.6. LC-MS/MS (positive mode) *m*/*z*: 256 [M+2]^+^, 255 [M + H]^+^, 246 [M–OCH_3_+Na]+, 238 [M–OH+H]^+^, 171 [M–methoxyphenyl+Na]^+^, 168 [M–methoxyphenyl+K]^+^.

##### 4.1.3.4 (2E)-3-(2-Hydroxyphenyl)-1-(3-methoxyphenyl)­prop-2-en-1-one (**1d**)

Rf: 0.52 (Hexane-Diethyl ether: 1:3); Yield: 71%; mp 110–113 °C; FT-IR KBr (cm^–1^): 3280, 2968, 1640, 1595, 1573, 1550, 1510, 1438, 1023, 823, 750;

^1^H NMR (400 MHz, CDCl_3_/CD_3_OD) δ 8.21 (d, *J =* 16.0 Hz, 1H), 7.70 (d, *J =* 16.0 Hz, 1H), 7.65 (d, *J =* 8.0 Hz, 1H), 7.62 (d, *J =* 8.0 Hz, 1H), 7.60 (s, 1H), 7.44 (t, *J =* 8.0 Hz, 1H), 7.30 (t, *J =* 8.0 Hz, 1H), 7.20 (dd, *J =* 8.0, 1.6 Hz, 1H), 6.99 (d, *J =* 8.0 Hz, 1H), 6.96 (t, *J =* 8.0 Hz, 1H), 6.78 (br s, 1H), 3.91 (s, 3H).

^13^C NMR (100 MHz, CD_3_OD) δ 191.7, 159.9, 156.0, 141.0, 139.7, 131.8, 129.5, 129.5, 122.9, 122.2, 121.7, 120.9, 119.3, 116.6, 113.1, 55.5. LC-MS/MS (positive mode): *m*/*z*: 255 [M + H]^+^, 254 [M]^+^, 238 [M–OH+H]^+^, 237 [M–OH]^+^, 171 [M–methoxyphenyl+Na]^+^.

##### 4.1.3.5 (2E)-3-(3-Hydroxyphenyl)-1-(3-methoxyphenyl)­prop-2-en-1-one (**1e**)

Rf: 0.54 (Hexane-Diethyl ether: 1:3); Yield: 82%; mp 94 – 97 ^○^C; FT-IR KBr (cm^–1^): 3277, 2943, 1658, 1594, 1581, 1449, 1426, 1252, 1030, 878, 776;

^1^H NMR (400 MHz, CDCl_3_/CD_3_OD) δ 7.75 (d, *J =* 16.0 Hz, 1H), 7.64 (d, *J =* 8.0 Hz, 1H), 7.61 (d, *J =* 16.0 Hz, 1H), 7.54 (s, 1H), 7.44 (t, *J =* 8.0 Hz, 1H), 7.25 (t, *J =* 8.0 Hz, 1H), 7.19 (d, *J =* 8.0 Hz, 1H), 7.17 (d, *J =* 8.0 Hz, 1H), 7.13 (s, 1H), 6.88 (d, *J =* 8.0 Hz, 1H), 3.86 (s, 3H).

^13^C NMR (100 MHz, CD_3_OD) δ: 190.8, 160.1, 157.7, 145.1, 139.3, 136.1, 129.7, 129.5, 121.6, 120.8, 119.9, 118.9, 117.6, 114.5, 112.7, 54.5. LC-MS/MS (positive mode) *m*/*z*: 255 [M + H]^+^, 247 [M–OCH_3_+Na]^+^, 171 [M–methoxyphenyl+Na]^+^, 168 [M–methoxyphenyl+K]^+^, 148 [M–methoxyphenyl+H]^+^, 135 [methoxybenzoyl]^+^.

##### 4.1.3.6 (2E)-3-(4-Hydroxyphenyl)-1-(3-methoxyphenyl)­prop-2-en-1-one (**1f**)

Rf: 0.50 (Hexane-Diethyl ether: 1:3); Yield: 77%; mp 139–143 ^○^C; FT-IR KBr (cm^–1^): 3112, 3001, 2929, 1645, 1555, 1511, 1434, 1251, 1030, 832, 740;

^1^H NMR (400 MHz, CDCl_3_/CD_3_OD) δ 7.72 (d, *J =* 16.0 Hz, 1H), 7.53 (d, *J =* 8.0 Hz, 1H), 7.49 (d, *J =* 8.0 Hz, 2H), 7.46 (s, 1H), 7.36 (t, *J =* 8.0 Hz, 1H), 7.31 (d, *J =* 16.0 Hz, 1H), 7.08 (dd, *J =* 8.0, 2.0 Hz, 1H), 6.83 (d, *J =* 8.0 Hz, 2H), 3.83 (s, 3H).

^13^C NMR (100 MHz, CD_3_OD) δ 191.2, 159.8, 159.8, 145.8, 139.9, 130.6, 129.5, 126.4, 120.9, 119.0, 119.0, 115.9, 112.9, 55.4. LC-MS/MS (positive mode) *m*/*z*: 304 [M–OCH_3_+2K+3]^+^.

##### 4.1.3.7 (2E)-3-(2-Hydroxyphenyl)-1-(2-methoxyphenyl)­prop-2-en-1-one (**1g**)

Rf: 0.46 (Hexane-Diethyl ether: 1:3); Yield: 65%; mp 109–112 ^○^C; FT-IR KBr (cm^–1^): 3261, 3078, 2944, 1644, 1599, 1563, 1448, 1246, 1018, 746;

^1^H NMR (400 MHz, CDCl_3_/CD_3_OD) δ 7.90 (d, *J =* 16.0 Hz, 1H), 7.56 (d, *J =* 6.8 Hz, 1H), 7.51 (t, *J =* 8.0 Hz, 1H), 7.50 (d, *J =* 8.0 Hz, 1H), 7.44 (d, *J =* 16.0 Hz, 1H), 7.23 (t, *J =* 8.0 Hz, 1H), 7.13 (d, *J =* 8.0 Hz, 1H), 7.05 (t, *J =* 8.0 Hz, 1H), 6.87 (t, *J =* 6.8 Hz, 1H), 6.86 (d, *J =* 8.0 Hz, 1H), 3.89 (s, 3H).

^13^C NMR (100 MHz, CD_3_OD) δ 195.3, 158.1, 157.3, 140.4, 137.6, 131.5, 129.4, 129.2, 128.6, 126.3, 121.8, 120.3, 119.5, 115.7, 111.7, 54.8. LC-MS/MS (positive mode) *m*/*z*: 304 [M–OCH_3_+2K+3]^+^, 237 [M–OH]^+^.

##### 4.1.3.8 (2E)-3-(3-Hydroxyphenyl)-1-(2-methoxyphenyl)­prop-2-en-1-one (**1h**)

Rf: 0.65 (Hexane-Diethyl ether: 1:4); Yield: 82%; mp 96–99 °C; FT-IR KBr (cm^–1^): 3387, 3011, 2949, 1630, 1594, 1451, 1234, 972, 857, 760;

^1^H NMR (400 MHz, CDCl_3_/CD_3_OD) δ 7.55–7.50 (m, 2H), 7.49 (d, *J =* 16.0 Hz, 1H), 7.34 (d, *J =* 16.0 Hz, 1H), 7.23 (t, *J =* 8.0 Hz, 1H), 7.12 (d, *J =* 8.0 Hz, 1H), 7.07 (d, *J =* 8.0 Hz, 1H), 7.06 (s, 1H), 7.04 (t, *J =* 8.0 Hz, 1H), 6.82 (dd, *J =* 8.0, 2.4 Hz, 1H), 3.89 (s, 3H).

^13^C NMR (100 MHz, CD_3_OD) δ 194.0, 158.3, 157.7, 157.7, 143.8, 136.2, 133.0, 129.7, 129.7, 128.8, 126.5, 120.4, 119.9, 117.5, 114.1, 54.9. LC-MS/MS (positive mode) *m*/*z*: 304 [M–OCH_3_+2K+3]^+^.

##### 4.1.3.9 (2E)-3-(4-Hydroxyphenyl)-1-(2-methoxyphenyl)­prop-2-en-1-one (**1i**)

Rf: 0.58 (Hexane-Diethyl ether: 1:3); Yield: 69%; mp 133136 °C; FT-IR KBr (cm^–1^): 3251, 3092, 1643, 1594, 1560, 1011, 983, 834, 751;

^1^H NMR (400 MHz, CDCl_3_/CD_3_OD) δ 7.54–7.49 (m, 2H), 7.51 (d, *J =* 8.0 Hz, 2H), 7.50 (d, *J =* 16.0 Hz, 1H), 7.21 (d, *J =* 16.0 Hz, 1H), 7.15 (d, *J =* 8.0 Hz, 1H), 7.06 (t, *J =* 8.0 Hz, 1H), 6.84 (d, *J =* 8.0 Hz, 2H), 3.90 (s, 3H).

^13^C NMR (100 MHz, CD_3_OD) δ: 194.6, 160.2, 158.0, 144.9, 132.5, 130.2, 129.4, 129.2, 126.2, 123.5, 120.3, 115.6, 111.6, 54.9. LC-MS/MS (positive mode) *m*/*z*: 304 [M–OCH_3_+2K+3]^+^, 282 [M+2Na–H_2_O]^+^, 255 [M + H]^+^, 135 [methoxybenzyl]^+^.

#### 4.1.4 General Method for the Preparation of Gypsogenin (**2**)

Gypsogenin (**1**) was obtained from *Gypsophila arrostii* roots (Scheme 2) according to the previously described procedures.^14,15^ Structural characterization of gypsogenin and purity analyses were performed using TLC, NMR, and MS.

##### 4.1.4.1 3-Hydroxy-23-oxoolean-12-en-28-oic Acid gypsogenin (**2**)

mp 273–274 °C; LC-MS (ESI, negative mode) *m*/*z*: 469.20 [M–H]^−^.

#### 4.1.5 General Procedure for the Synthesis of Compound Acetyl-Gypso (**3**)

Gypsogenin (500 mg) was treated with acetic anhydride (2 mL) and DMAP in tetrahydrofuran (5 mL). The mixture was allowed to stir for 48 h at room temperature. After stirring, the mixture was extracted with CH_2_Cl_2_ (3 × 10 mL) and then the organic phase was evaporated to dryness. The residue was purified by column chromatography with hexane/ethyl acetate (8/2) to give compound **3** (Scheme 2).

##### 4.1.5.1 3-(Acetyloxy)-23-oxoolean-12-en-28-oic Acid (**3**)

Rf: 0.50 (Hexane-Ethyl acetate: 8:2); Yield: 98%; mp 165–166 °C; FT-IR KBr (cm^–1^): 3353, 2946, 1736, 1694, 1463, 1370, 1238, 1030, 1009, 736, 688, 645, 603, 509,485;

^1^H NMR (400 MHz, C_5_D_5_N) δ 9.79 (s, 1H), 5.76 (br s, 1H), 5.51 (dd, 1H), 3.59 (dd, 1H), 2.23 (s, 3H), 1.58 (s, 3H), 1.47 (s, 3H), 1.32 (s, 3H), 1.27 (s, 3H), 1.25 (s, 3H), 1.15 (s, 3H).

^13^C NMR (100 MHz, C_5_D_5_N) δ: 204.9, 180.4, 170.4, 145.2, 122.4, 73.8, 42.3, 34.6, 24.1, 23.1, 21.1, 17.6, 15.8, 10.1. LC-MS/MS (negative mode): *m*/*z*: 511.20 [M–H]^−^.

#### 4.1.6 General Procedure for the Synthesis of Compounds (**4a**–**4i**)

For this process, two natural hybrid derivatives were used as starting materials. Chalcone derivatives (**1a**–**1i**) (1 equiv) and Acetyl-Gypso (**3**) compound (2 equiv) were dissolved in CH_2_Cl_2_ (10 mL), after then added DCC (1.5 equiv) and DMAP under N_2_. The reaction mixture was allowed to stir at 0 °C for 72 h. After the mixture was stirred, the solvent was evaporated to dryness in the vacuum. The residue was purified by silica gel chromatography using Hexane:Ethyl Acetate (8:2) to give gypsogenin-chalcone hybrid compounds (**4a**–**4i**) (Scheme 5).

**Scheme 5:**
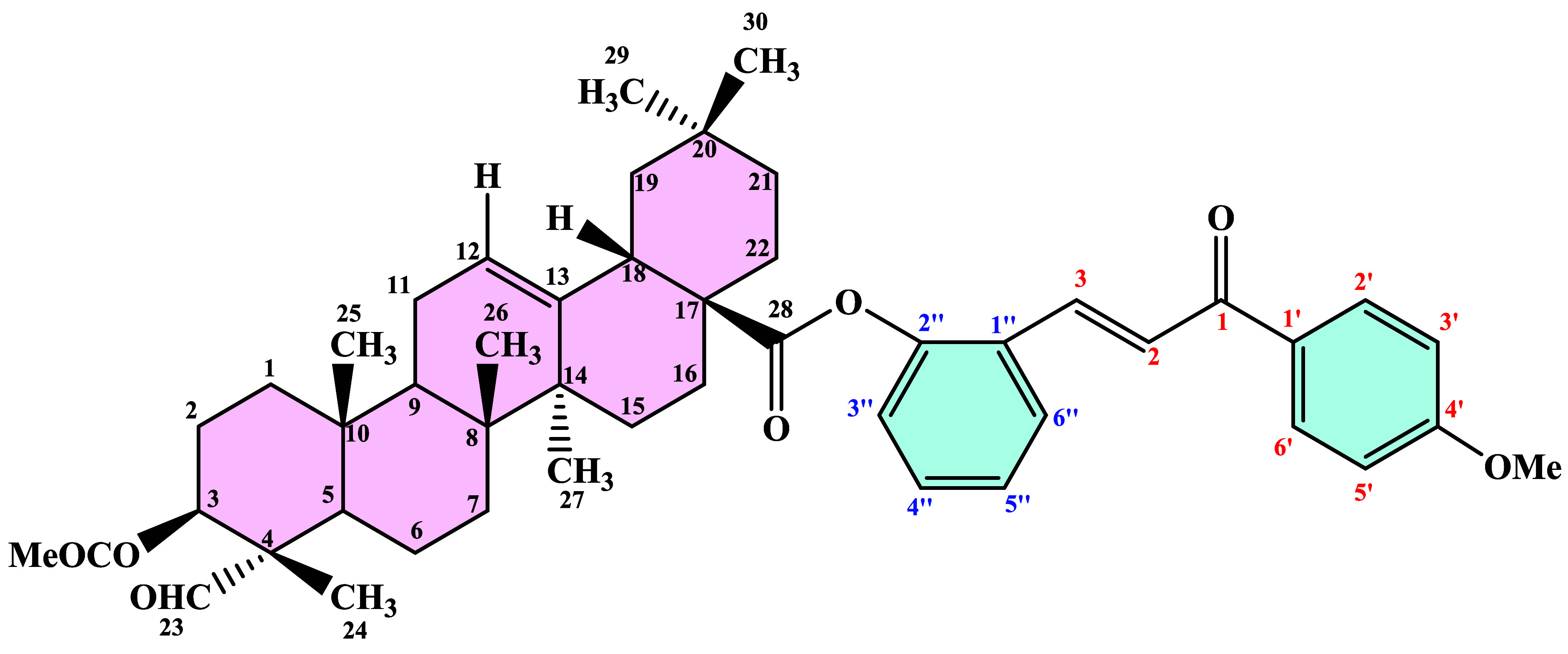
Numbering of atoms of compound (**4a**–**4i**) for NMR analysis

#### 4.1.7 Characterization of Compounds (**4a**–**4i**

##### 4.1.7.1 Acetyl Gypso-COO-Chalcone Hybrid Compound (**4a**)

Rf: 0.60 (Hexane-Ethyl Acetate: 7:3); Yield: 51%; mp 221–223.1 °C; FT-IR KBr (cm^–1^): 3048, 2921, 2850, 1733, 1625, 1573, 1467, 1341, 1279, 1146, 1096, 1059, 960, 841;

^1^H NMR (600 MHz, CDCl_3_) δ 9.20 (s, 1H), 7.92 (d, *J =* 8.6 Hz, 2H), 7.82 (d, *J =* 15.8 Hz, 1H), 7.64 (d, *J =* 8.2 Hz, 1H), 7.62 (d, *J =* 15.8 Hz, 1H), 7.13 (t, *J =* 7.8 Hz, 1H), 6.90 (d, *J =* 8.1 Hz, 1H), 6.77 (d, *J =* 8.6 Hz, 2H), 5.31 (br s, 1H), 4.89 (m, 1H), 3.83 (s, 3H), 2.95 (dd, 1H), 1.90 (s, 3H), 1.16 (s, 3H), 1.00 (s, 3H), 0.93 (s, 3H), 0.92 (s, 3H), 0.87 (s, 3H), 0.80 (s, 3H).

^13^C NMR (150 MHz, CDCl_3_) δ 204.9, 189.0, 175.9, 170.9, 163.4, 148.5, 143.4, 143.3, 132.9, 130.3, 130.1, 122.9, 122.4, 121.9, 119.9, 115.9, 113.7, 73.5, 55.4, 54.3, 41.2, 32.9, 25.6, 23.3, 20.8, 17.5, 15.5, 9.3. LC-MS/MS *m*/*z*: 749 [M]^+^.

##### 4.1.7.2 Acetyl Gypso-COO-Chalcone Hybrid Compound (**4b**)

Rf: 0.33 (Hexane- Ethyl Acetate: 8:2); Yield: 30%; mp 209–211 °C; FT-IR KBr (cm^–1^): 2925, 2851, 1741, 1599, 1450, 1359, 1341, 1279, 1090, 1059, 960, 946, 841;

^1^H NMR (600 MHz, CDCl_3_) δ 9.60 (s, H-23), 8.37 (d, *J =* 8.5 Hz, 2H), 8.23 (d, *J =* 15.7 Hz, 1H), 7.92 (d, *J =* 15.7 Hz, 1H), 7.57 (t, 1H), 7.16 (d, *J =* 8.5 Hz, 2H), 7.07 (s, 1H), 6.92 (s, 1H), 5.53 (br s), 5.31 (1H), 3.81 (s, 3H), 3.26 (dd, 1H), 2.01 (s, 3H), 1.35 (3H), 1.29 (3H), 1.05 (3H), 1.02 (3H), 0.95 (3H), 0.92 (3H).

^13^C NMR (150 MHz, CDCl_3_) δ: 205.0, 188.3, 176.6, 170.5, 164.3, 152.7, 144.1, 144.1, 143.1, 137.8, 130.7, 130.7, 129.9, 123.3, 122.3, 114.8, 114.8, 73.8, 55.8, 55.0, 42.6, 34.3, 28.5, 23.9, 21.1, 18.0, 15.9, 10.2. LC-MS/MS *m*/*z*: 749 [M]^+^.

##### 4.1.7.3 Acetyl Gypso-COO-Chalcone Hybrid Compound (**4c**)

Rf: 0.39 (Hexane-Ethyl Acetate: 8:2); Yield: 25%; mp 217.9–219 °C; FT-IR KBr (cm^–1^): 2927, 2849, 1731, 1624, 1571, 1443, 1309, 1240, 1162, 1087, 1026, 892, 801;

^1^H NMR (600 MHz, CDCl_3_) δ 9.41 (s, 1H), 8.21 (d, *J =* 8.4 Hz, 2H), 8.04 (d, *J =* 15.6 Hz, 1H), 7.76 (d, *J =* 15.6 Hz, 1H), 7.68 (d, *J =* 8.2 Hz, 2H), 7.12 (d, J *J =* 8.4 Hz, 2H), 6.93 (d, *J =* 8.2 Hz, 2H), 5.32 (br s, 1H), 4.09 (dd, 1H), 3.60 (s, 3H), 3.08 (dd, 1H), 1.81 (s, 3H), 1.15 (s, 3H), 1.09 (s, 3H), 0.86 (s, 3H), 0.85 (s, 3H), 0.82 (s, 3H), 0.73 (s, 3H).

^13^C NMR (150 MHz, CDCl_3_) δ 205.0, 188.4, 176.4, 170.5, 164.3, 160.3, 144.0, 143.2, 131.9, 131.9, 130.6, 124.2, 123.8, 123.5, 123.3, 114.8, 73.8, 55.1, 55.1, 40.5, 31.2, 26.4, 23.9, 21.2, 17.9, 15.9, 10.2. LC-MS/MS *m*/*z*: 749 [M]^+^.

##### 4.1.7.4 Acetyl Gypso-COO-Chalcone Hybrid Compound (**4d**)

Rf: 0.52 (Hexane-Ethyl Acetate: 8:2); Yield: 15%; mp 198–200 °C; FT-IR KBr (cm^–1^): 3050, 2923, 2850, 2340, 1744, 1699, 1652, 1553, 1457, 1380, 1336, 1308, 1237, 1181, 1002, 902, 822;

^1^H NMR (600 MHz, CDCl_3_) δ 9.28 (s, 1H), 8.20 (d, *J =* 15.5 Hz, 1H), 7.45 (d, *J =* 15.5 Hz, 1H), 7.42 (d, *J =* 8.1 Hz, 1H), 7.32 (t, *J =* 7.8 Hz, 1H), 7.08 (d, *J =* 8.1 Hz, 1H), 7.03 (d, *J =* 8.0 Hz, 1H), 7.02 (d, *J =* 8.0 Hz, 1H), 5.37 (br s, 1H), 4.97 (dd, 1H), 3.80 (s, 3H), 2.99 (dd, 1H), 1.96 (s, 3H), 1.22 (s, 3H), 1.08 (s, 3H), 0.98 (s, 3H), 0.93 (s, 3H), 0.88 (s, 3H), 0.86 (s, 3H).

^13^C NMR (150 MHz, CDCl_3_) δ 204.9, 190.6, 176.2, 170.7, 159.6, 143.7, 142.7, 138.7, 129.6, 129.5, 129.5, 126.9, 122.7, 122.3, 120.1, 119.3, 119.3, 115.2, 112.9, 73.4, 55.6, 54.2, 39.9, 32.7, 25.5, 23.3, 20.6, 17.2, 15.3, 9.1. LC-MS/MS *m*/*z*: 749 [M]^+^.

##### 4.1.7.5 Acetyl Gypso-COO-Chalcone Hybrid Compound (**4e**)

Rf: 0.48 (Hexane-Ethyl Acetate: 8:2); Yield: 51%; mp 152–153 °C; FT-IR KBr (cm^–1^): 3045, 2924, 2891, 2683, 2361, 1785, 1718, 1646, 1560, 1354, 1335, 1261, 1175, 1078, 1048, 961, 877, 744;

^1^H NMR (600 MHz, CDCl_3_) δ 9.28 (s, 1H), 7.75 (d, *J =* 15.6 Hz, 1H), 7.59 (d, 1H), 7.51 (d, *J =* 15.6 Hz, 1H), 7.46 (s, 1H), 7.41 (t, 1H), 7.29 (t, 1H), 7.13 (d, 1H), 7.05 (d, 1H), 5.37 (br s, 1H), 4.97 (dd, 1H), 3.88 (s, 3H), 2.96 (dd, 1H), 1.96 (s, 3H), 1.18 (s, 3H), 1.07 (s, 3H), 0.97 (s, 3H), 0.95 (s, 3H), 0.93 (s, 3H), 0.88 (s, 3H).

^13^C NMR (150 MHz, CDCl_3_) δ 204.5, 190.1, 176.2, 170.1, 159.9, 159.8, 143.9, 143.4, 139.4, 136.4, 129.8, 129.5, 122.0, 122.0, 121.1, 119.9, 119.3, 112.9, 112.7, 73.5, 55.7, 54.2, 41.0, 32.9, 25.9, 23.4, 20.7, 17.2, 15.4, 9.3. LC-MS/MS *m*/*z*: 749 [M]^+^.

##### 4.1.7.6 Acetyl Gypso-COO-Chalcone Hybrid Compound (**4f**)

Rf: 0.40 (Hexane-Ethyl Acetate: 8:2); Yield: 42%; mp 101.2–102.3 °C; FT-IR KBr (cm^–1^): 3359, 3043, 2928, 2854, 1733, 1698, 1663, 1505, 1451, 1369, 1239, 1164, 1150, 1028, 894;

^1^H NMR (600 MHz, CDCl_3_) δ 9.28 (s, 1H), 7.78 (d, *J =* 15.6 Hz, 1H), 7.53 (d, 1H), 7.50 (s, 1H), 7.46 (s, 1H), 7.41 (t, *J =* 7.8 Hz, 1H), 7.32 (d, *J =* 15.6 Hz, 1H), 7.08 (d, *J =* 8.0 Hz, 1H), 6.91 (d, *J =* 8.0 Hz, 1H), 5.36 (br s, 1H), 5.00 (dd, *J =* 11.5, 4.2 Hz, 1H), 3.89 (s, 3H), 2.96 (dd, 1H), 1.96 (s, 3H), 1.12 (s, 3H), 1.06 (s, 3H), 0.98 (s, 3H), 0.95 (s, 3H), 0.90 (s, 3H), 0.81 (s, 3H).

^13^C NMR (150 MHz, CDCl_3_) δ: 204.3, 189.2, 180.4, 170.1, 161.5, 161.5, 143.9, 143.4, 138.7, 132.5, 129.5, 123.1, 120.1, 119.4, 112.7, 74.1, 55.5, 42.7, 33.0, 25.8, 23.5, 20.9, 17.2, 15.6, 9.5. LC-MS/MS *m*/*z*: 749.91 [M+1]^+^.

##### 4.1.7.7 Acetyl Gypso-COO-Chalcone Hybrid Compound (**4g**)

Rf: 0.38 (Hexane- Ethyl Acetate: 8:2); Yield: 20%; mp 181.6 – 182.4 °C; FT-IR KBr (cm^–1^): 3039, 2928, 2118, 1734, 1604, 1554, 1483, 1450, 1240, 1150, 1027, 891;

^1^H NMR (600 MHz, CDCl_3_) δ 9.28 (s, 1H), 7.90 (d, *J =* 15.5 Hz, 1H), 7.61 (d, 1H), 7.54 (d, 1H), 7.46 (d, 1H), 7.03 (d, *J =* 8.0 Hz, 1H), 7.00 (d, 1H), 6.93 (d, 1H), 6.92 (d, 1H), 6.90 (d, *J =* 15.5 Hz, 1H), 6.84 (d, 1H), 5.35 (br s, 1H), 4.98 (dd, 1H), 3.84 (s, 3H), 2.98 (dd, 1H), 1.96 (s, 3H), 1.20 (s, 3H), 1.08 (s, 3H), 0.98 (s, 3H), 0.94 (s, 3H), 0.88 (s, 3H), 0.84 (s, 3H).

^13^C NMR (150 MHz, CDCl_3_) δ: 204.4, 193.4, 178.2, 170.2, 158.6, 158.1, 143.5, 143.4, 136.4, 131.1, 129.0, 129.0, 128.9, 125.4, 122.6, 121.4, 121.3, 120.1, 116.3, 111.8, 73.3, 55.5, 54.3, 41.5, 32.9, 25.6, 23.6, 20.9, 15.6, 14.1, 9.6. LC-MS/MS *m*/*z*: 743 [M-OCH_3_+Na+2] ^+^.

##### 4.1.7.8 Acetyl Gypso-COO-Chalcone Hybrid Compound (**4h**)

Rf: 0.38 (Hexane- Ethyl Acetate: 8:2); Yield: 68%; mp 211.6 – 213.2 °C; FT-IR KBr (cm^–1^): 3043, 2929, 1733, 1629, 1563, 1450, 1370, 1318, 1242, 1149, 1028, 891;

^1^H NMR (600 MHz, CDCl_3_) δ 9.26 (s, 1H), 7.69 (d, *J =* 15.4 Hz, 1H), 7.53 (d, *J =* 15.4 Hz, 1H), 7.02 (t, *J =* 7.6 Hz, 1H), 6.98 (d, *J =* 8.1 Hz, 1H), 5.35 (br s, 1H), 4.98 (dd, 1H), 3.88 (s, 3H), 2.97 (dd, 1H), 1.95 (s, 3H), 1.20 (s, 3H), 1.07 (s, 3H), 0.96 (s, 3H), 0.94 (s, 3H), 0.91 (s, 3H), 0.84 (s, 3H).

^13^C NMR (150 MHz, CDCl_3_) δ: 204.5, 192.8, 176.1, 170.3, 158.1, 156.8, 143.3, 142.1, 136.7, 129.2, 125.6, 122.4, 111.7, 73.4, 55.8, 54.3, 41.5, 33.0, 25.6, 23.6, 20.4, 17.4, 15.6, 9.5. LC-MS/MS *m*/*z*: 716 [M-(CH_3_CO+OMe) + K+2]^+^.

##### 4.1.7.9 Acetyl Gypso-COO-Chalcone Hybrid Compound (**4i**)

Rf: 0.50 (Hexane- Ethyl Acetate: 8:2); Yield: 32%; mp 202 – 204 °C; FT-IR KBr (cm^–1^): 2926, 2883, 1967, 1719, 1603, 1466, 1341, 1279, 1095, 960, 841;

^1^H NMR (600 MHz, CDCl_3_) δ 9.28 (s, 1H), 7.70 (m, 1H), 7.68 (d, 1H), 7.62 (d, *J =* 8.4 Hz, 2H), 7.58 (d, *J =* 15.3 Hz, 1H), 7.05 (d, *J =* 15.3 Hz, 1H), 6.98 (d, *J =* 8.2 Hz, 1H), 6.92 (d, 1H), 6.75 (d, J = 8.4 Hz, 2H), 5.34 (br s, 1H), 4.98 (m, 1H), 3.84 (s, 3H), 2.97 (dd, 1H), 1.96 (s, 3H), 1.20 (s, 3H), 1.09 (s, 3H), 0.99 (s, 3H), 0.96 (s, 3H), 0.94 (s, 3H), 0.85 (s, 3H).

^13^C NMR (150 MHz, CDCl_3_) δ 205.2, 196.2, 176.2, 170.4, 161.6, 158.2, 143.4, 142.3, 133.7, 130.7, 129.4, 122.2, 120.9, 111.6, 73.3, 55.6, 54.2, 41.5, 32.8, 25.7, 23.4, 20.7, 17.2, 15.4, 9.3. LC-MS/MS *m*/*z*: 749 [M]^+^.

### 4.2 Biological Activity

#### 4.2.1 Antimicrobial Activity

In microdilution method applied for antimicrobial activity, bacterial strains were cultured on Mueller–Hinton agar (MHA) plaque media and yeast broth Sabouraud dekstroz agar (SDA) plaque media and incubated at 37 °C for 18–20 h. Suspensions were prepared from fresh cultures with 0.9% NaCl solution (saline), and the suspensions were adjusted to 0.5 (1–2 × 108 CFU/mL) with the McFarland device (Den-1, Biosan). The suspensions were diluted 1/100. 50 μL of bacteria were distributed to the wells of sterile 96-well microplates, with Mueller-Hinton II broth (cation-added) (MHIIB) for bacterial origins (*Staphylococcus aureus* ATCC 29213, *Enterococcus faecalis* ATCC 29212, *Escherichia coli* ATCC 25922, *Pseudomonas aeruginosa* ATCC 27853, *Bacillus subtilis* RSKK 02021) and RPMI-1640 (buffered with MOPS) for yeast origins (*Candida albicans* ATCC 90028). First batches were added to 50 μL of active substance solutions (DMSO/distilled water, 1/1), and serial dilutions were prepared by transferring to the side wells. Fifty microliter of bacterial and fungal suspensions was added to the wells. The lowest concentrations without growth after 16–24 h of incubation at 37 °C were accepted as MIC. Feeding and reproductive controls were made in every microplate. The control group used ciprofloxacin and fluconazole as antimicrobial agents. MIC interpretation limits and control limits were evaluated according to the CLSI and EUCAST criteria.

#### 4.2.2 In Vitro Cytotoxicity

##### 4.2.2.1 Cancer Cell Lines and Cell Culture

The anticancer activities of the synthesized compounds were evaluated using PANC-I (human pancreatic cancer), MDA-MB-231 (human breast cancer), HeLa (human cervical cancer), A-549 (human lung cancer), and SHSY-5Y (human neuroblastoma cancer) cell lines and HEK293 as the normal cell line. The cells were incubated in DMEM Ham’s F12 culture medium at 37 °C in a CO_2_-regulated incubator. Cells were passaged twice weekly, and actively proliferating cells in the logarithmic growth phase were utilized for the experiments.

##### 4.2.2.2 Cytotoxicity Assay

The cytotoxicity of the compounds was screened against cancer cell lines (PANC-I, MDA-MB-231, HeLa, A-549, and SHSY-5Y) and a normal cell line (HEK293) using the MTT assay [3-(4,5-dimethyl-2-thiazolyl)-2,5-diphenyl-2H-tetrazolium bromide)]. The test is based on the conversion of the tetrazolium salt MTT to soluble formazan by mitochondrial reductase in viable cells. For this purpose, cell lines were seeded in 96-well culture plates at an initial concentration of 1 × 10^5^ cells/mL and incubated for 24 h in a humidified incubator at 37 °C with 5% CO_2_. Subsequently, different concentrations of the test compounds were added to the cultured cells, which were then incubated for 48 h. Doxorubicin was used as a positive control. After 48 h of incubation, MTT solution was added, and the plates were incubated for an additional 4 h at 37 °C. The resulting material was dissolved in DMSO (dimethyl sulfoxide), and the absorbance was measured at a wavelength of λ=570 nm (reference filter, 630 nm) using a UV–visible spectrophotometer (Molecular Devices, UK).

For comparative purposes, all bioactivity values were converted from μg/mL to micromolar units (μM) using the molecular weights of the corresponding compounds.

#### 4.2.3 Determination of IC50 and TSI Values

Cytotoxicity was expressed as a percentage relative to the control ± SD, where the control was considered 0% cytotoxic. The cytotoxicity data were fitted to a sigmoidal curve, and the IC_50_ value, defined as the concentration of the compound required to inhibit cell growth by 50% compared with the untreated control group, was calculated using a four-parameter logistic model. The IC_50_ value represents the concentration under experimental conditions that reduces cell growth by 50%. This value is the mean of at least three independent and statistically significant replicates. The IC_50_ value was determined by a 95% confidence interval. This analysis was performed by using GraphPad Prism (San Diego, CA). Tumor selectivity was assessed by calculating the Tumor Selectivity Index (TSI) as the ratio of IC_50_ values in normal HEK293 cells to those in cancer cell lines, using mean IC_50_ values derived from cytotoxicity assays.

### 4.3 Molecular Docking Studies

Molecular docking studies of compounds **1g**, **1h**, and doxorubicin with different targets were performed using the Glide module of Schrödinger Maestro 14.1 software.^27^ The binding parameters and interaction modes of compounds **1g** and **1h**, which exhibited pronounced activity in the tested cancer cell lines, were calculated by using molecular modeling approaches. This method involves several standard steps, including the preparation of ligands and protein targets, followed by the evaluation of ligand–protein interactions.^27^

Compounds **1g**, **1h,** and doxorubicin were optimized using the LigPrep wizard of Schrödinger Maestro 14.1 software.^27^ The next step is to determine the crystal structures of the targets with which the ligands interact in molecular docking. Given that anticancer activity may involve multiple molecular targets, six different targets should be selected for molecular docking analysis to better explain the experimental cytotoxicity results. When these targets are selected, crystal structures related to signaling pathways associated with cancer cell lines are considered. Signaling pathways are obtained using the “KEGG Pathway Database” (https://www.genome.jp/kegg/pathway.html). In this database, the most important pathways related to each cell line and in anticancer drugs are pharmacologically determined, and the crystal structures of the relevant target are selected from there. Since the cell lines A549, Hela, HEK293, SHSY5Y, MDA-MB231, and PANC1 types were examined, the targets were selected accordingly. Epidermal growth factor receptor is a receptor that is important in all anticancer research and attempts to prevent cell metastasis.^21^ Since breast cancer cell lines were the target of the study, the estrogen receptor was chosen.^24,30^ For pancreatic and lung cell lines, Akt-1, PI3K-alpha, tubulin, and VEGFR-2 were identified as targets.^22,25,26,28,31^ Subsequently, the crystal structure with the lowest resolution and structural elucidation via X-ray crystallography was selected from the protein data bank (https://www.rcsb.org/).^32–34^

The six crystal structures obtained (PDB IDs: 4HJO, 3MV5, 5XGJ, 3ERT, 1SA0, 4ASD) were prepared with the ProteinPrep wizard of Schrödinger Maestro 14.1.^27^ The active pocket region for the ligand to be placed in hefein was determined using the Glide Grid wizard. In the final stage, each determined target was interacted with all three compounds. The accuracy of the results was confirmed by performing redocking.

## Supplementary Material



## Notes

Experimental procedures; characterization data of compounds **1a**–**1i** and **4a**–**4i**; ^1^H and ^13^C NMR spectra; COSY and HMQC spectra; MS spectra; UV spectra (PDF)
