# Supplementary material for: Design, Synthesis, and Biological Evaluation of Methoxy-Substituted Chalcones and Gypsogenin-Based Hybrids as Anticancer and Antimicrobial Agents
Source: ACS Omega. 2026 Apr 21;11(17):25537–51. doi: 10.1021/acsomega.5c13679 (PMC13150756; doi:10.1021/acsomega.5c13679)
Supplement: Supplementary file 1 [file ao5c13679_si_001.pdf]

SUPPLEMANTARY DATA

Design, Synthesis, and Biological Evaluation of Methoxy-Substituted Chalcones and Gypsogenin-Based Hybrids as Anticancer and Antimicrobial Agents

Nafia Gökçe Ulusoy[a], Safiye Emirdağ[a], Nuran Kahriman[b], Fatma Demir[b], Vildan Serdaroğlu[c], Nurettin Yaylı[d], Burçin Türkmenoğlu[e]

[a] Faculty of Science, Department of Chemistry, Ege University, Izmir, 35040, Türkiye

[b] Faculty of Science, Department of Chemistry, Karadeniz Technical University, Trabzon, 61080, Türkiye

[c] Central Kanuni Campus Scientific Research Center Building, Karadeniz Technical University, Trabzon, 61080, Türkiye

[d] Faculty of Pharmacy, Karadeniz Technical University, Trabzon, 61080, Türkiye

[e] Faculty of Pharmacy, Department of Analytical Chemistry, Erzincan Binali Yıldırım University, 24002 Erzincan, Türkiye

CHALCONES (1a-1i)

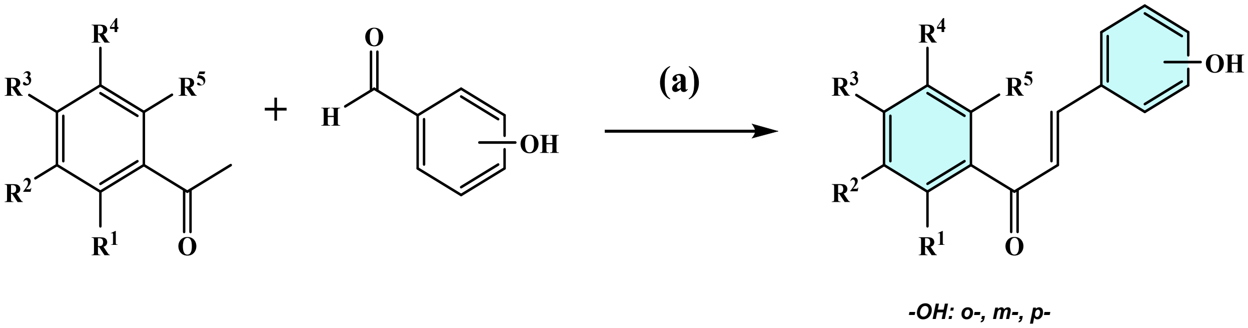

Chalcones (**1a-1i**)

Chalcones	R1	R2	R3	R4	R5
**1a – c**	-H	-H	**-OCH$_3$**	-H	-H
**1d – 1f**	-H	**-OCH$_3$**	-H	-H	-H
**1g – 1i**	**-OCH$_3$**	-H	-H	-H	-H

Reagents and conditions: (a) NaOH, H_2O/EtOH, RT (room temperature)

1a: *(2E)-3-(2-Hydroxyphenyl)-1-(4-methoxyphenyl) prop-2-en-1-one*

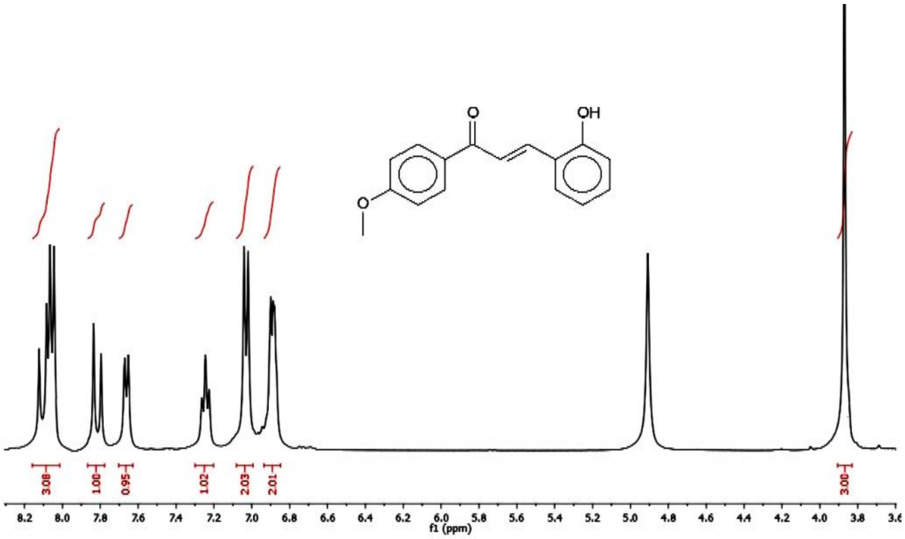

Figure S1. ^{1}H-NMR spectrum of compound **1a** (400 MHz, $CDCl_3/CD_3OD$, ppm)

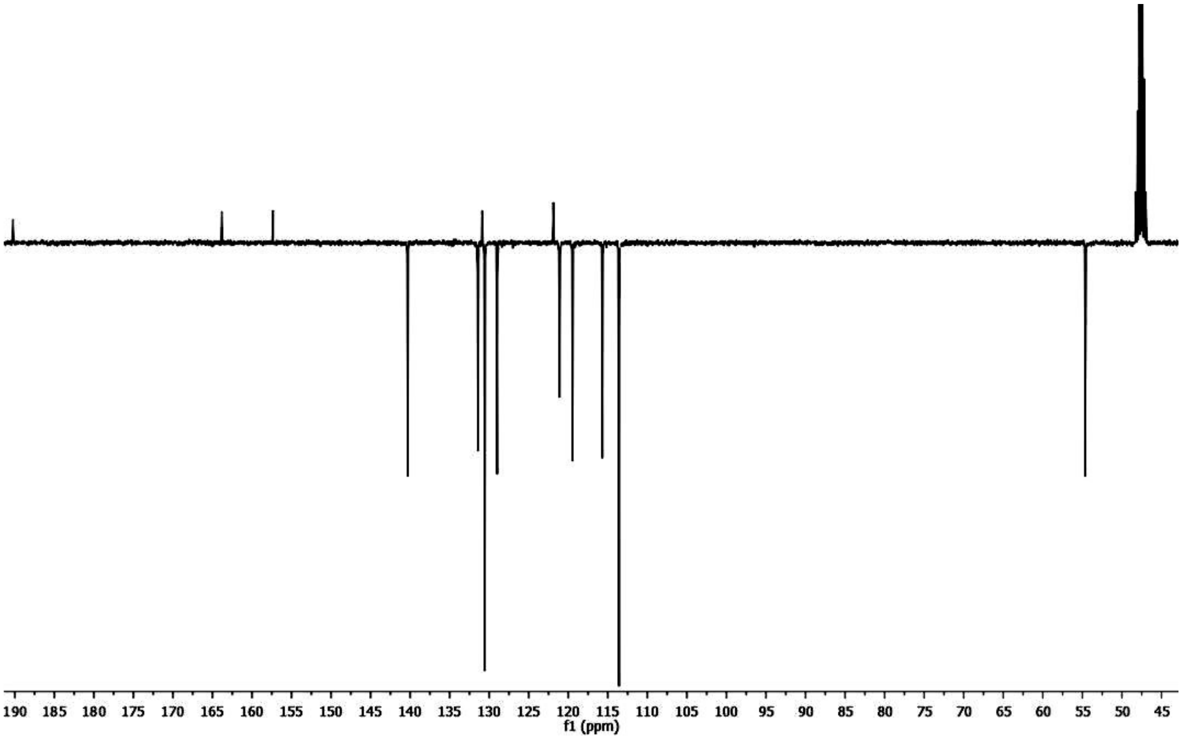

Figure S2. ^{13}C-APT NMR spectrum of compound **1a** (100 MHz, CD_3OD, ppm)

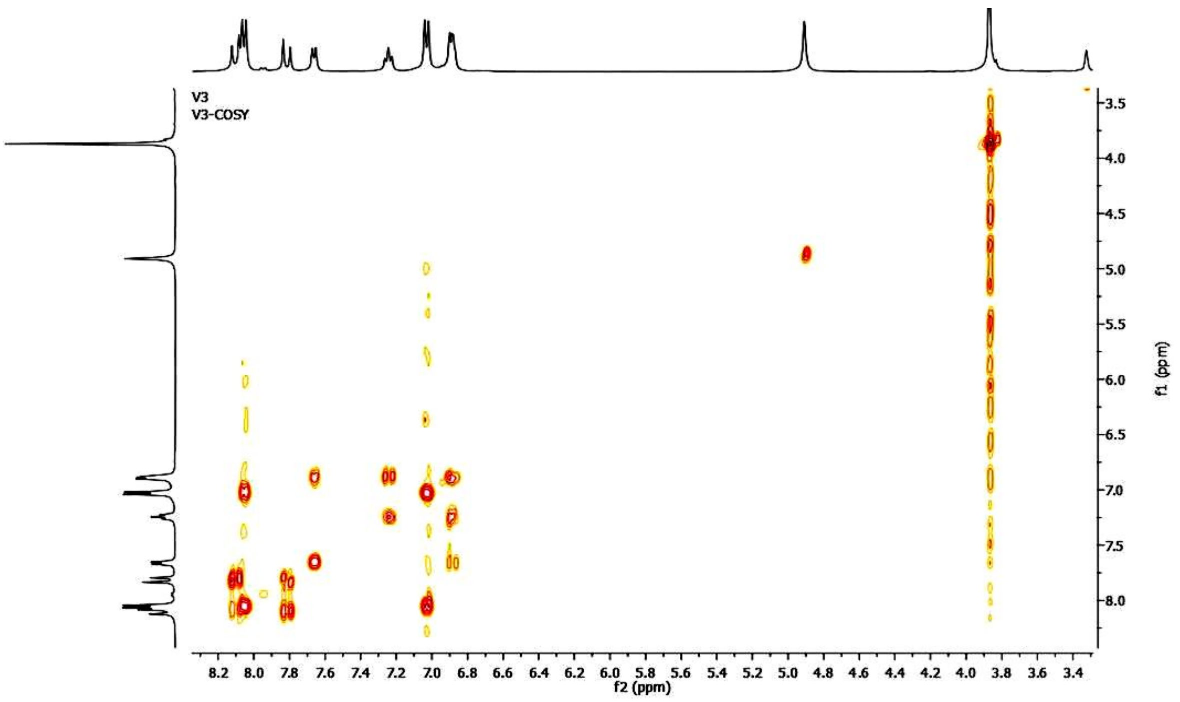

Figure S3. ^{1}H-^{1}H COSY NMR spectrum of compound **1a** (400 MHz, $CDCl_3/CD_3OD$, ppm)

1b: *(2E)-3-(3-Hydroxyphenyl)-1-(4-methoxyphenyl) prop-2-en-1-one*

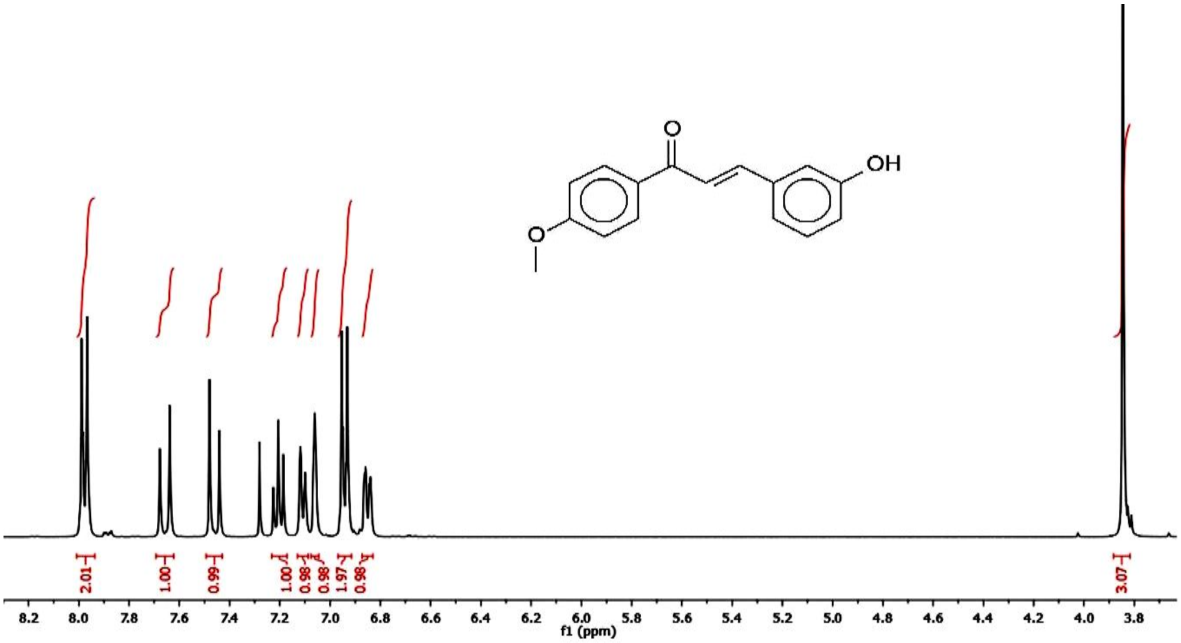

^{1}H-

Figure S4. ^{1}H-NMR spectrum of compound **1b** (400 MHz, $CDCl_3/CD_3OD$, ppm)

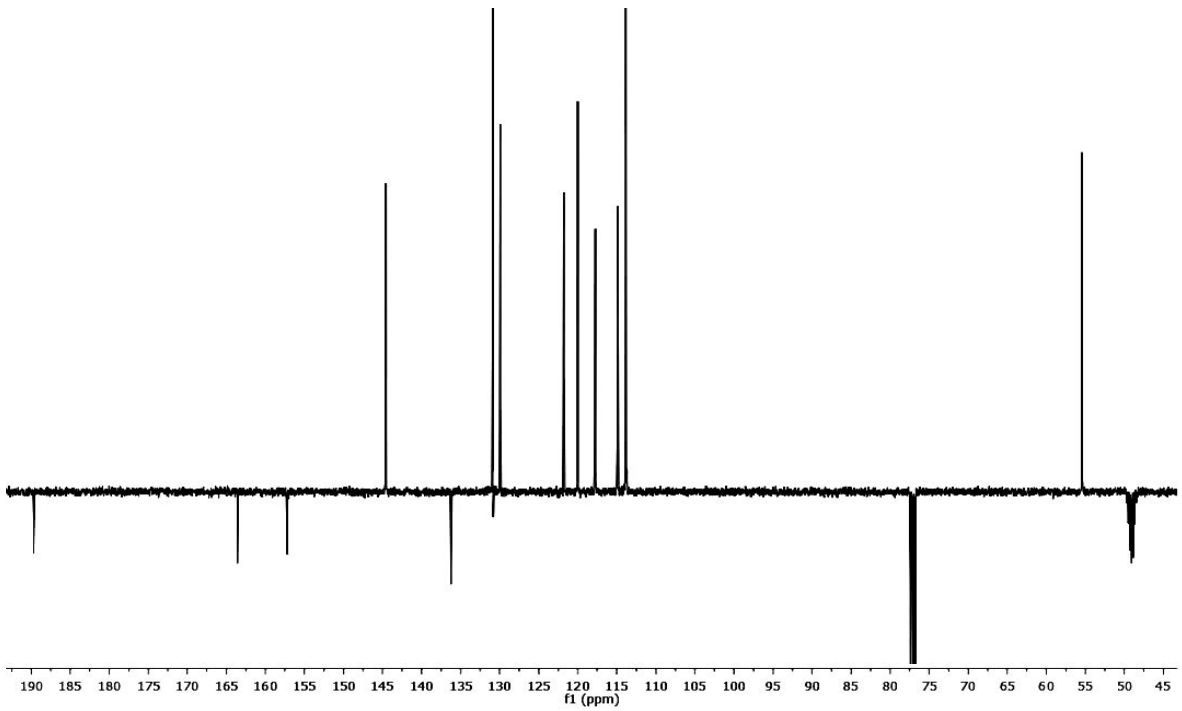

Figure S5. ^{13}C-APT NMR spectrum of compound **1b** (100 MHz, CD_3OD, ppm)

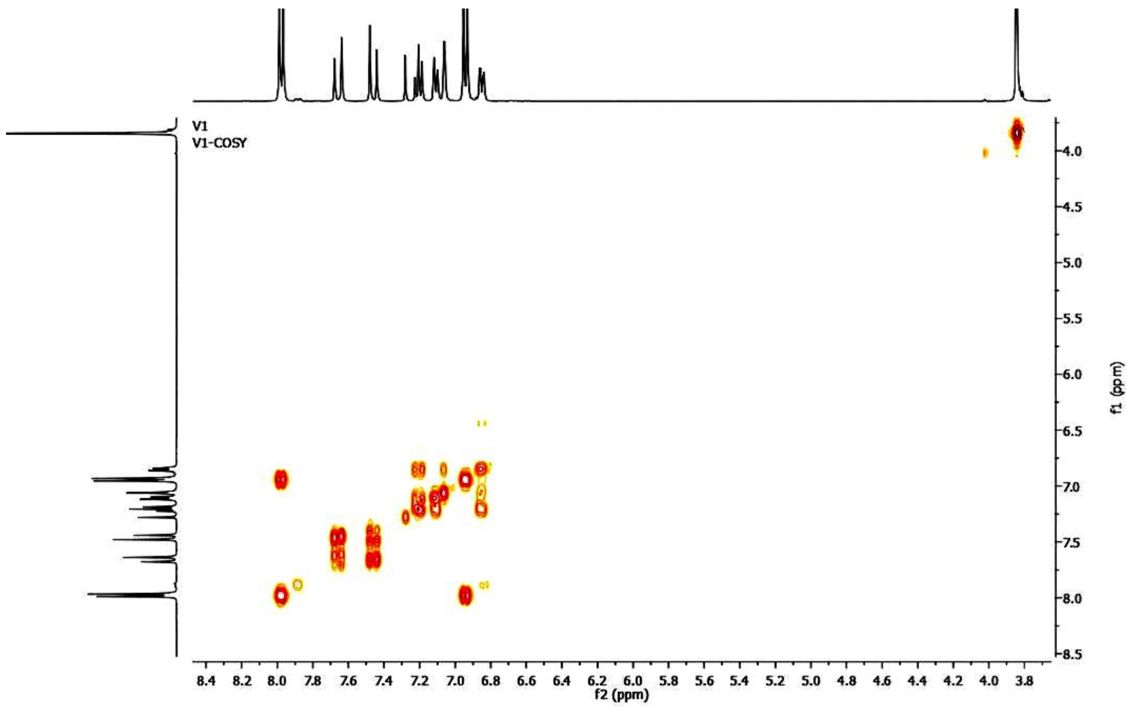

Figure S6. ^{1}H-^{1}H COSY NMR spectrum of compound **1b** (400 MHz, $CDCl_3/CD_3OD$, ppm)

1c: *(2E)-3-(4-Hydroxyphenyl)-1-(4-methoxyphenyl) prop-2-en-1-one*

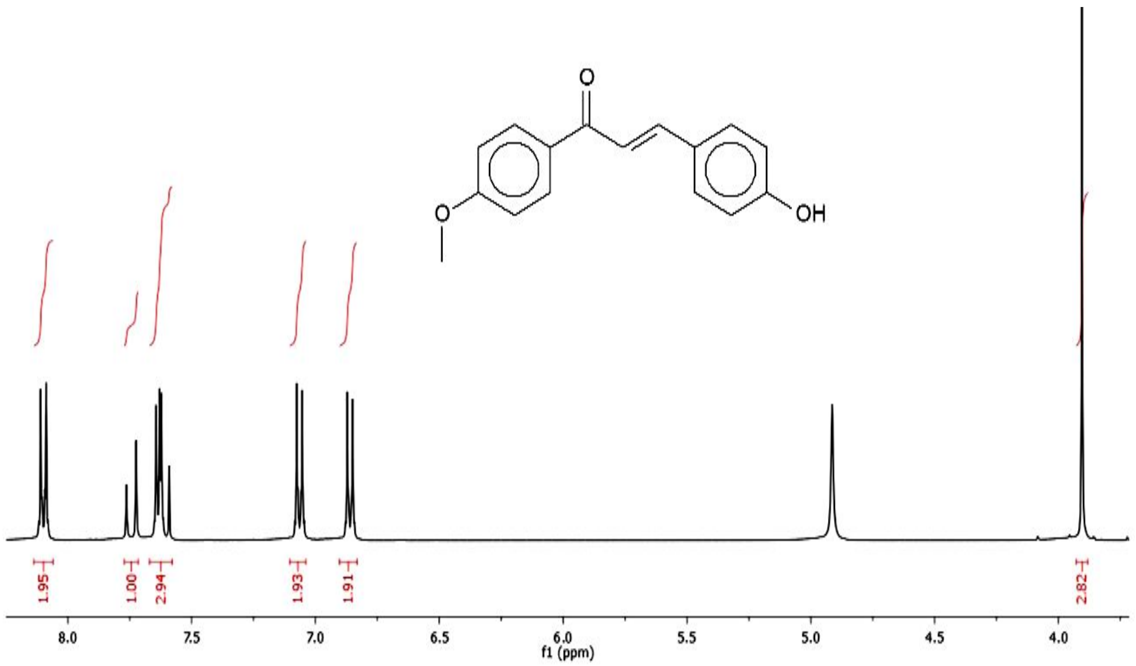

Figure S7. ^{1}H-NMR spectrum of compound **1c** (400 MHz, $CDCl_3/CD_3OD$, ppm)

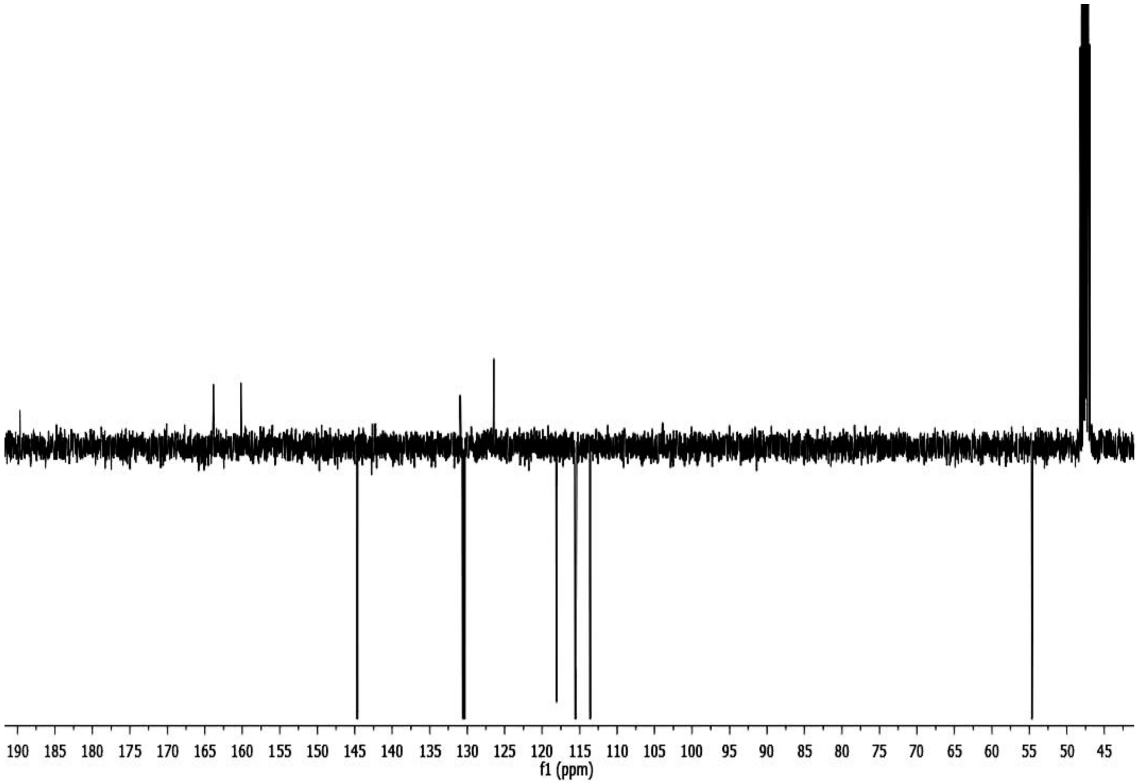

Figure S8. ^{13}C-APT NMR spectrum of compound **1c** (100 MHz, CD_3OD, ppm)

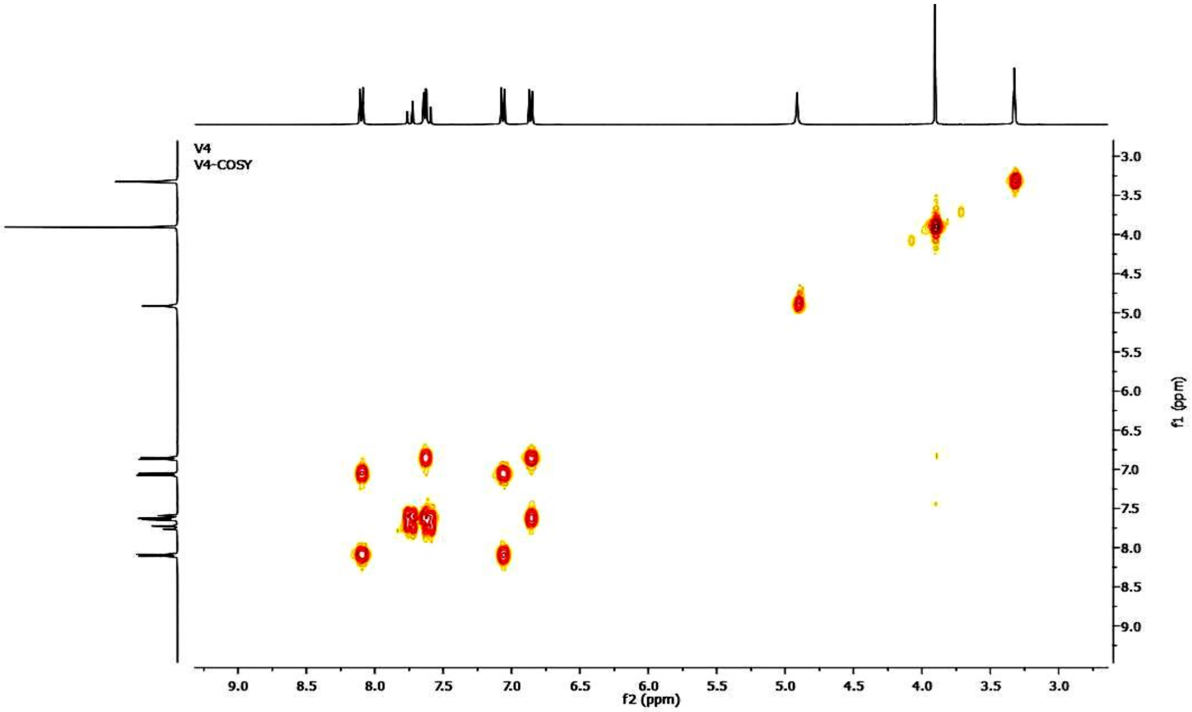

Figure S9. ^{1}H-^{1}H COSY NMR spectrum of compound **1c** (400 MHz, $CDCl_3/CD_3OD$, ppm)

1d: *(2E)-3-(2-Hydroxyphenyl)-1-(3-methoxyphenyl) prop-2-en-1-one*

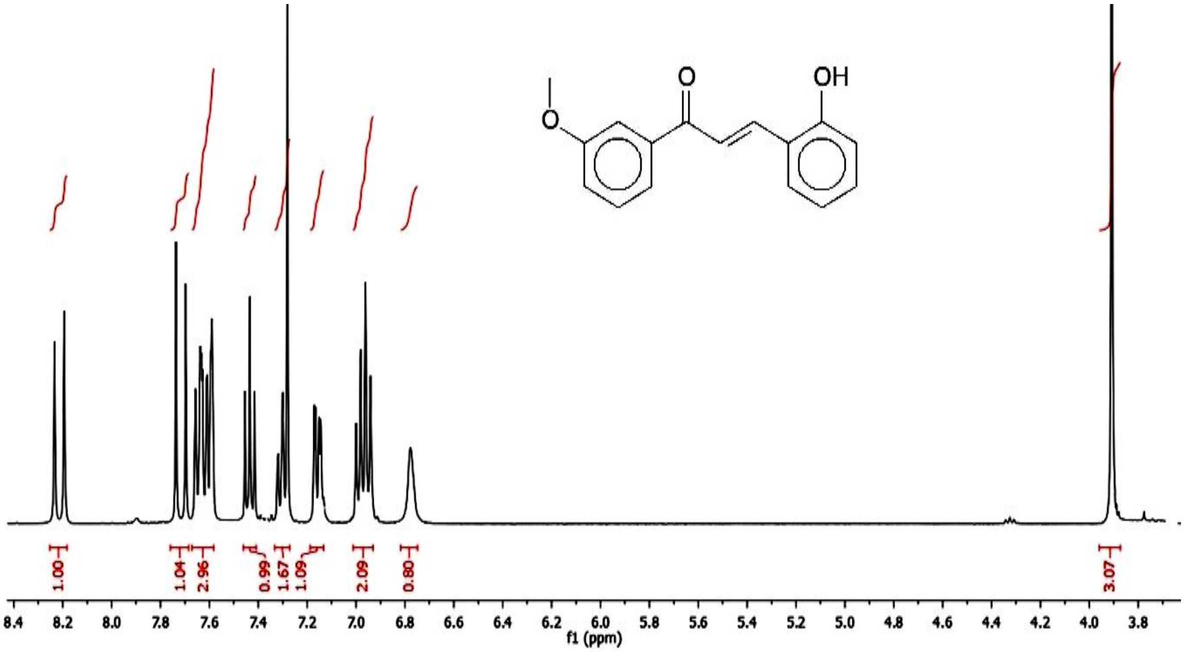

Figure S10. ^{1}H-NMR spectrum of compound **1d** (400 MHz, $CDCl_3/CD_3OD$, ppm)

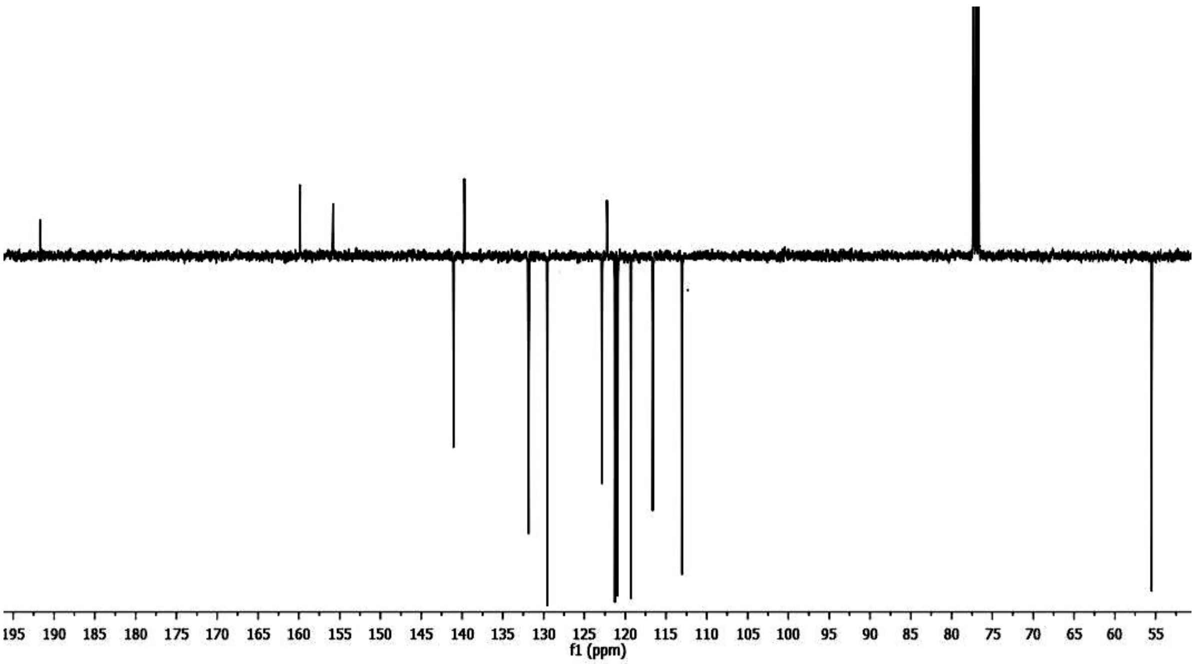

Figure S11. ^{13}C-APT NMR spectrum of compound **1d** (100 MHz, CD_3OD, ppm)

1e: *(2E)-3-(3-Hydroxyphenyl)-1-(3-methoxyphenyl) prop-2-en-1-one*

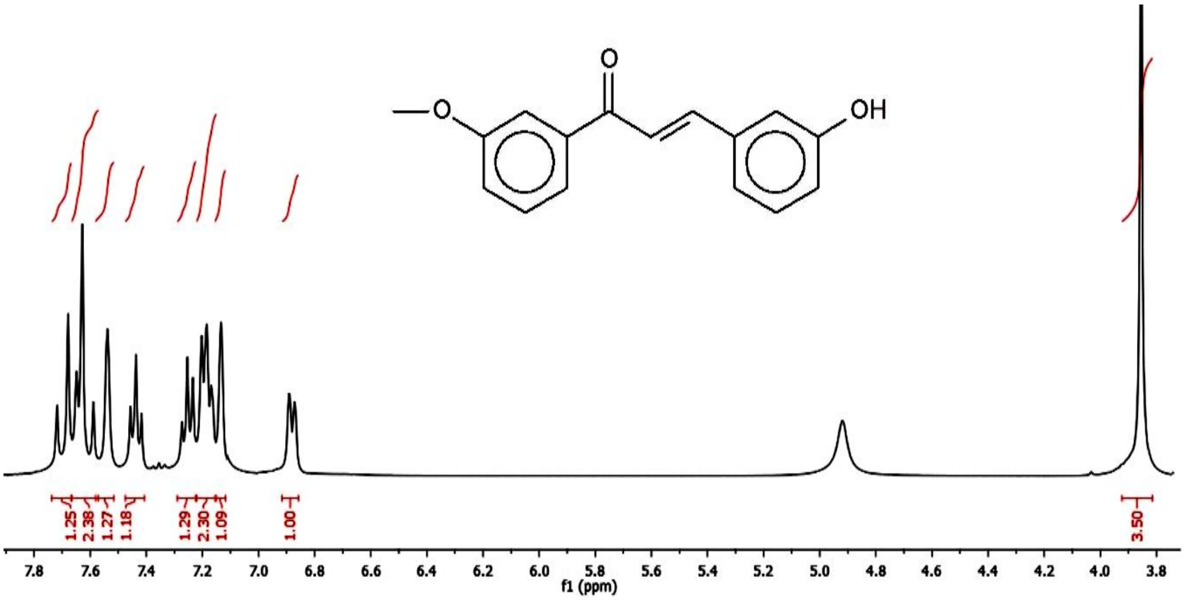

Figure S12. ^{1}H-NMR spectrum of compound **1e** (400 MHz, $CDCl_3/CD_3OD$, ppm)

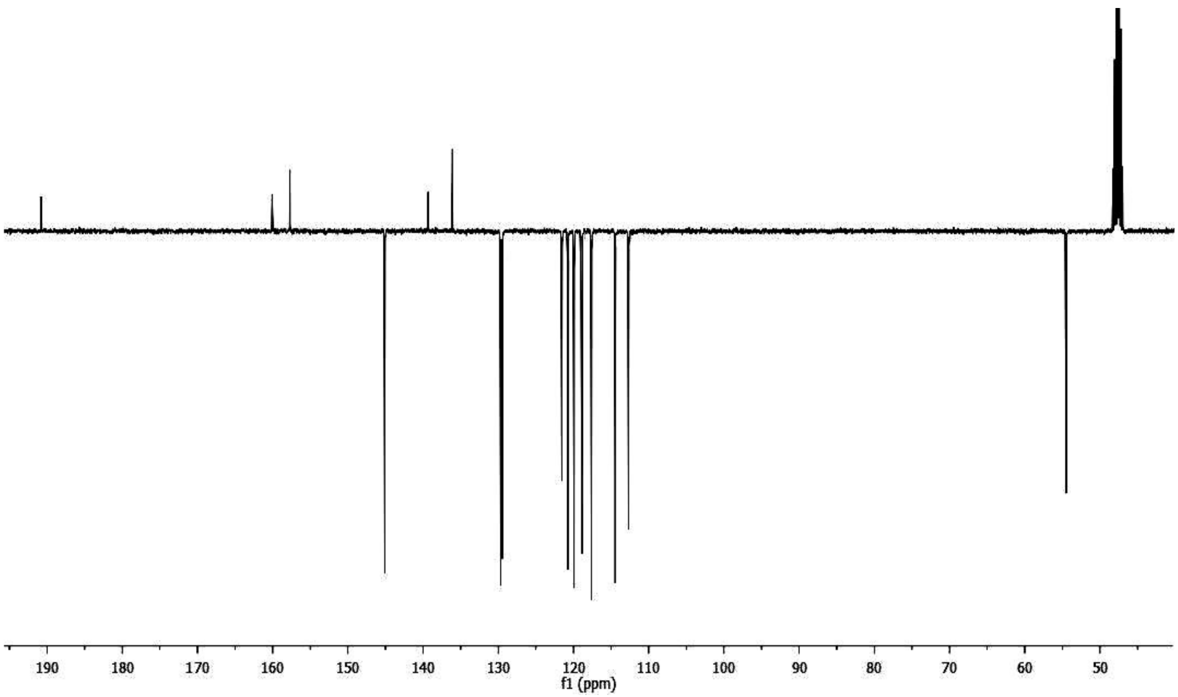

Figure S13. ^{13}C-APT NMR spectrum of compound **1e** (100 MHz, CD_3OD, ppm

1f: *(2E)-3-(4-Hydroxyphenyl)-1-(3-methoxyphenyl) prop-2-en-1-one*

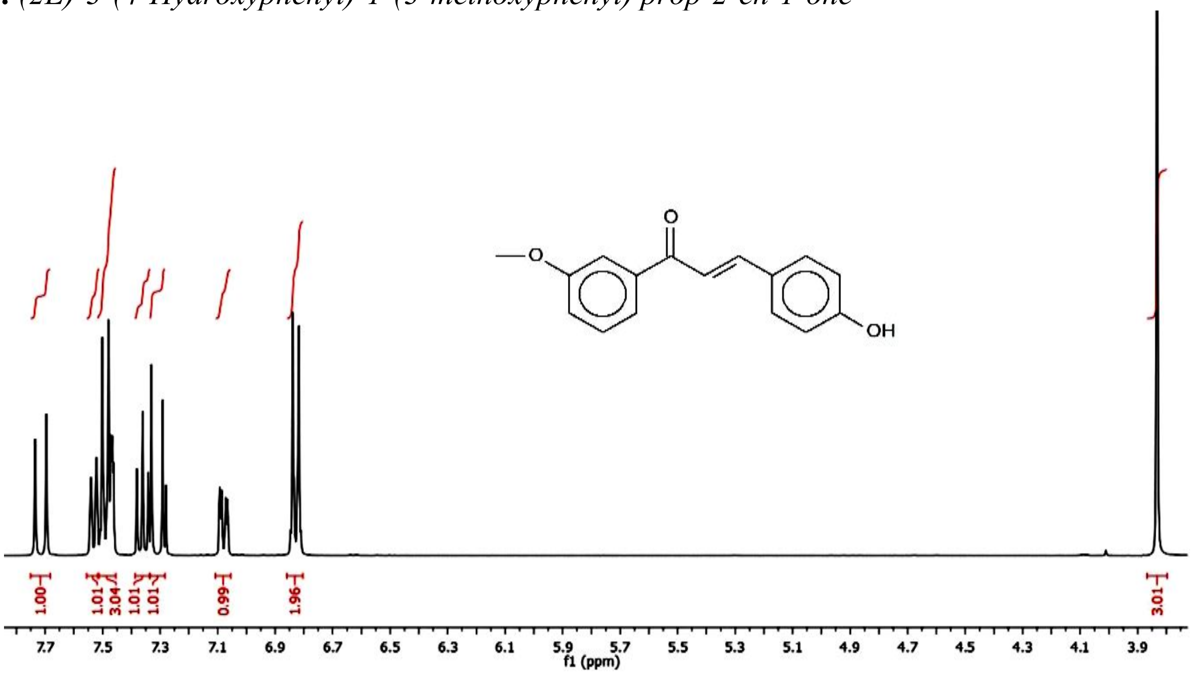

Figure S14. ^{1}H-NMR spectrum of compound **1f** (400 MHz, $CDCl_3/CD_3OD$, ppm)

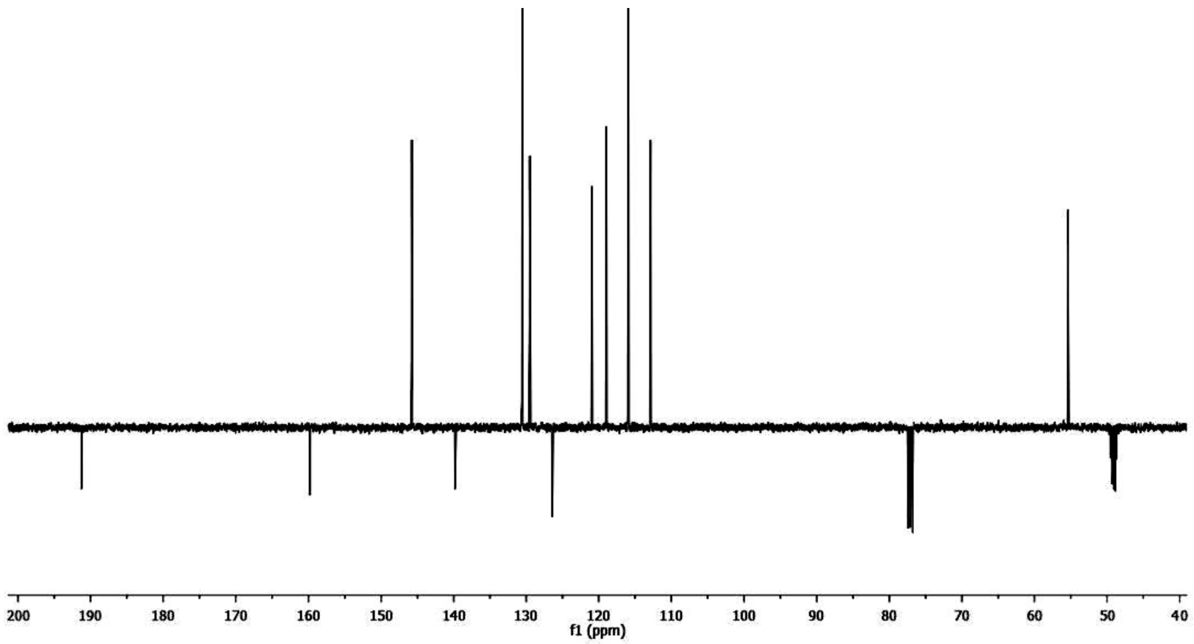

Figure S15. ^{13}C-APT NMR spectrum of compound **1f** (100 MHz, CD_3OD, ppm)

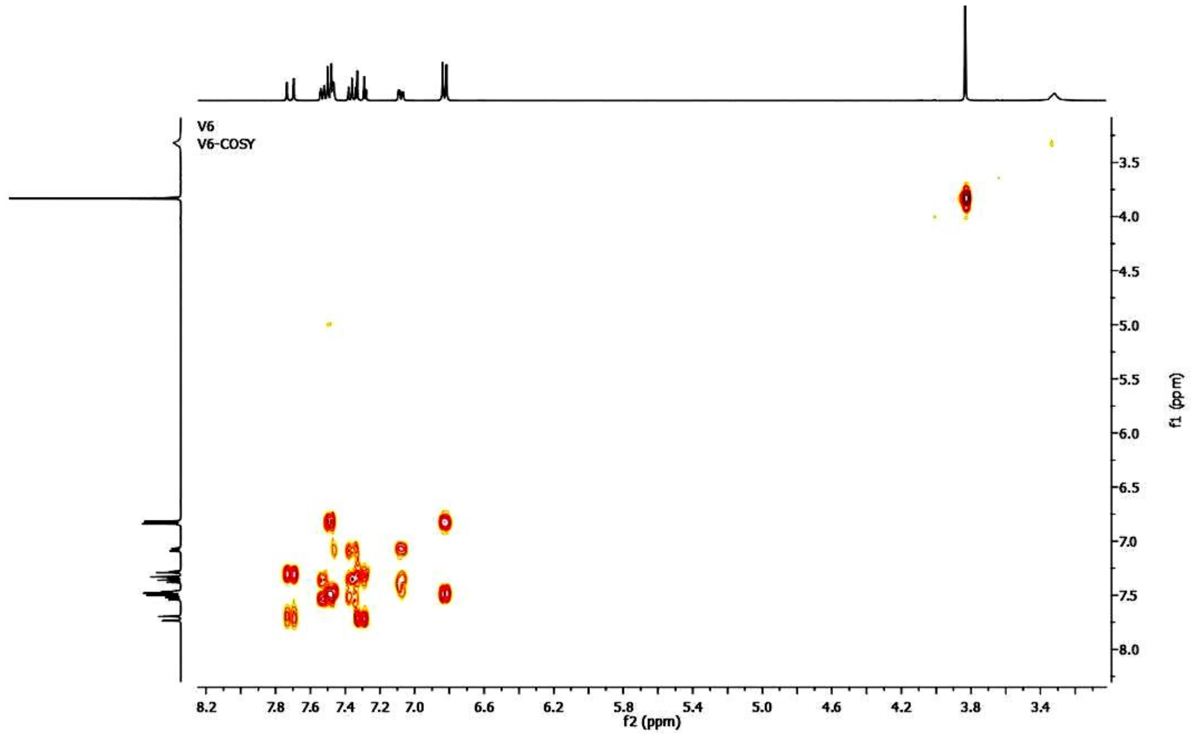

Figure S16. ^{1}H-^{1}H COSY NMR spectrum of compound **1f** (400 MHz, $CDCl_3$/CD_3OD, ppm)

1g: *(2E)-3-(2-Hydroxyphenyl)-1-(2-methoxyphenyl) prop-2-en-1-one*

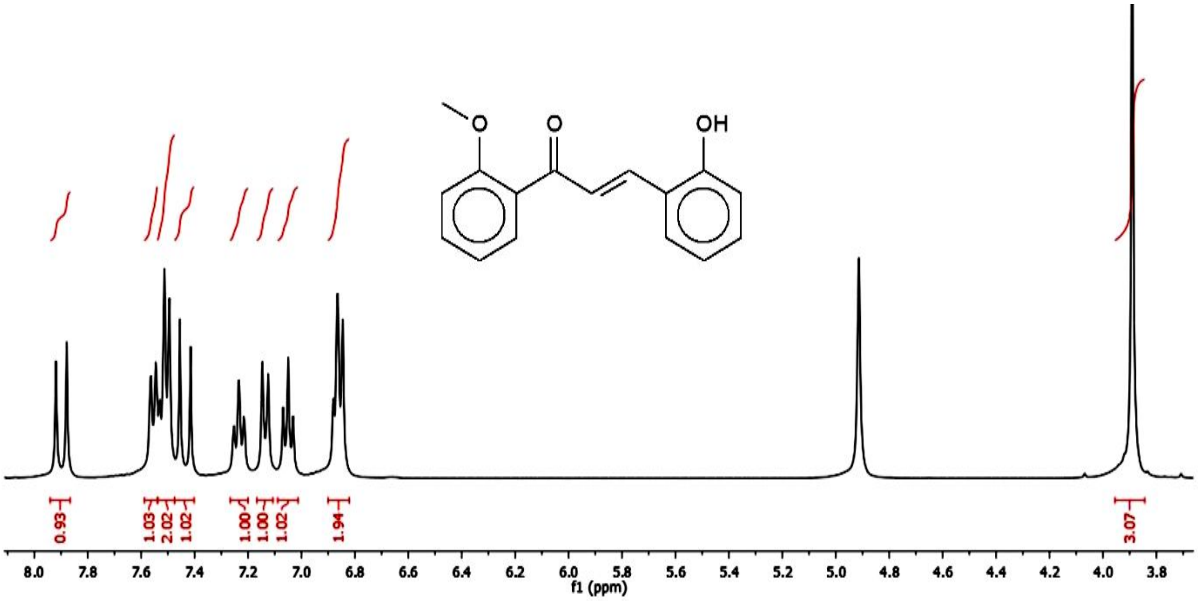

Figure S17. ^{1}H-NMR spectrum of compound **1g** (400 MHz, $CDCl_3/CD_3OD$, ppm)

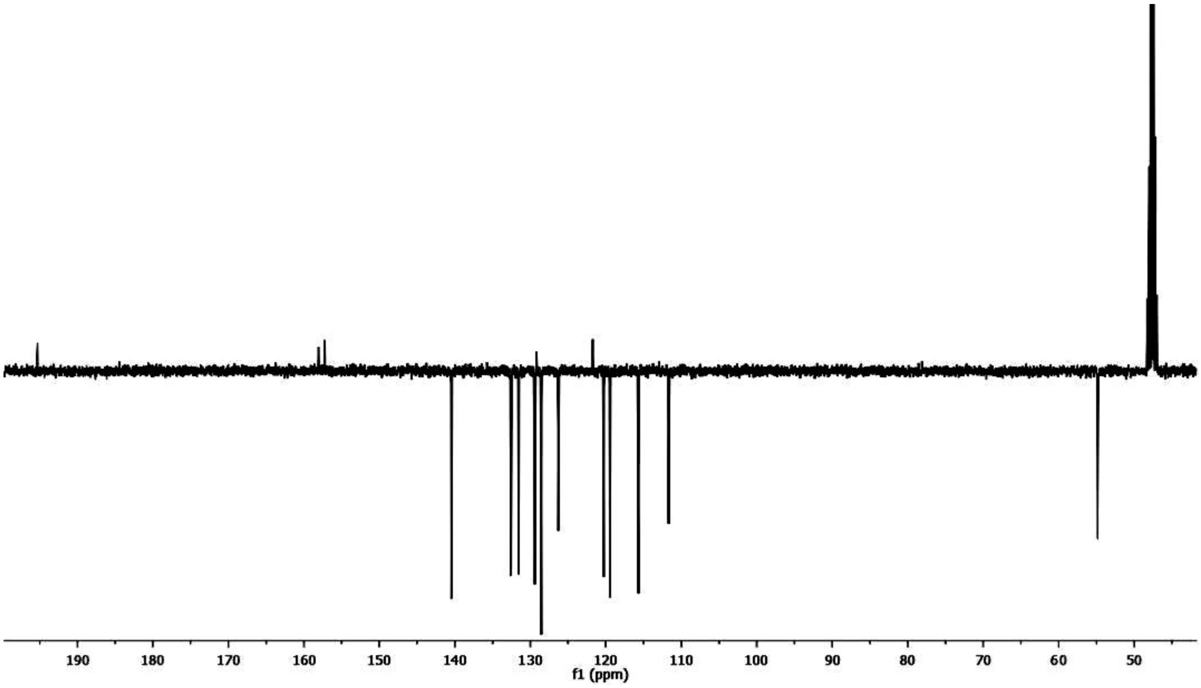

Figure S18. ^{13}C-APT NMR spectrum of compound 1g (100 MHz, CD3OD, ppm)

1h: *(2E)-3-(3-Hydroxyphenyl)-1-(2-methoxyphenyl) prop-2-en-1-one*

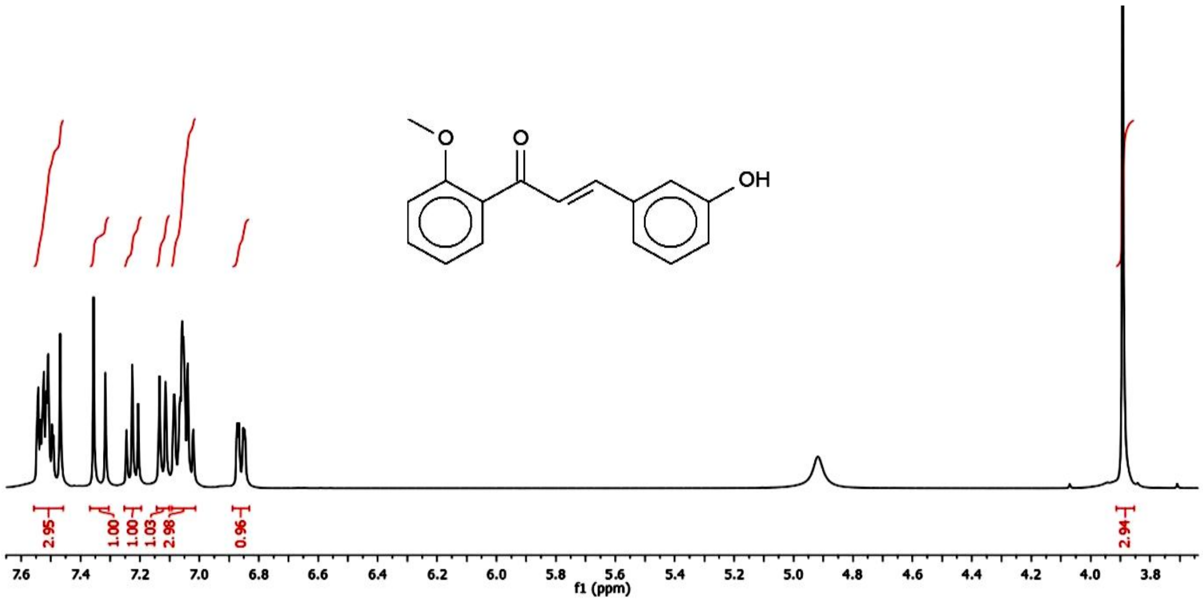

Figure S19. ^{1}H-NMR spectrum of compound **1h** (400 MHz, $CDCl_3/CD_3OD$, ppm)

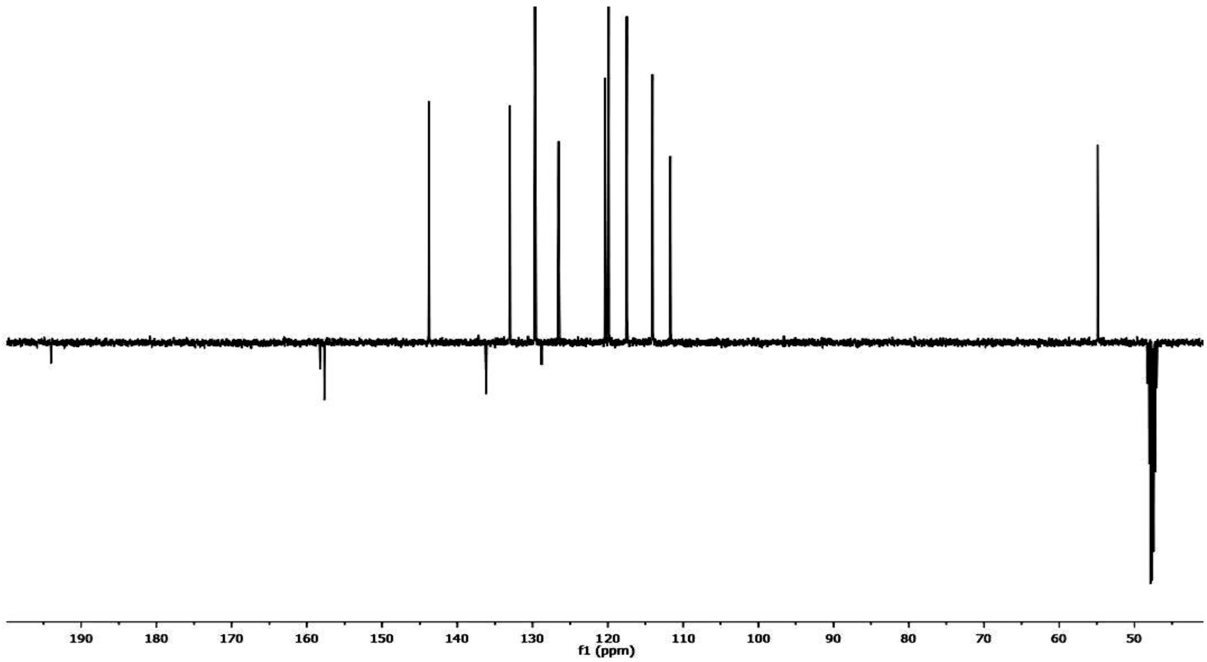

Figure S20. ^{13}C-APT NMR spectrum of compound **1h** (100 MHz, CD_3OD, ppm)

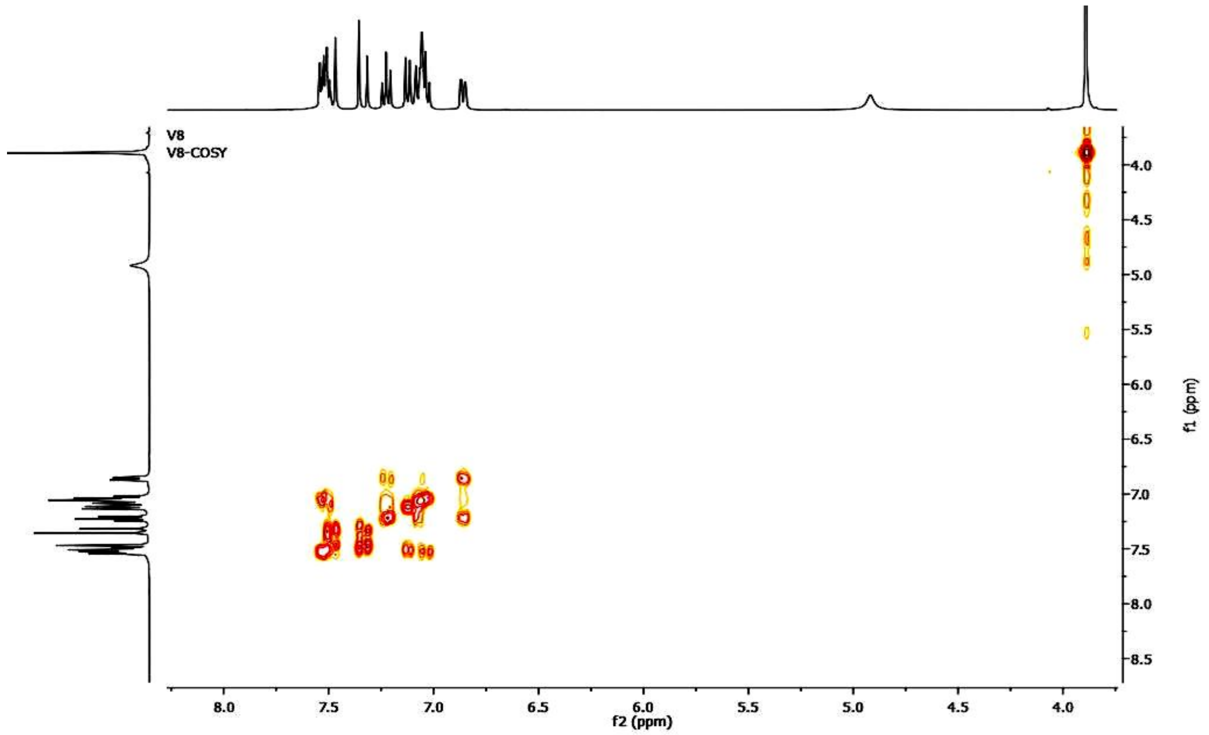

Figure S21. ^{1}H-^{1}H COSY NMR spectrum of compound **1h** (400 MHz, $CDCl_3/CD_3OD$, ppm)

1i: *(2E)-3-(4-Hydroxyphenyl)-1-(2-methoxyphenyl) prop-2-en-1-one*

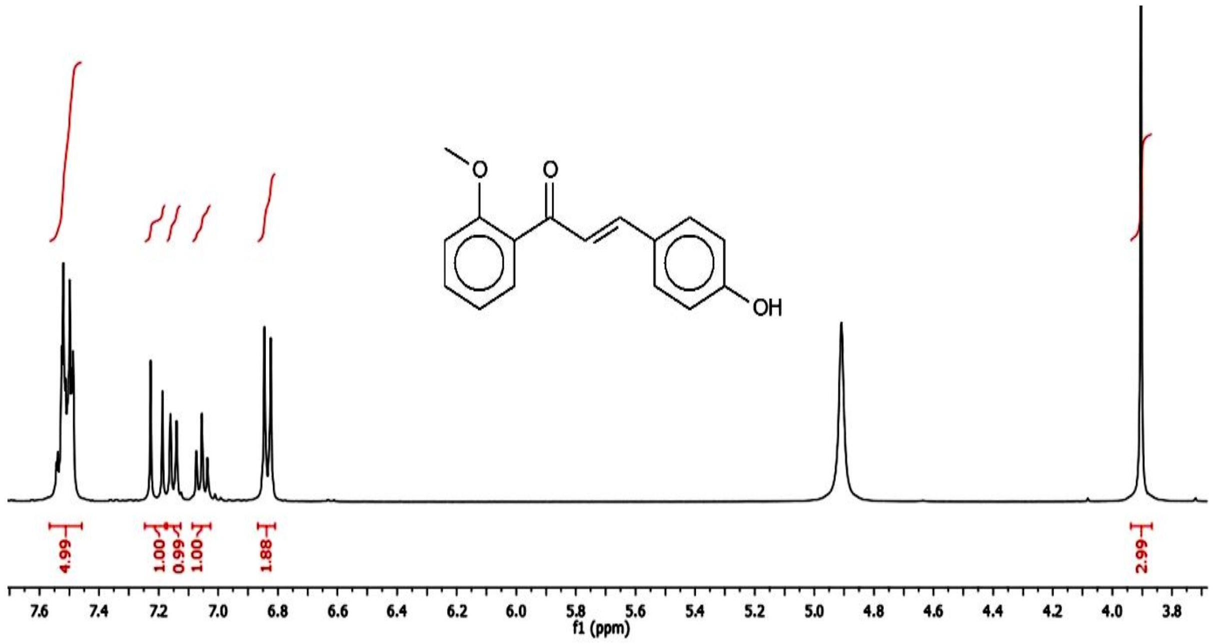

Figure S22. ^{1}H-NMR spectrum of compound **1i** (400 MHz, $CDCl_3/CD_3OD$, ppm)

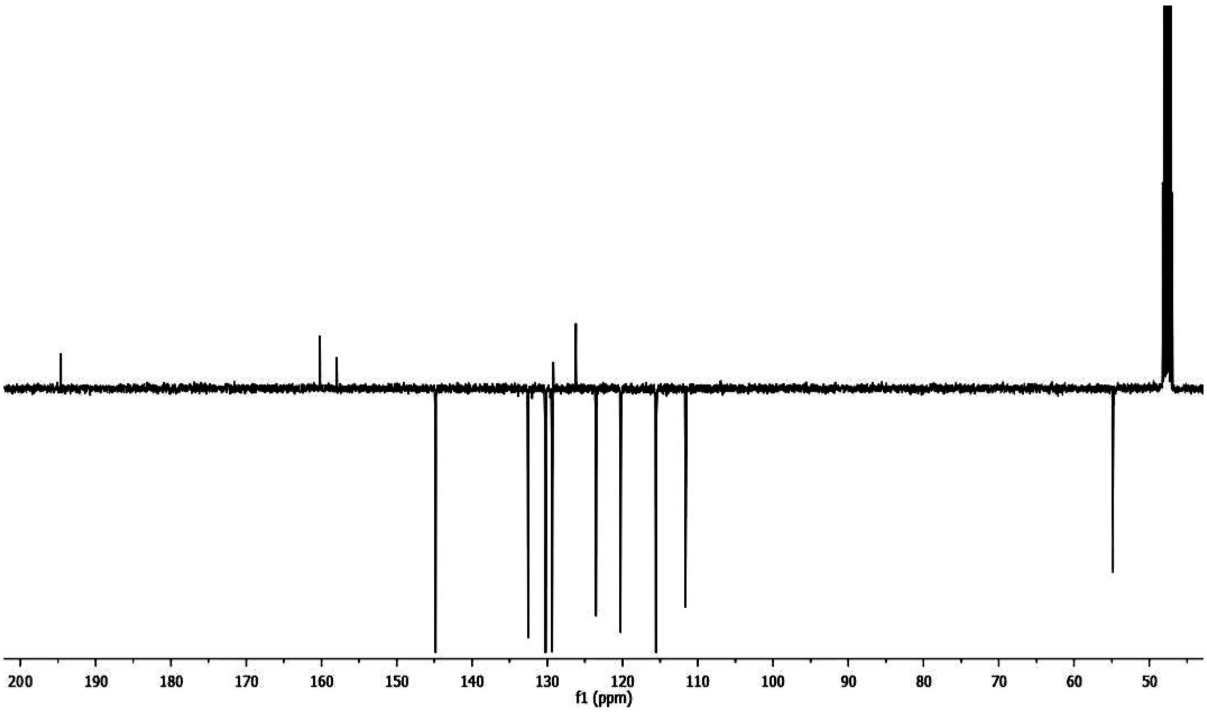

Figure S23. ^{13}C-APT NMR spectrum of compound **1i** (100 MHz, CD3OD, ppm)

Gypsogenin (2): *3-Hydroxy-23-oxoolean-12-en-28-oic acid*

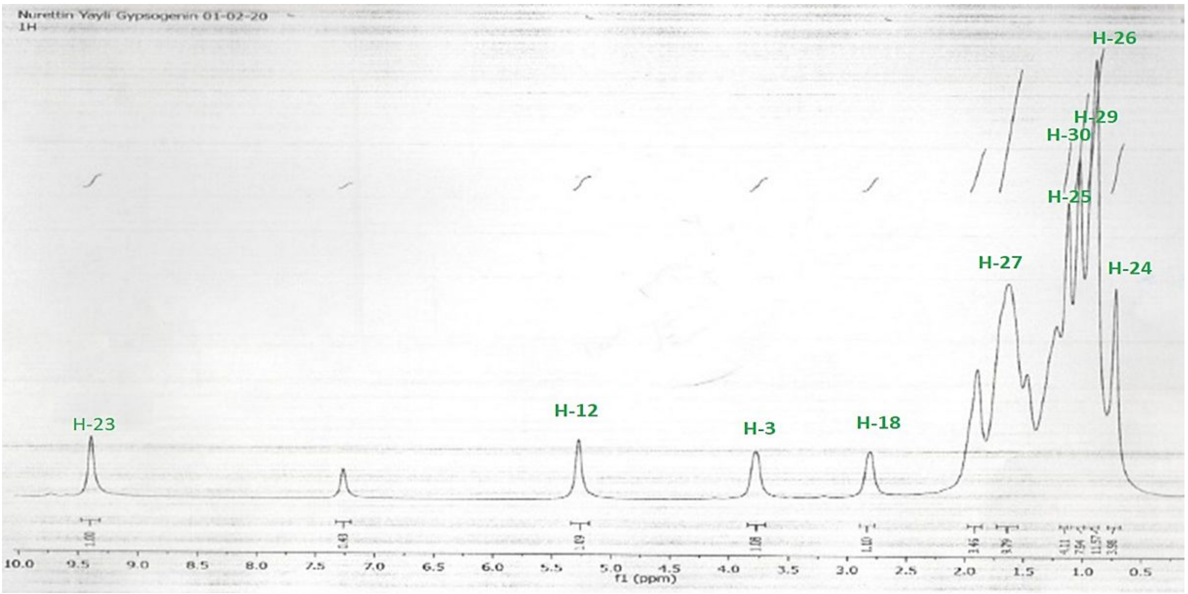

Figure S24. ^{1}H-NMR spectrum of compound **2** (400 MHz, C_5D_5N, ppm)

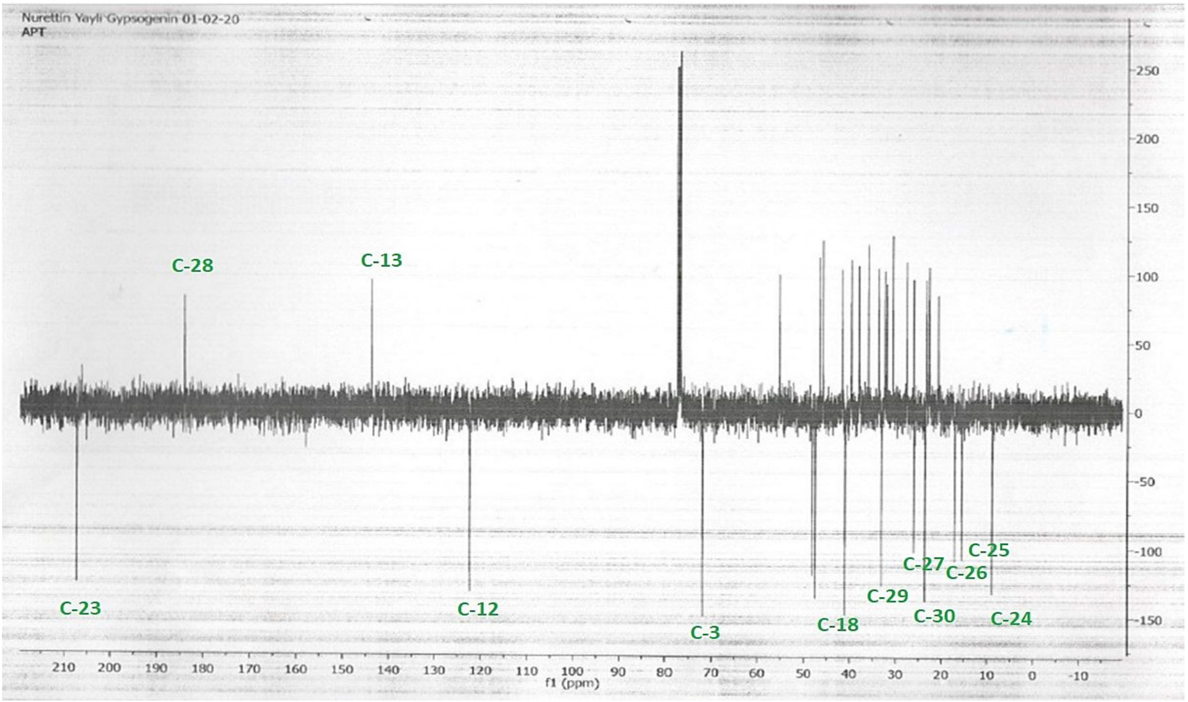

Figure S25. ^{13}C-APT NMR spectrum of compound **2** (100 MHz, C_5D_5N, ppm)

Acetyl-Gypso (3) : *3-(Acetyloxy)-23-oxoolean-12-en-28-oic acid*

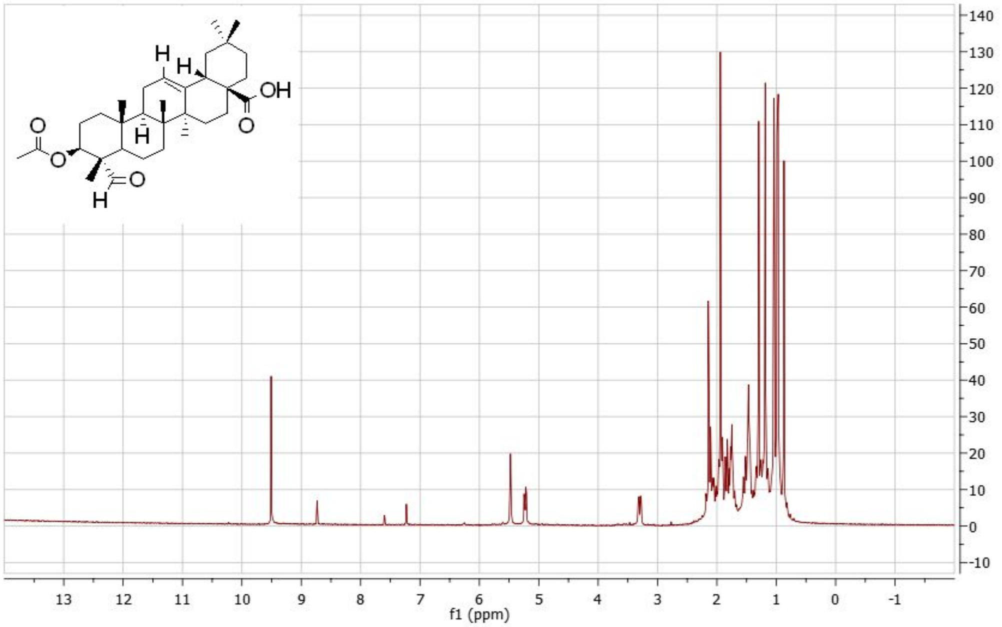

Figure S26. ^{1}H-NMR spectrum of compound **3** (400 MHz, C_5D_5N, ppm)

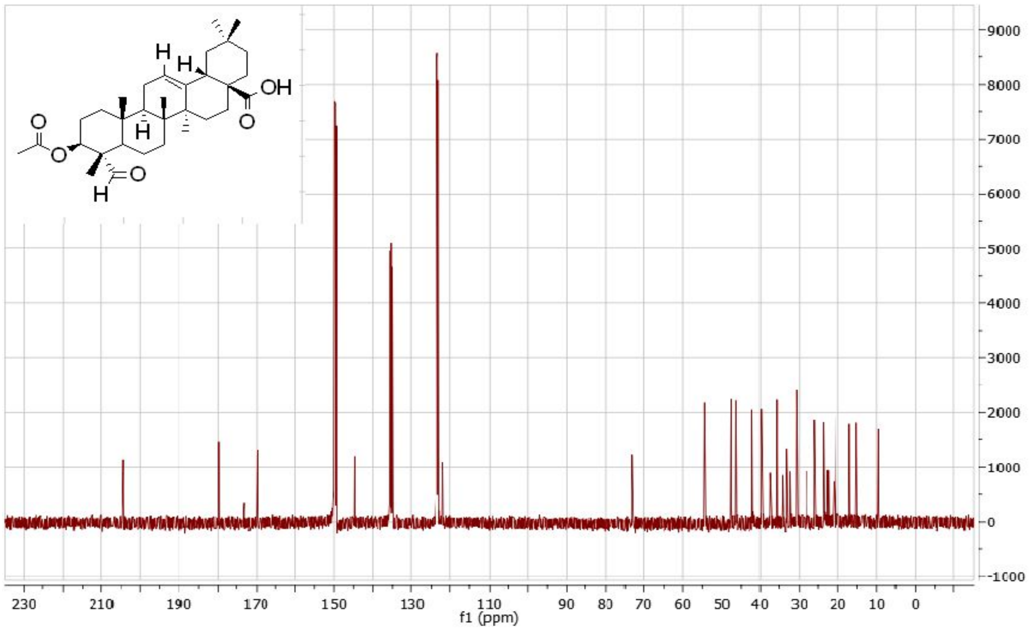

Figure S27. ^{13}C NMR spectrum of compound **3** (100 MHz, C_5D_5N, ppm)

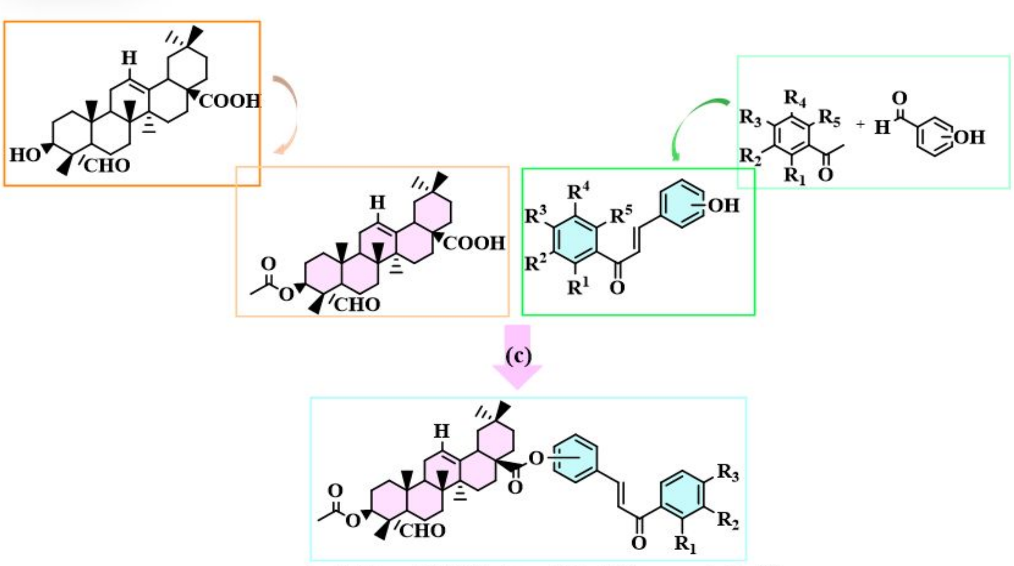

Compounds no

Acetyl Gypso-COO-Chalcone Hybrid Compounds (4a-4i)

	-OH	*R¹*	*R²*	*R³*	*R⁴*	*R⁵*
**4a-4c**	-o, -m, -p	-H	-H	-**OCH₃**	-H	-H
**4d-4f**	-o, -m, -p	-H	-**OCH₃**	-H	-H	-H
**4g-4i**	-o, -m, -p	-**OCH₃**	-H	-H	-H	-H

4a: Acetyl Gypso-COO-Chalcone Hybrid Compound

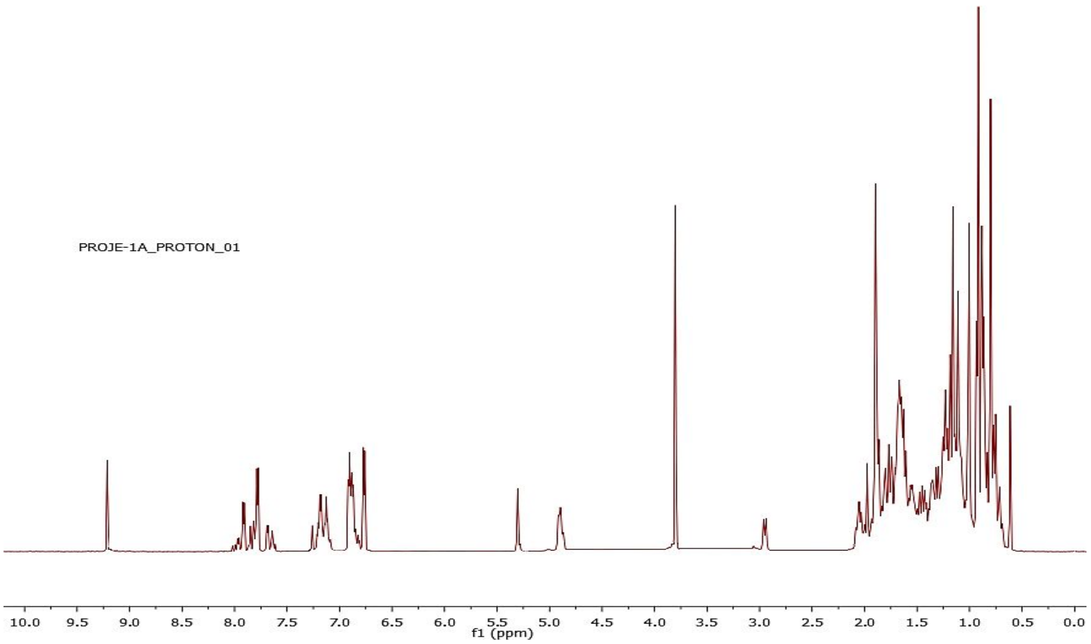

Figure S28. ^{1}H-NMR spectrum of compound **4a** (600 MHz, $CDCl_3$, ppm)

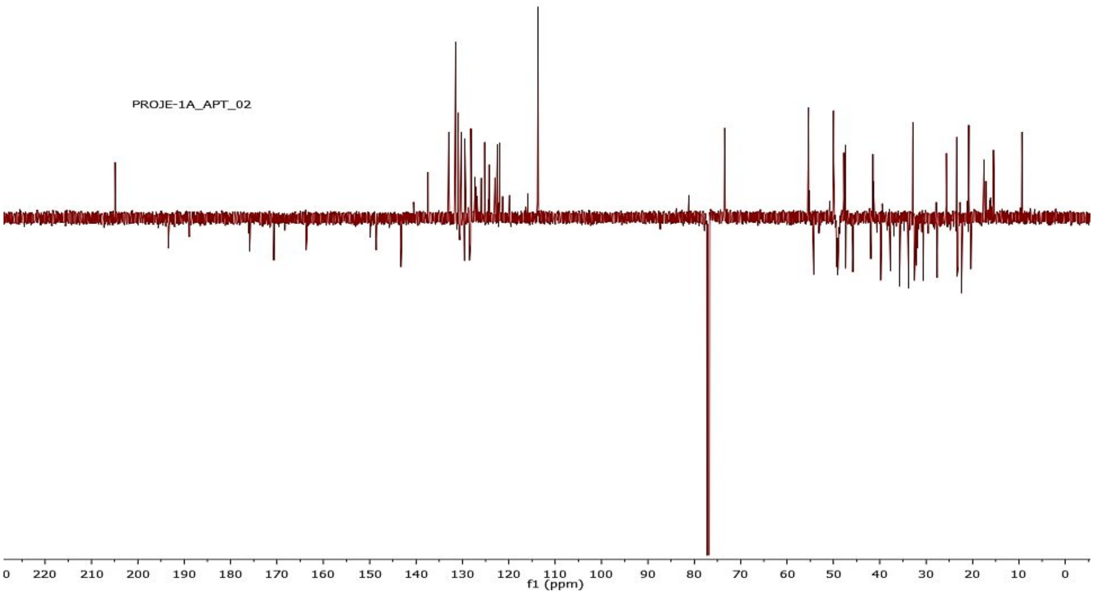

Figure S29. ^{13}C-APT NMR spectrum of compound **4a** (150 MHz, $CDCl_3$, ppm)

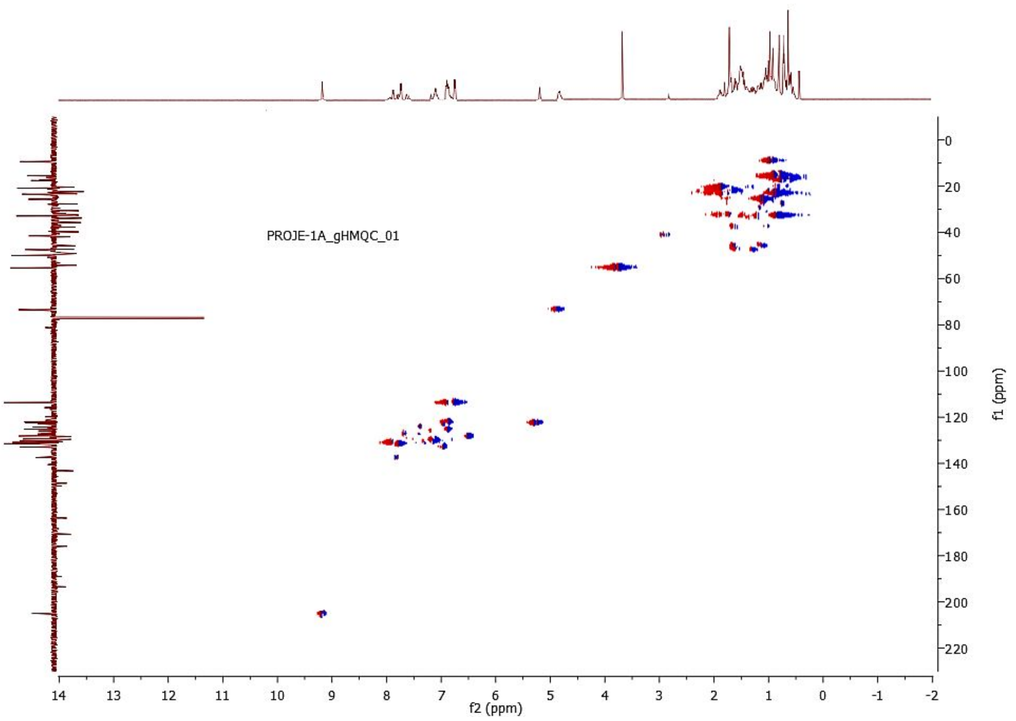

Figure S30. HMQC spectrum of compound **4a**

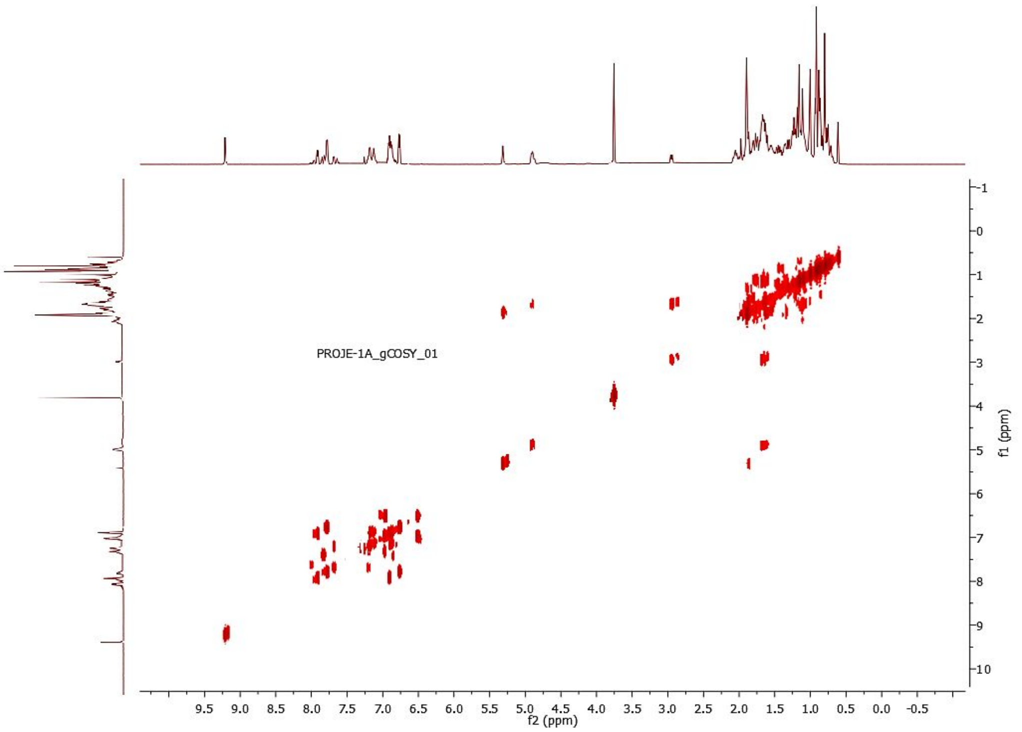

Figure S31. ^{1}H-^{1}H COSY NMR spectrum of compound **4a**

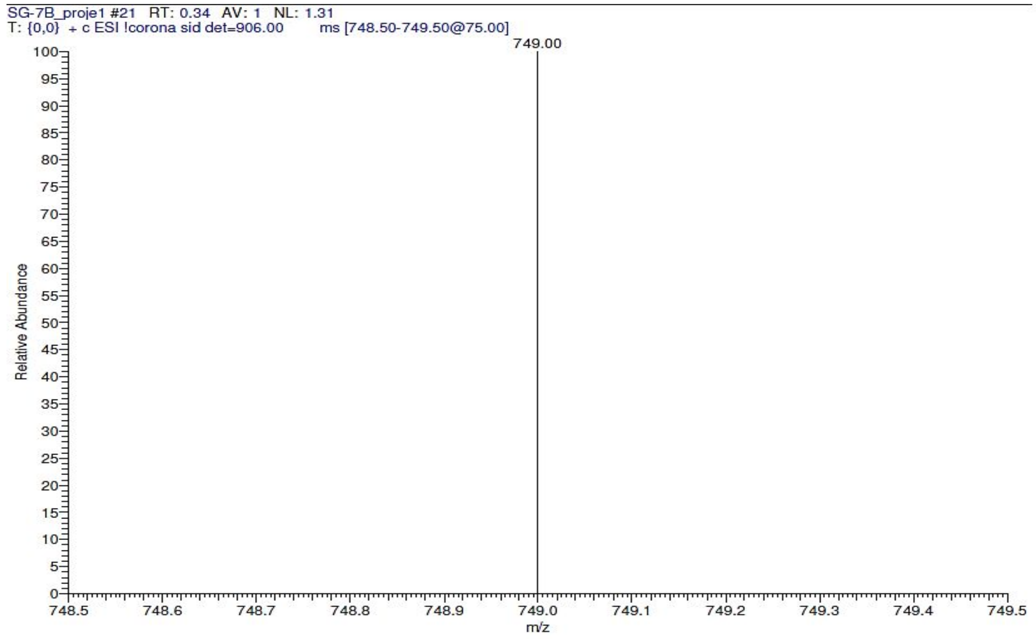

Figure S32. Mass spectrum of compound **4a** (Thermo Scientific/ Surveyor MSQ)

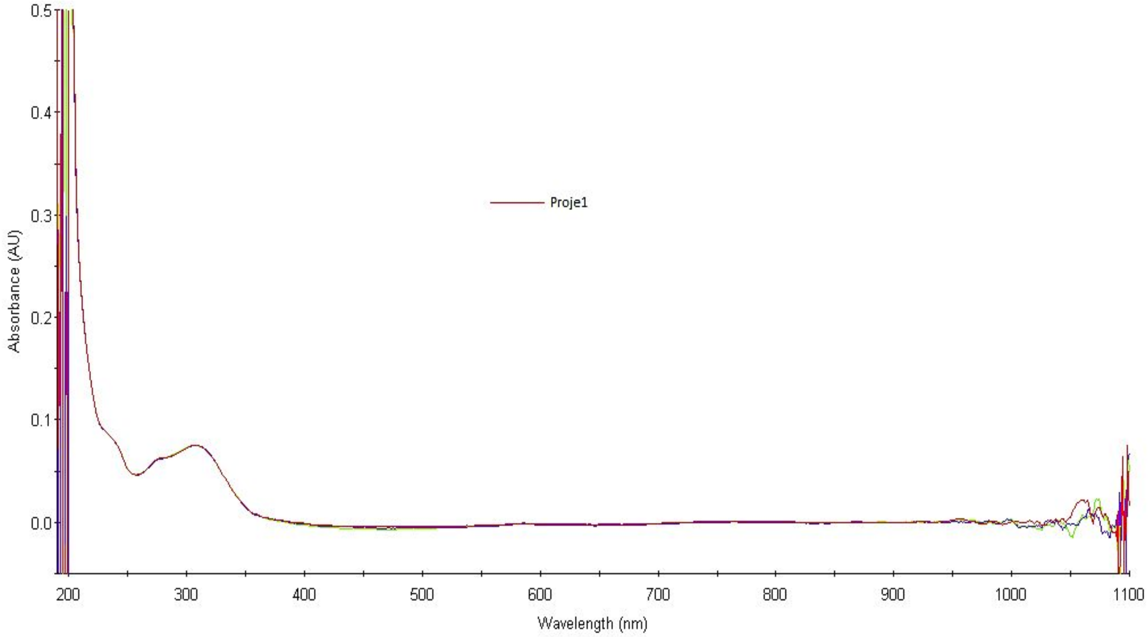

Figure S33. UV spectrum of compound **4a**

4b: Acetyl Gypso-COO-Chalcone Hybrid Compound

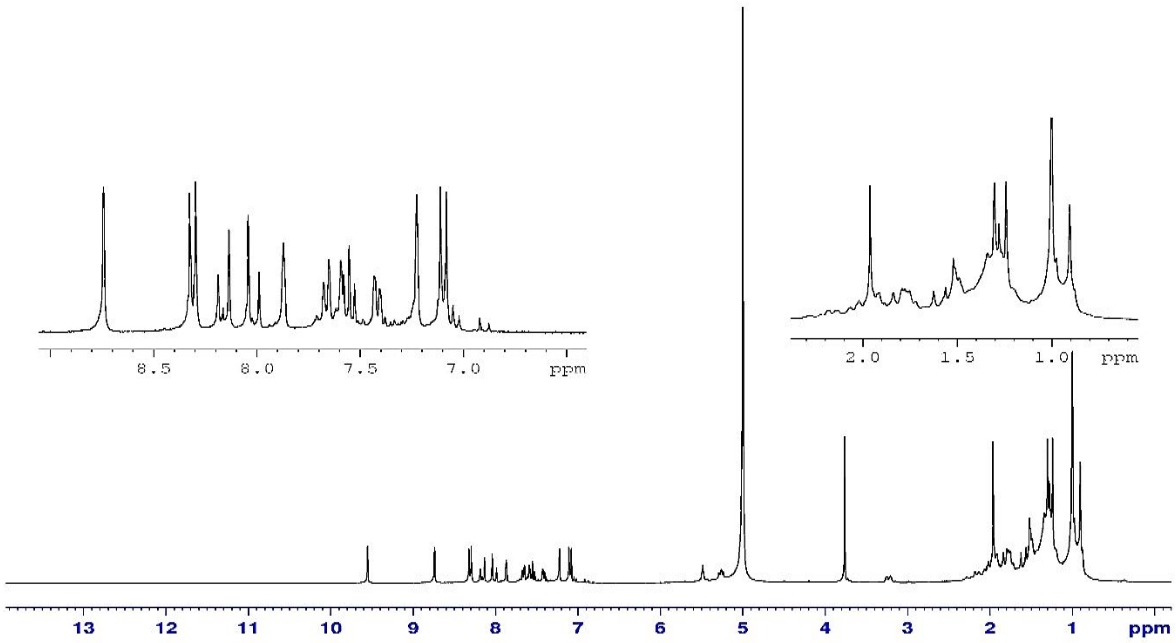

Figure S34. ^{1}H-NMR spectrum of compound **4b** (600 MHz, $CDCl_3$, ppm)

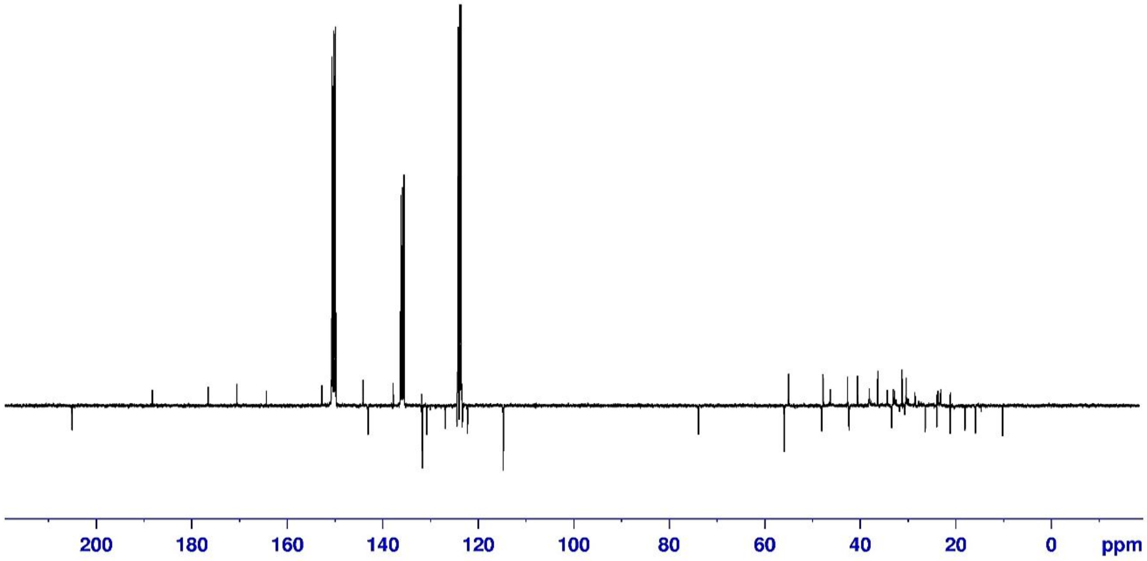

Figure S35. APT-NMR spectrum of compound **4b** (150 MHz, $CDCl_3$, ppm)

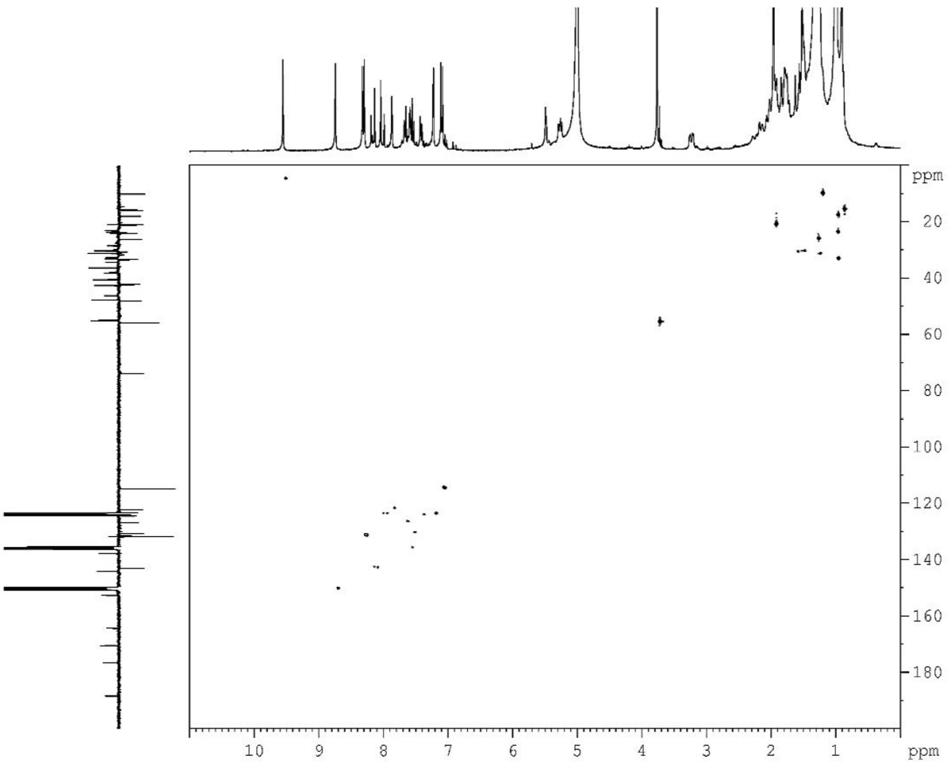

Figure S36. HMQC spectrum of compound **4b**

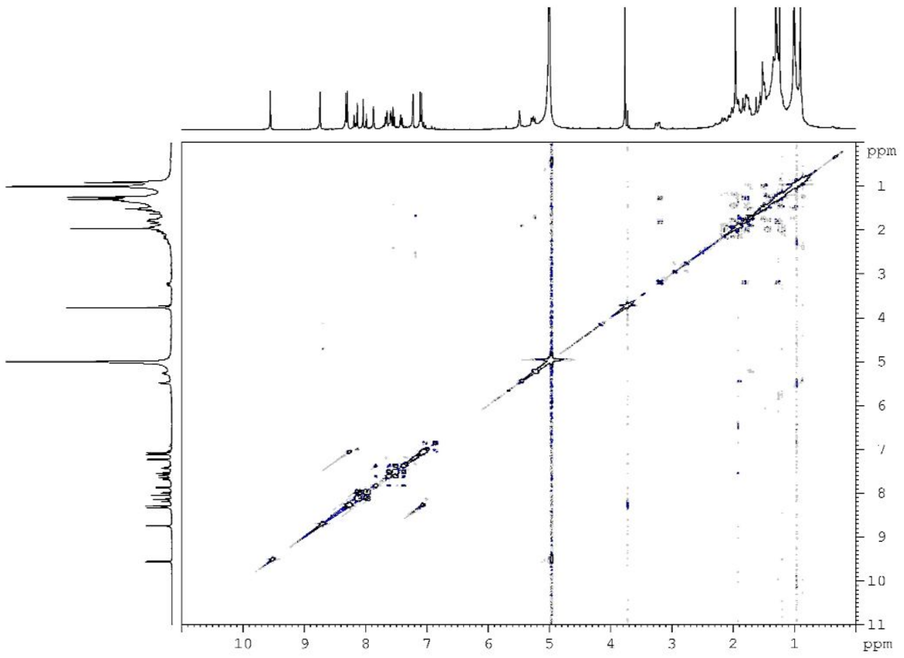

Figure S37. ^{1}H-^{1}H COSY-NMR spectrum of compound **4b** (400 MHz, CD_3OD, ppm)

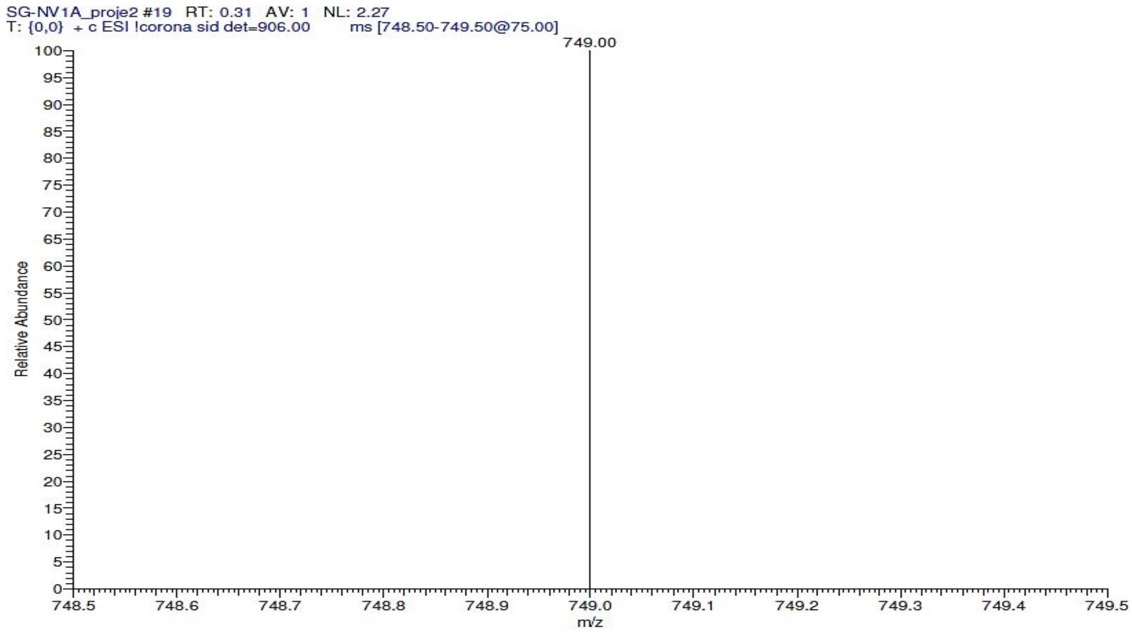

Figure S38. Mass spectrum of compound **4b** (Thermo Scientific/ Surveyor MSQ)

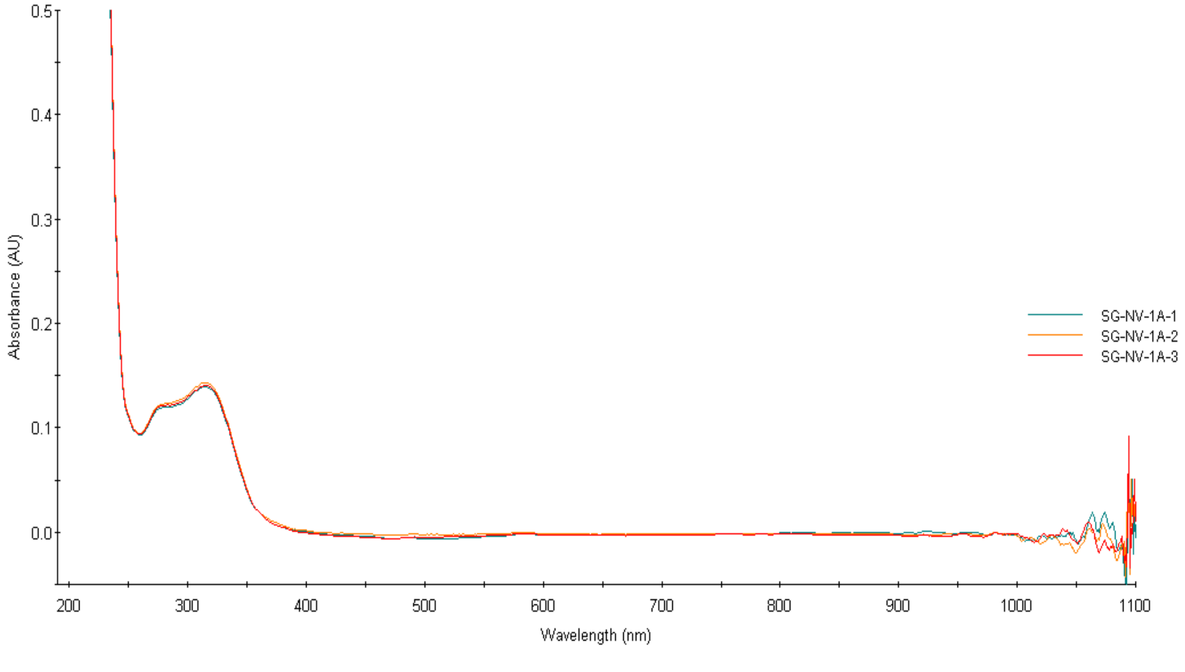

Figure S39. UV spectrum of compound **4b**

4c: Acetyl Gypso-COO-Chalcone Hybrid Compound

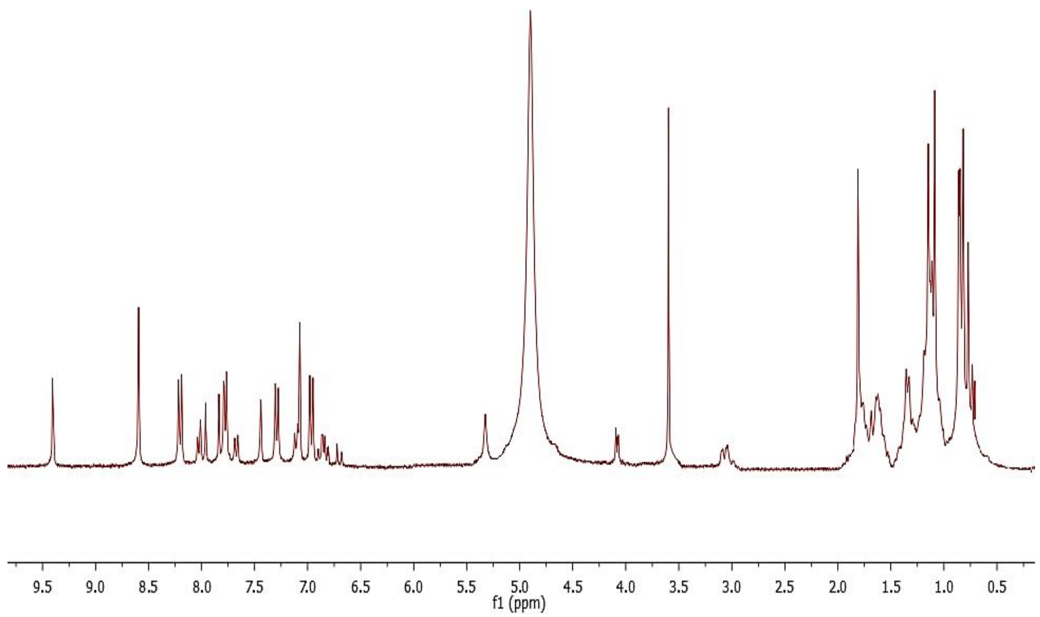

Figure S40. ^{1}H-NMR spectrum of compound **4c** (600 MHz, $CDCl_3$, ppm)

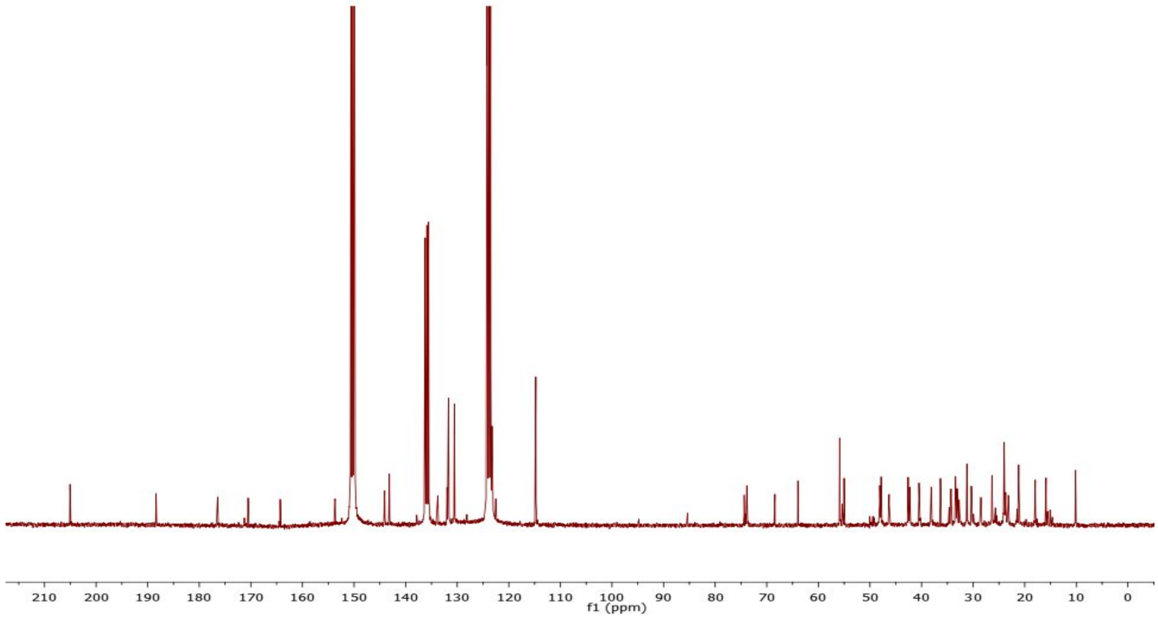

Figure S41. ^{13}C-NMR spectrum of compound **4c** (150 MHz, $CDCl_3$, ppm)

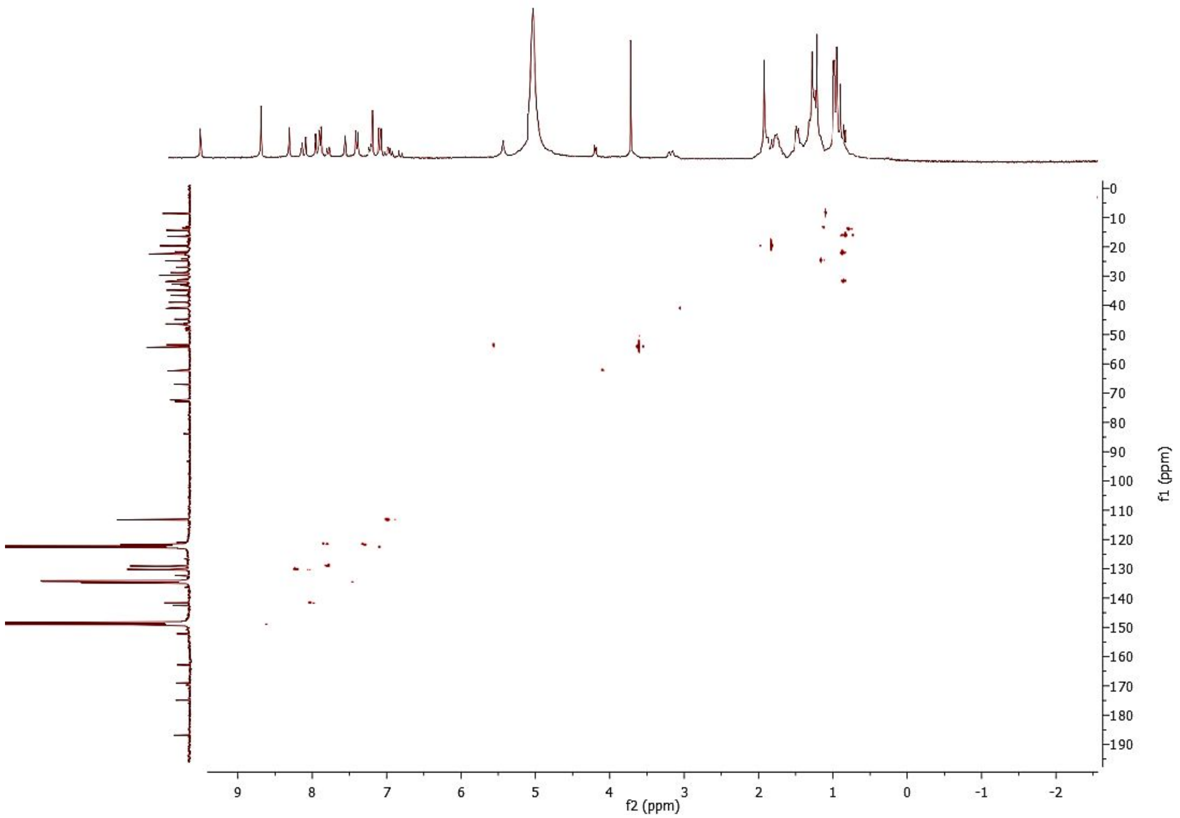

Figure S42. HMQC spectrum of compound **4c**

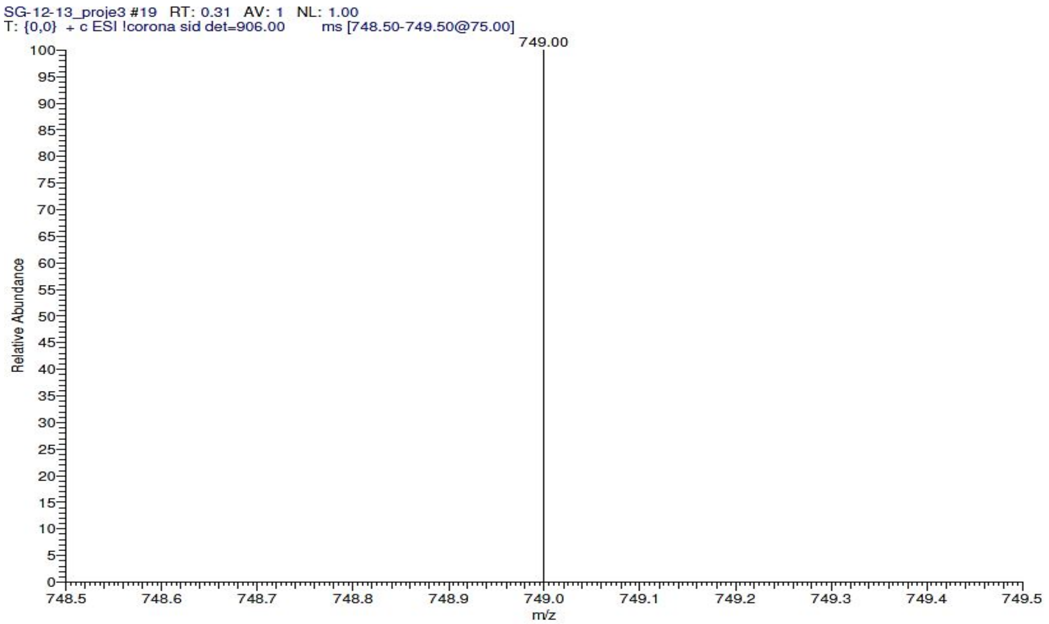

Figure S43. Mass spectrum of compound **4c** (Thermo Scientific/ Surveyor MSQ)

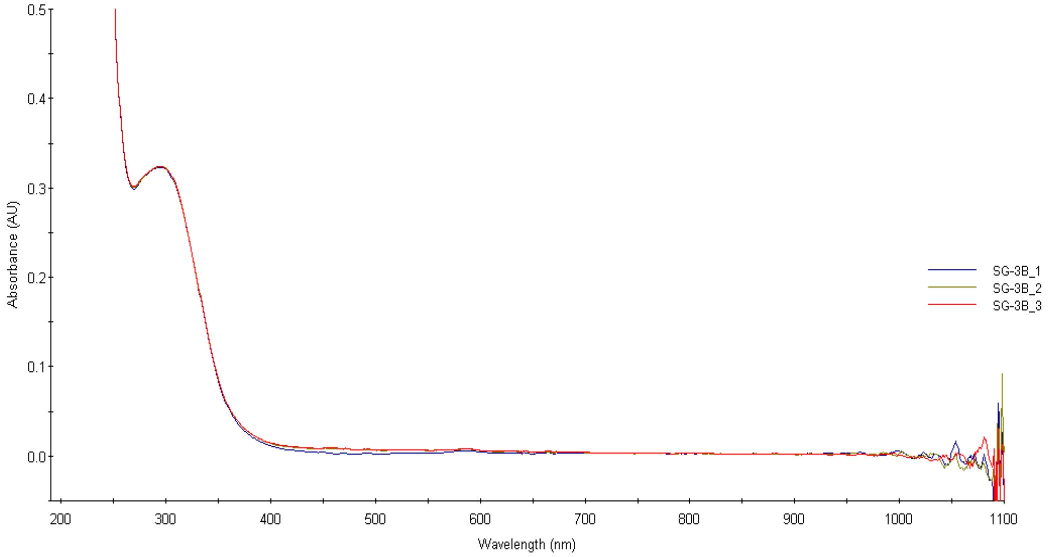

Figure S44. UV spectrum of compound **4c**

4d: Acetyl Gypso-COO-Chalcone Hybrid Compound

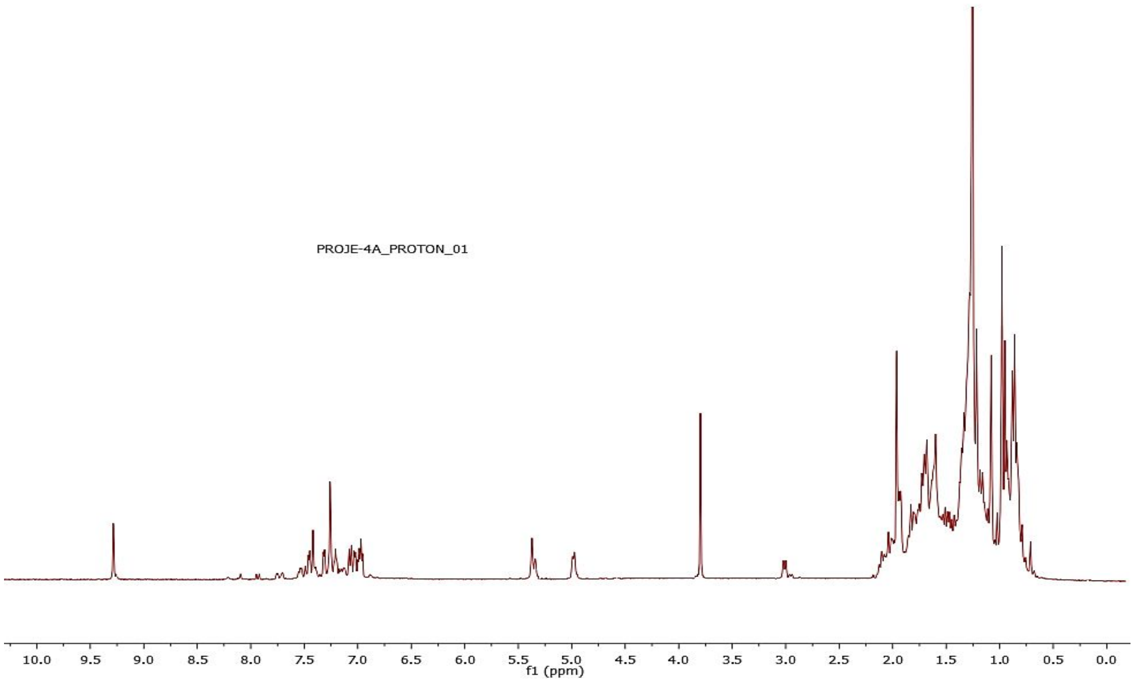

Figure S45. ^{1}H-NMR spectrum of compound **4d** (600 MHz, $CDCl_3$, ppm)

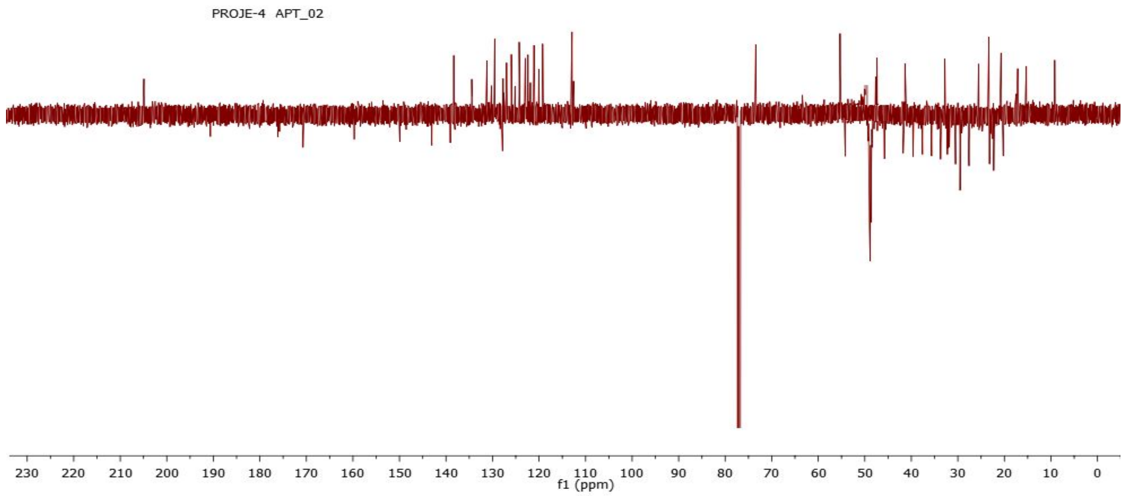

Figure S46. APT-NMR spectrum of compound **4d** (150 MHz, $CDCl_3$, ppm)

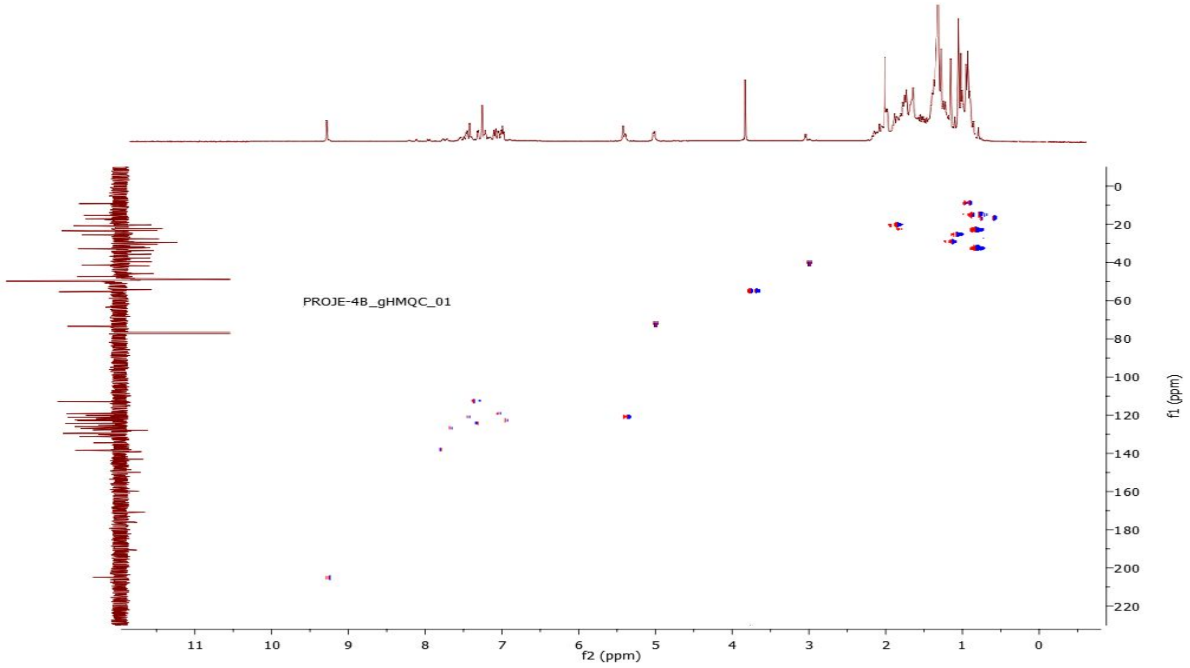

Figure S47. HMQC spectrum of compound **4d**

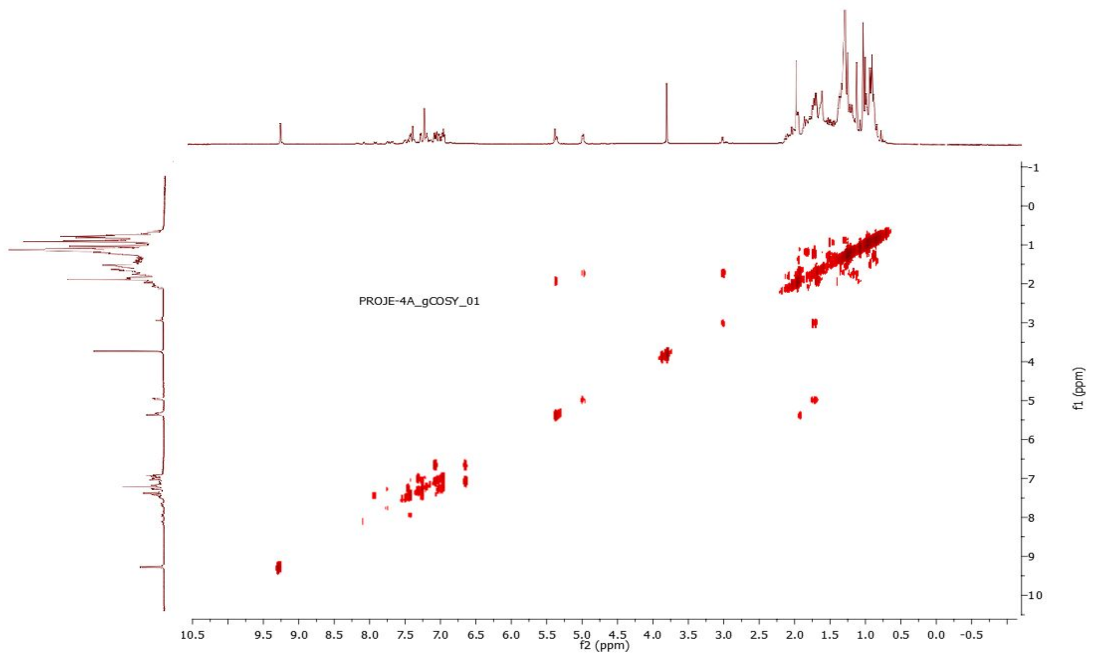

Figure S48. ^{1}H-^{1}H COSY-NMR spectrum of compound **4d (**400 MHz, CD_3OD, ppm)

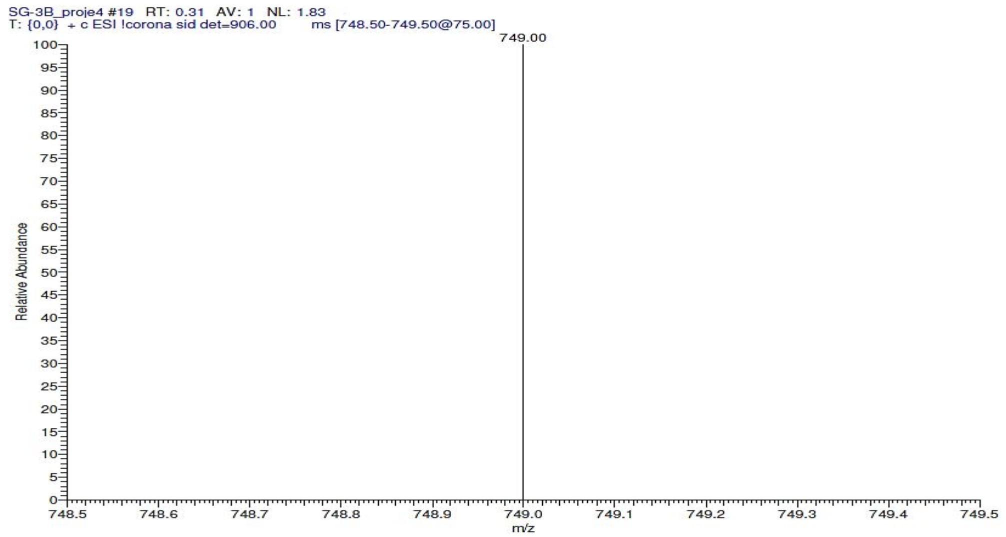

Figure S49. Mass spectrum of compound **4d** (Thermo Scientific/ Surveyor MSQ)

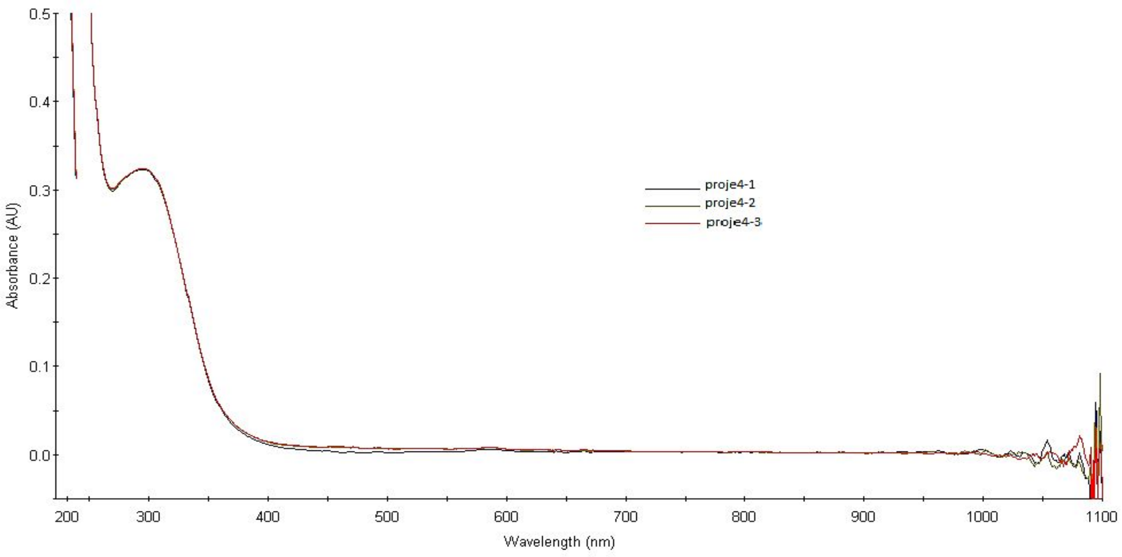

Figure S50. UV spectrum of compound **4d**

4e: Acetyl Gypso-COO-Chalcone Hybrid Compound

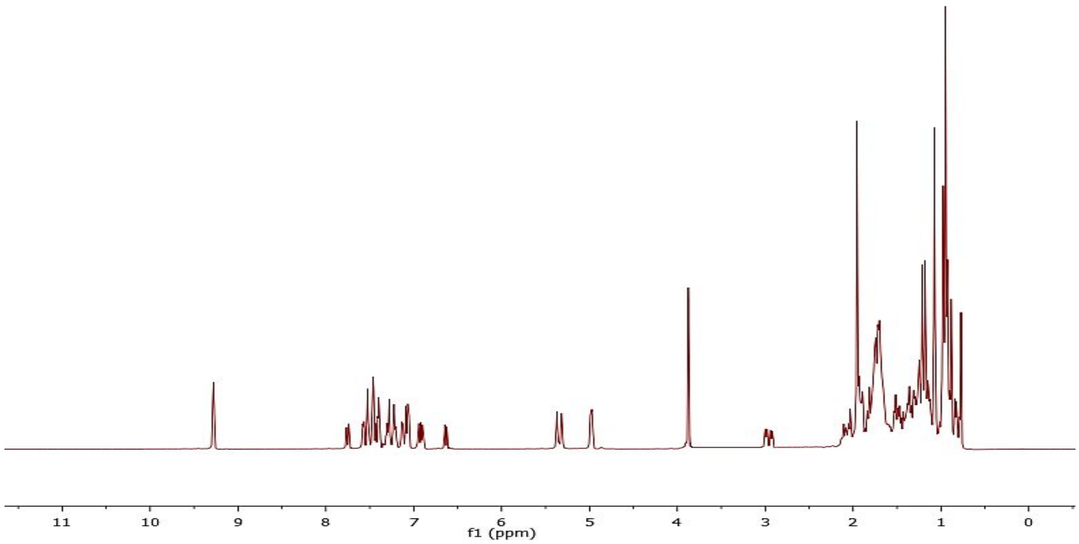

Figure S51. ^{1}H-NMR spectrum of compound **4e** (600 MHz, $CDCl_3$, ppm)

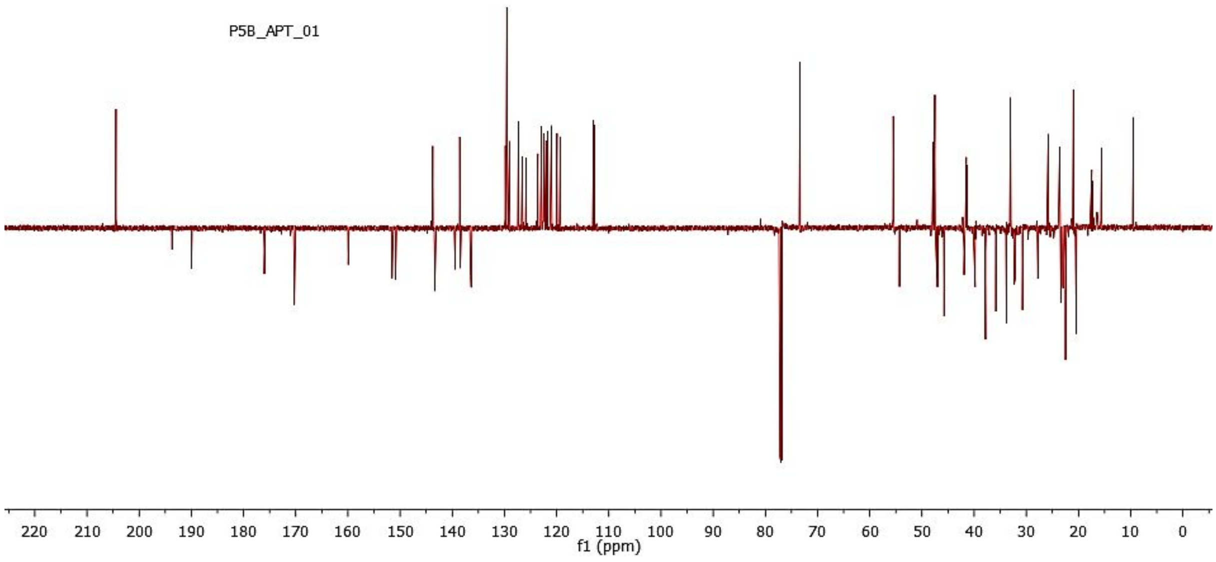

Figure S52. APT-NMR spectrum of compound **4e** (150 MHz, $CDCl_3$, ppm)

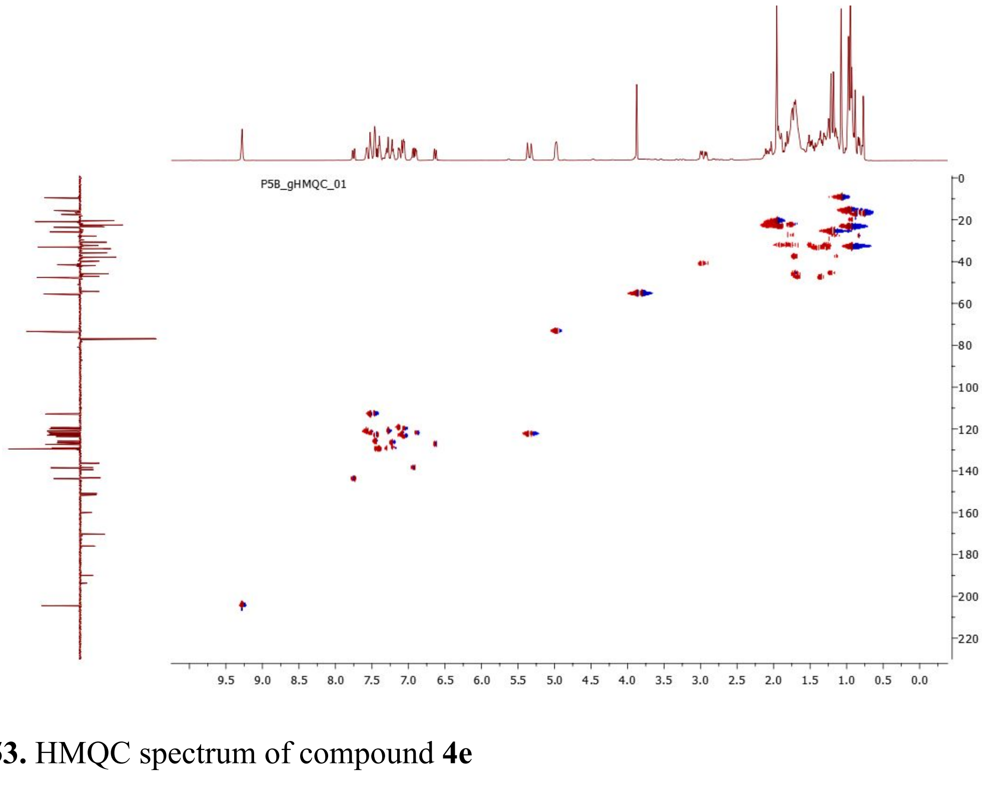

Figure S53. HMQC spectrum of compound **4e**

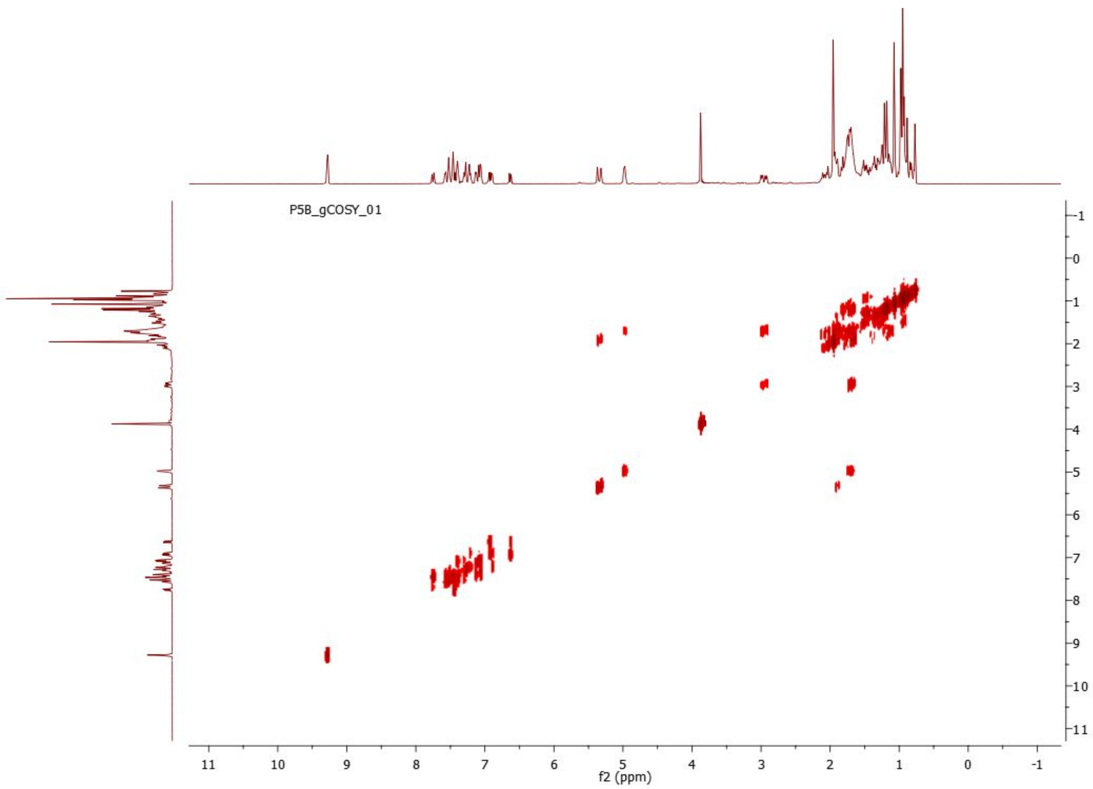

Figure S54. ^{1}H-^{1}H COSY-NMR spectrum of compound **4e** (400 MHz, CD_3OD, ppm)

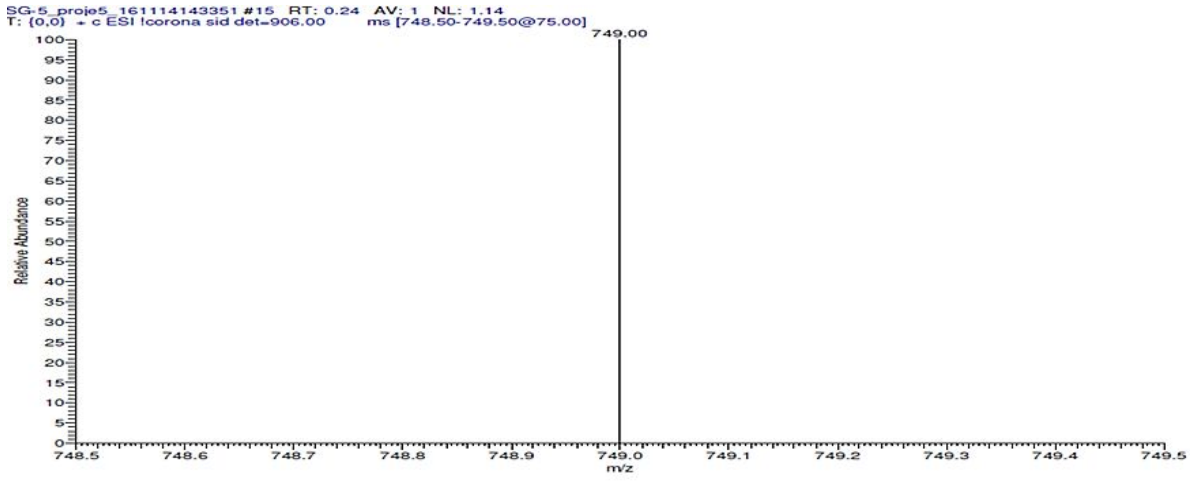

Figure S55. Mass spectrum of compound **4e** (Thermo Scientific/ Surveyor MSQ)

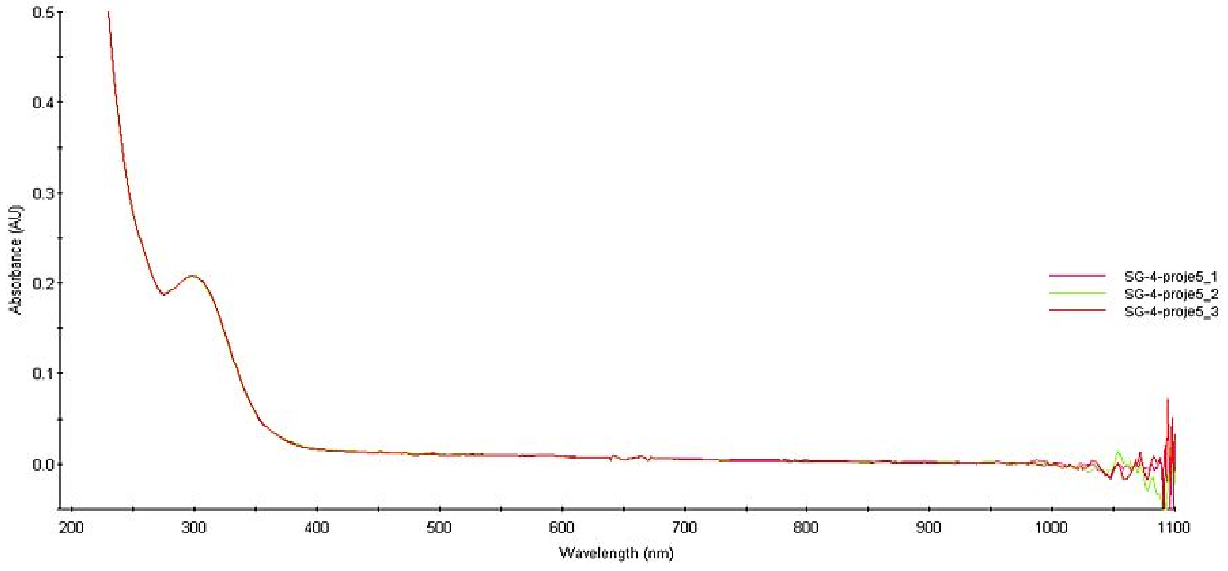

Figure S56. UV spectrum of compound **4e**

4f: Acetyl Gypso-COO-Chalcone Hybrid Compound

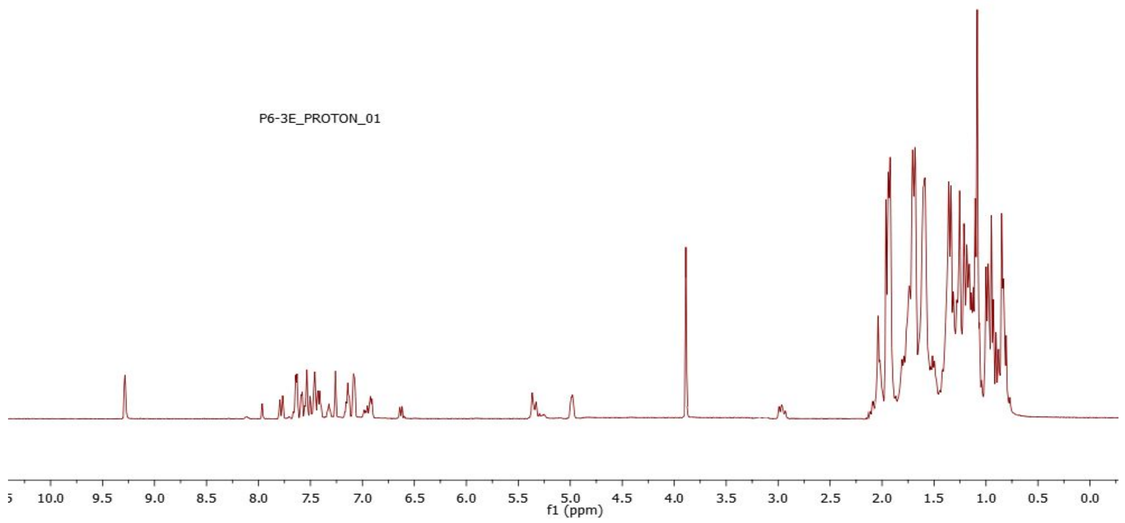

Figure S57. ^{1}H-NMR spectrum of compound **4f** (600 MHz, $CDCl_3$, ppm)

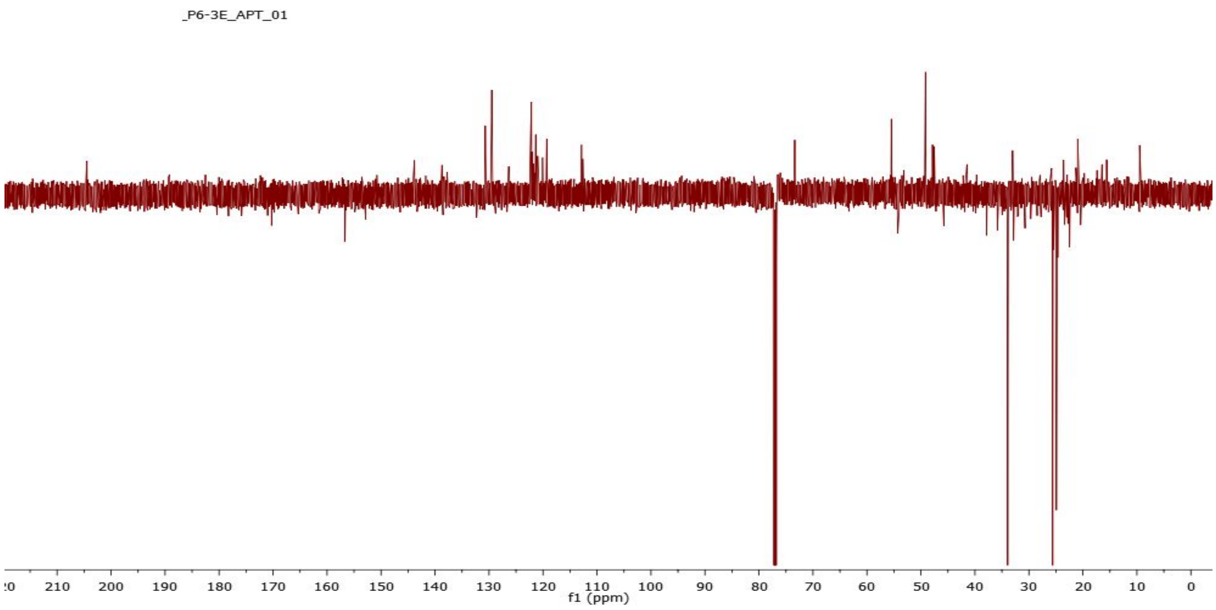

Figure S58. APT-NMR spectrum of compound **4f** (150 MHz, $CDCl_3$, ppm)

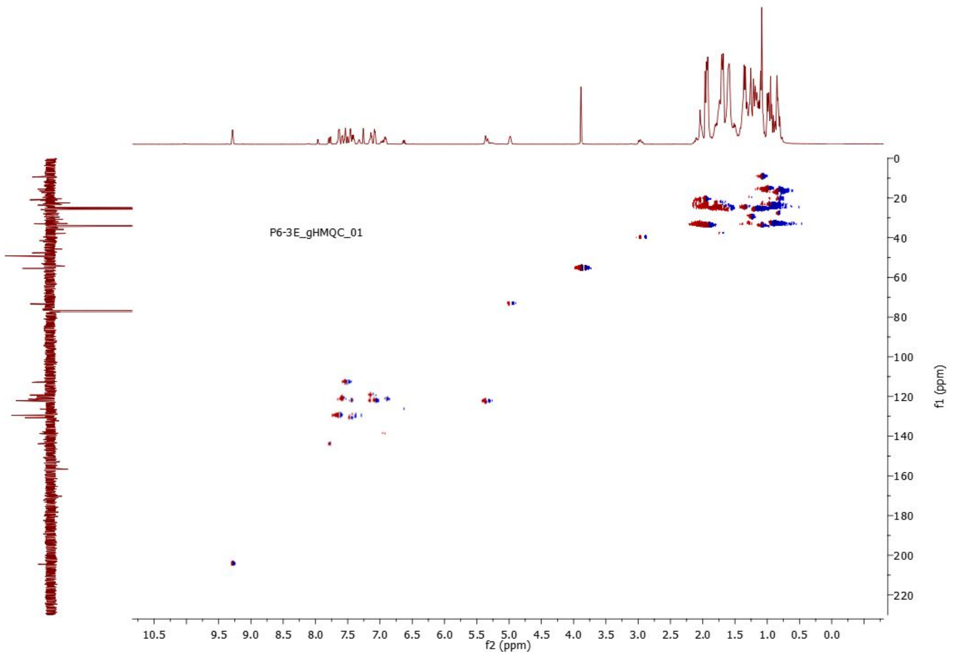

Figure S59. HMQC spectrum of compound **4f**

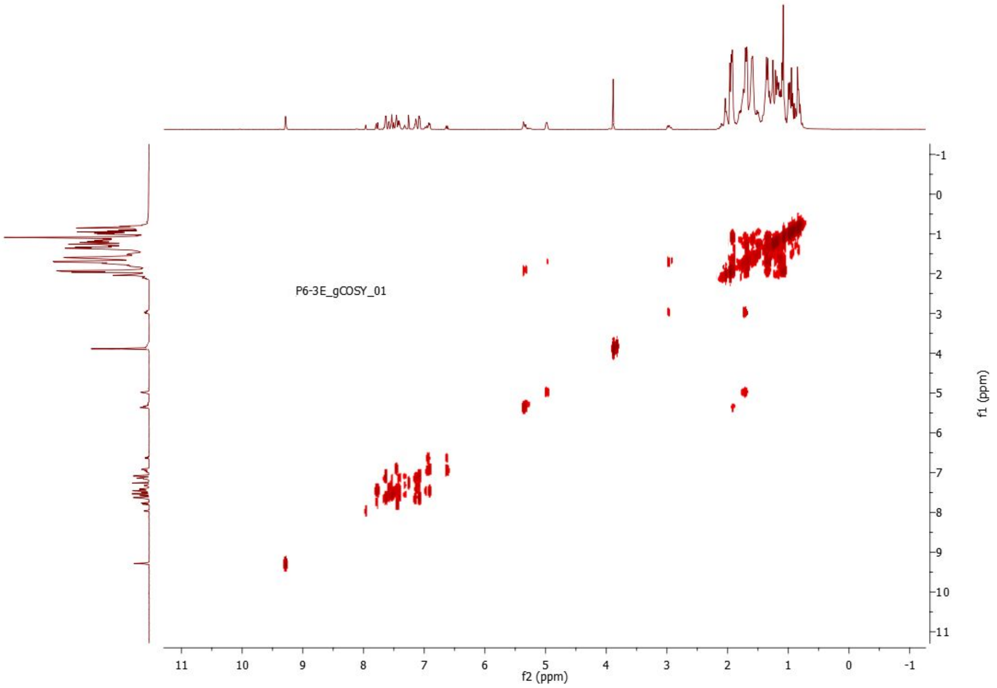

Figure S60. ^{1}H-^{1}H COSY-NMR spectrum of compound **4f (**400 MHz, CD_3OD, ppm)

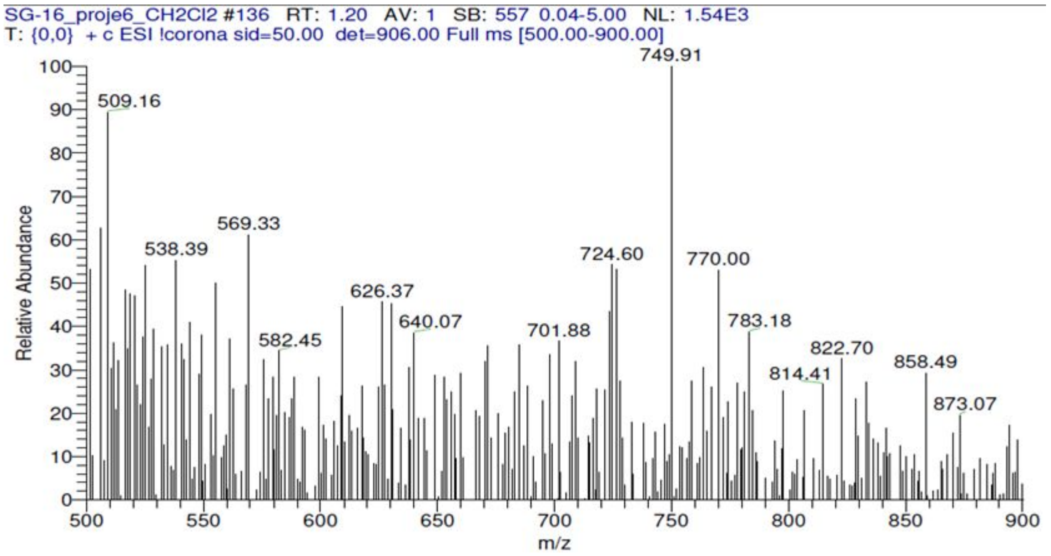

Figure S61. Mass spectrum of compound **4f** (Thermo Scientific/ Surveyor MSQ

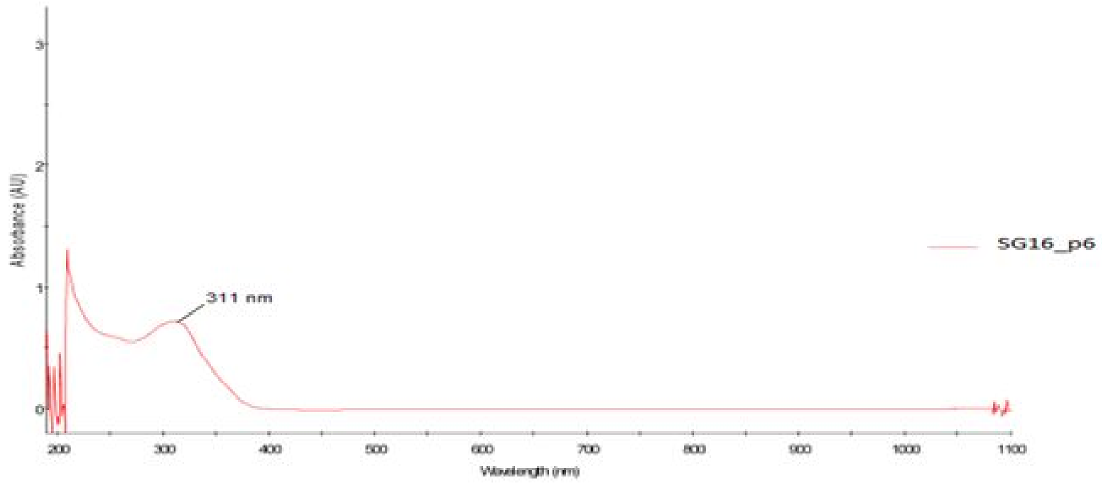

Figure S62. UV spectrum of compound **4f**

4g: Acetyl Gypso-COO-Chalcone Hybrid Compound

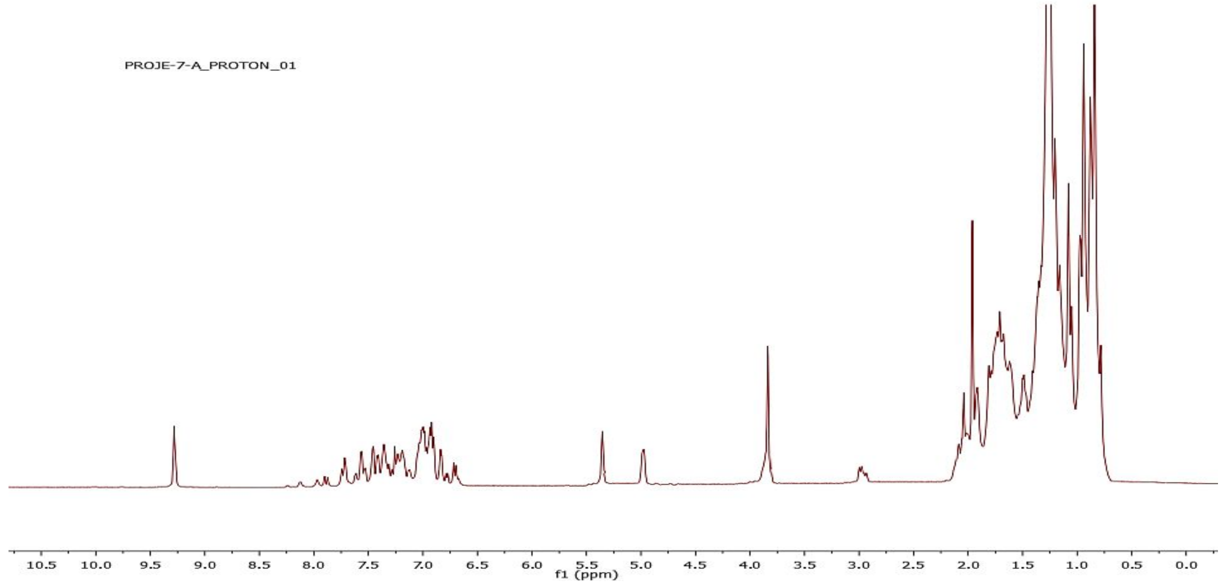

Figure S63. ^{1}H-NMR spectrum of compound **4g** (600 MHz, $CDCl_3$, ppm)

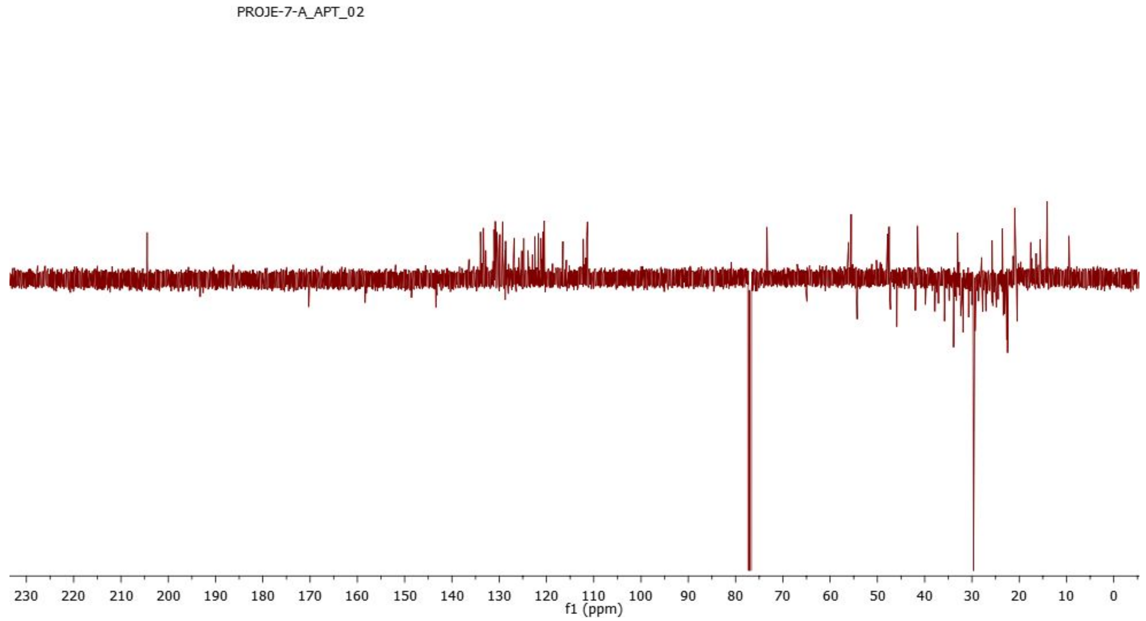

Figure S64. APT-NMR spectrum of compound **4g** (150 MHz, $CDCl_3$, ppm)

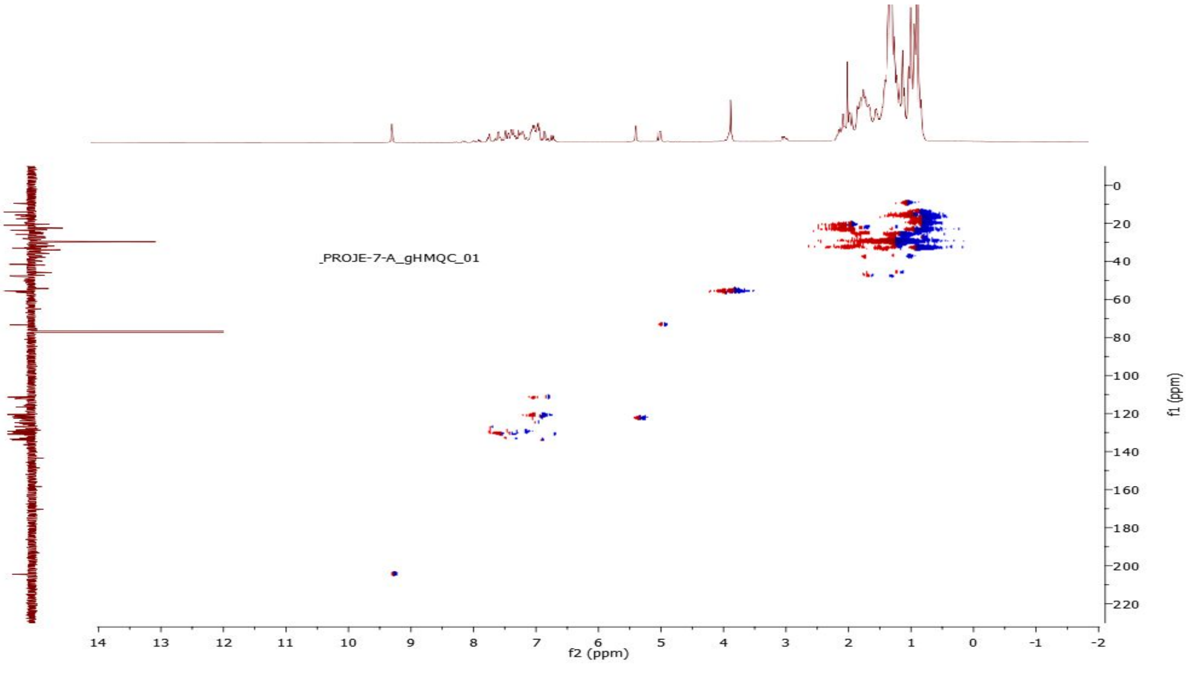

Figure S65. HMQC spectrum of compound **4g**

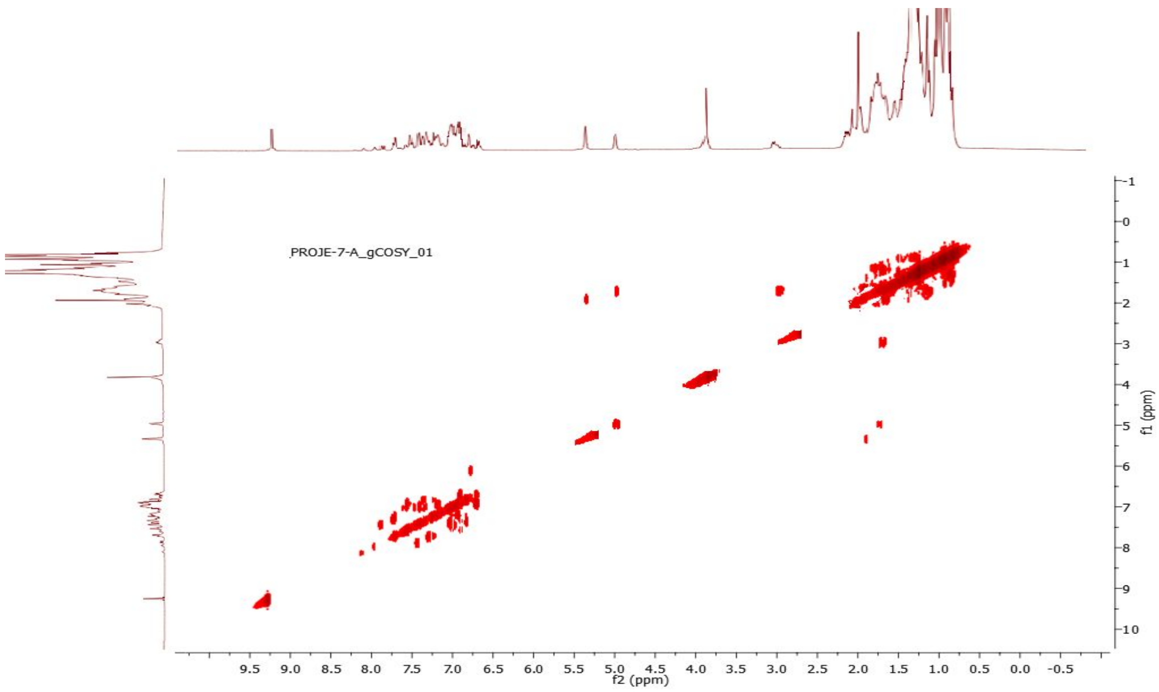

Figure S66. ^{1}H-^{1}H COSY-NMR spectrum of compound **4g** (400 MHz, CD_3OD, ppm)

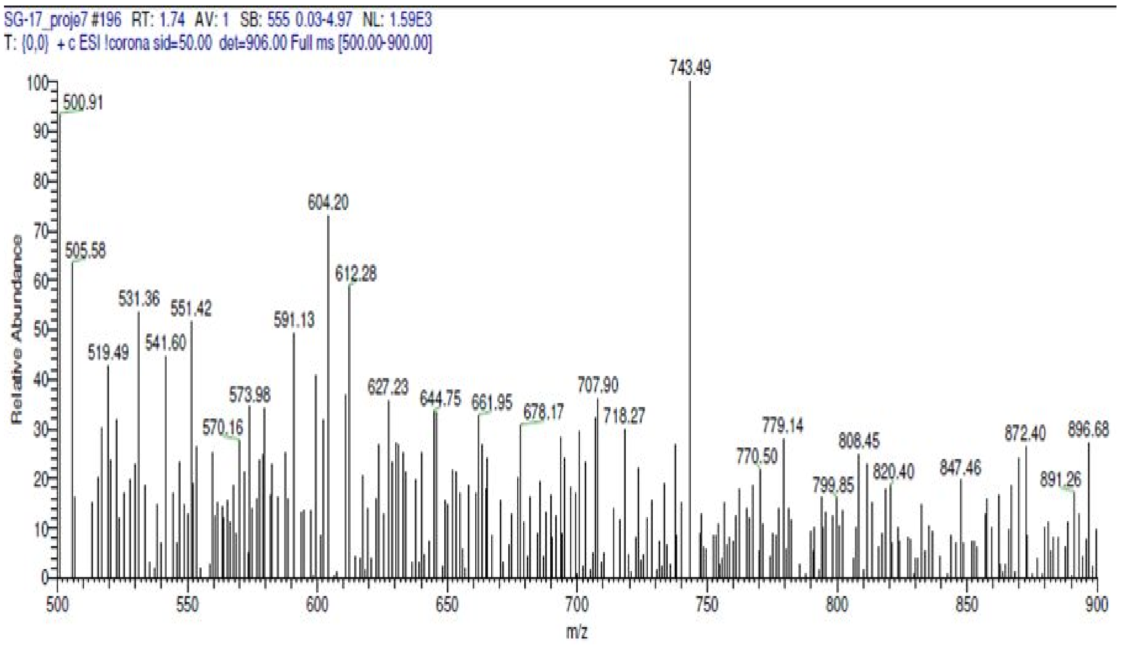

Figure S67. Mass spectrum of compound **4g** (Thermo Scientific/ Surveyor MSQ)

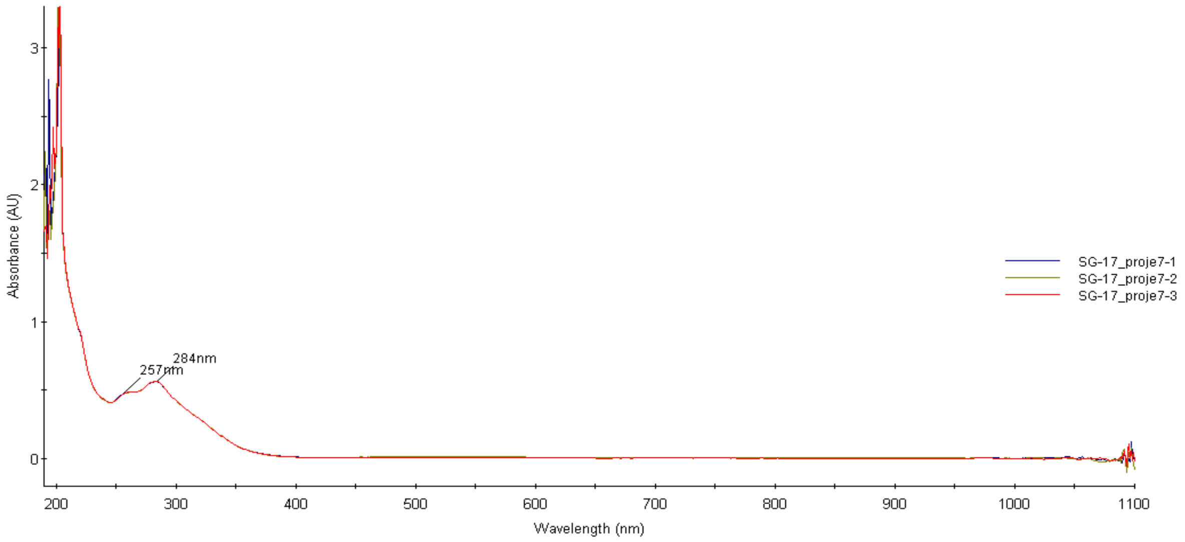

Figure S68. UV spectrum of compound **4g**

4h: Acetyl Gypso-COO-Chalcone Hybrid Compound

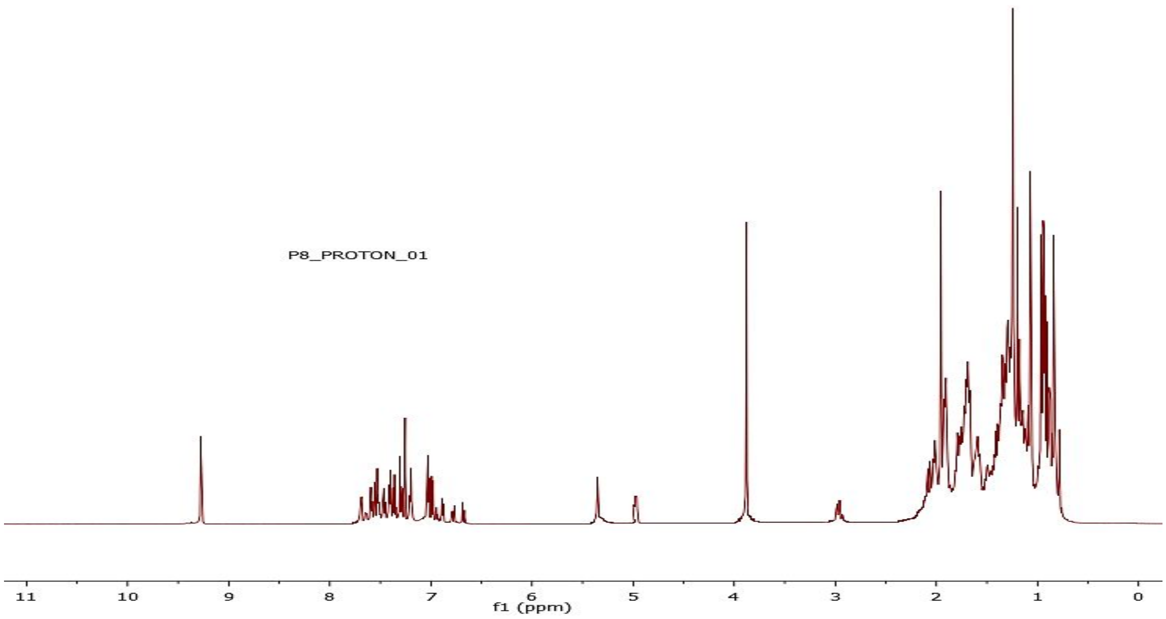

Figure S69. ^{1}H-NMR spectrum of compound **4h** (600 MHz, $CDCl_3$, ppm)

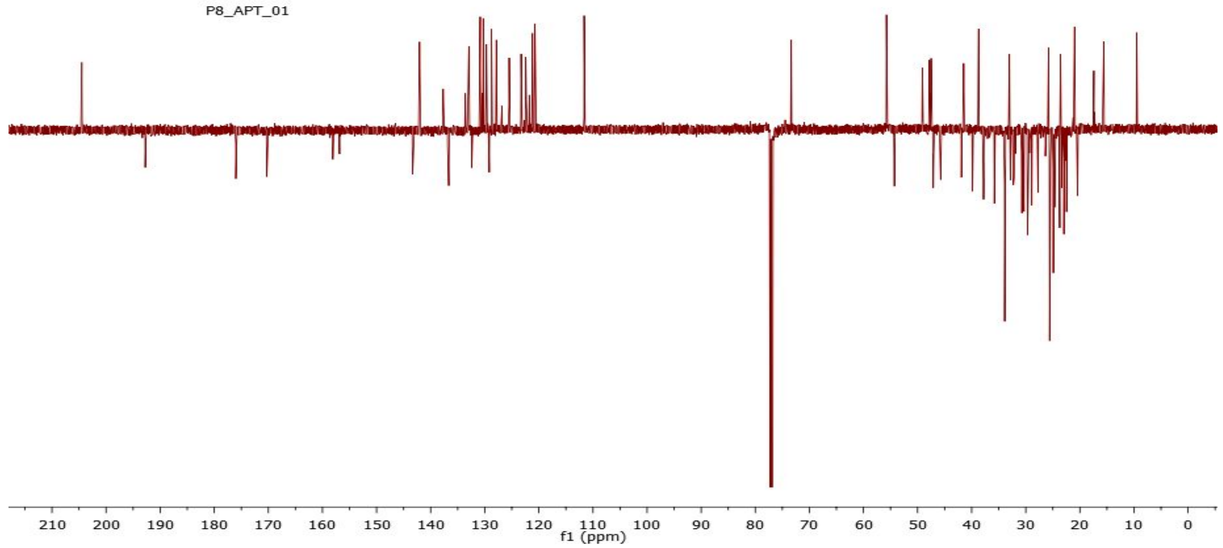

Figure S70. APT-NMR spectrum of compound **4h** (150 MHz, $CDCl_3$, ppm)

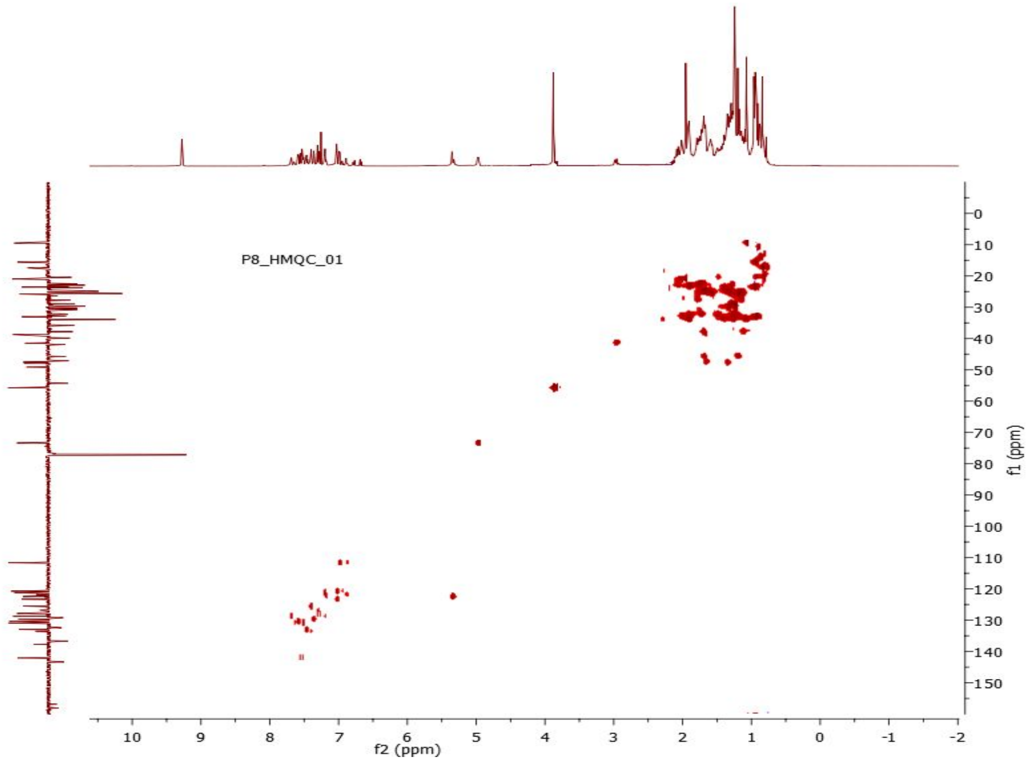

Figure S71. HMQC spectrum of compound **4h**

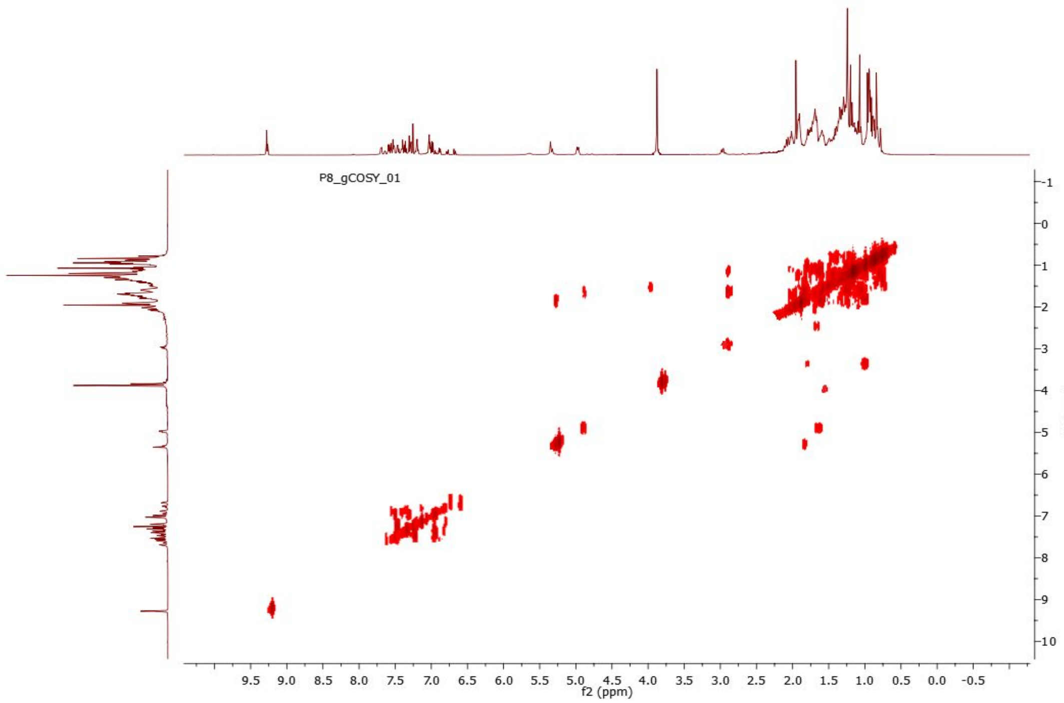

Figure S72. ^{1}H-^{1}H COSY-NMR spectrum of compound **4h (**400 MHz, CD_3OD, ppm)

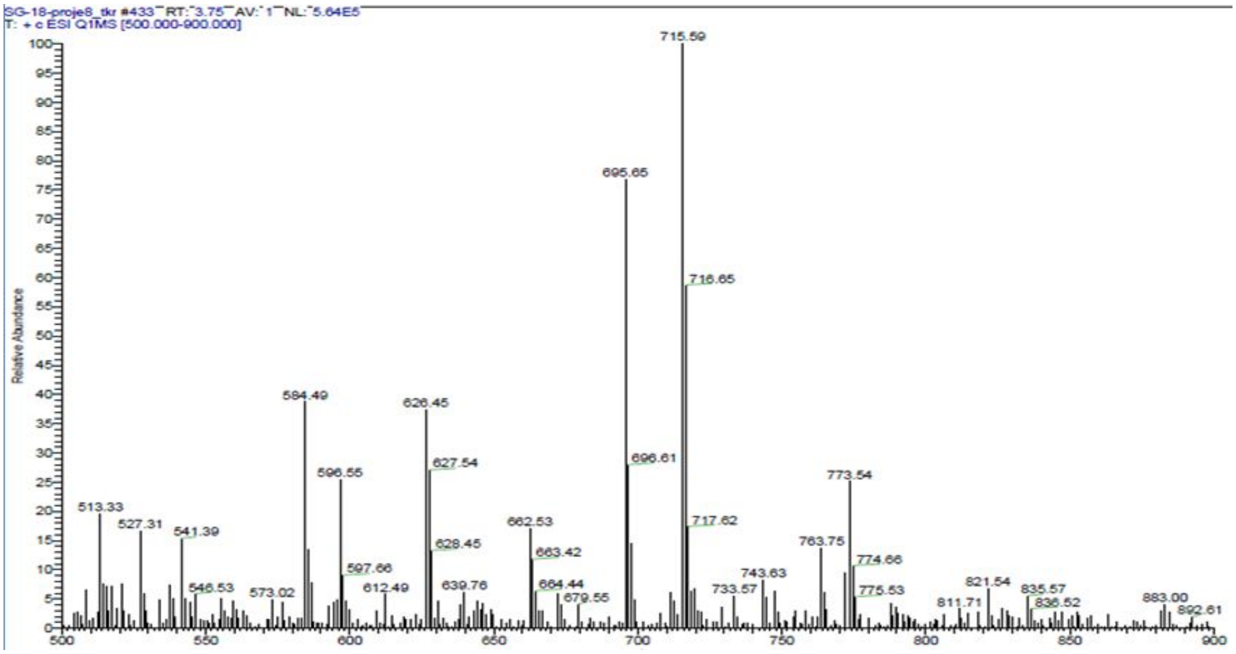

Figure S73. Mass spectrum of compound **4h** (Thermo Scientific/ Surveyor MSQ)

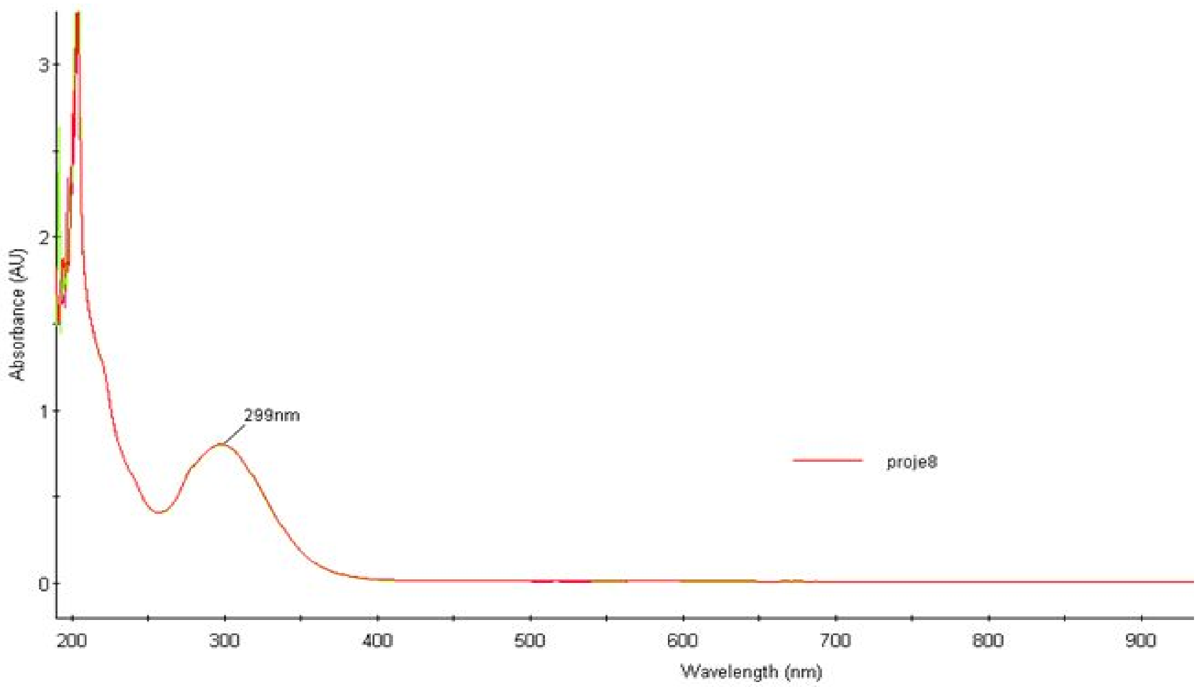

Figure S74. UV spectrum of compound **4h**

4i: Acetyl Gypso-COO-Chalcone Hybrid Compound

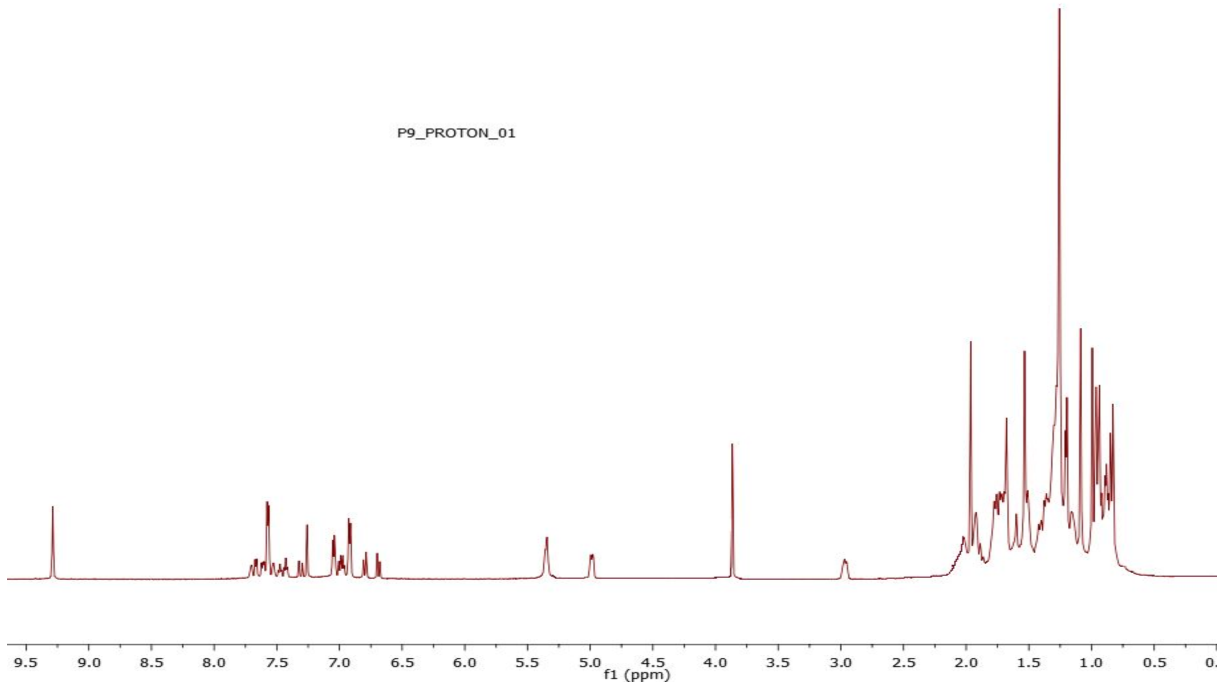

Figure S75. ^{1}H-NMR spectrum of compound **4i** (600 MHz, $CDCl_3$, ppm)

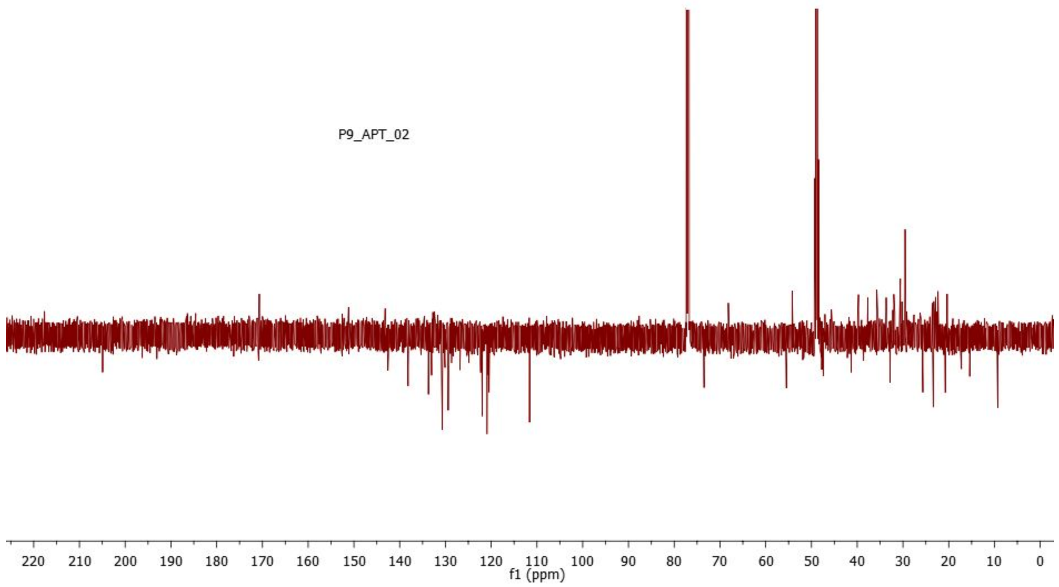

Figure S76. APT-NMR spectrum of compound **4i** (150 MHz, $CDCl_3$, ppm)

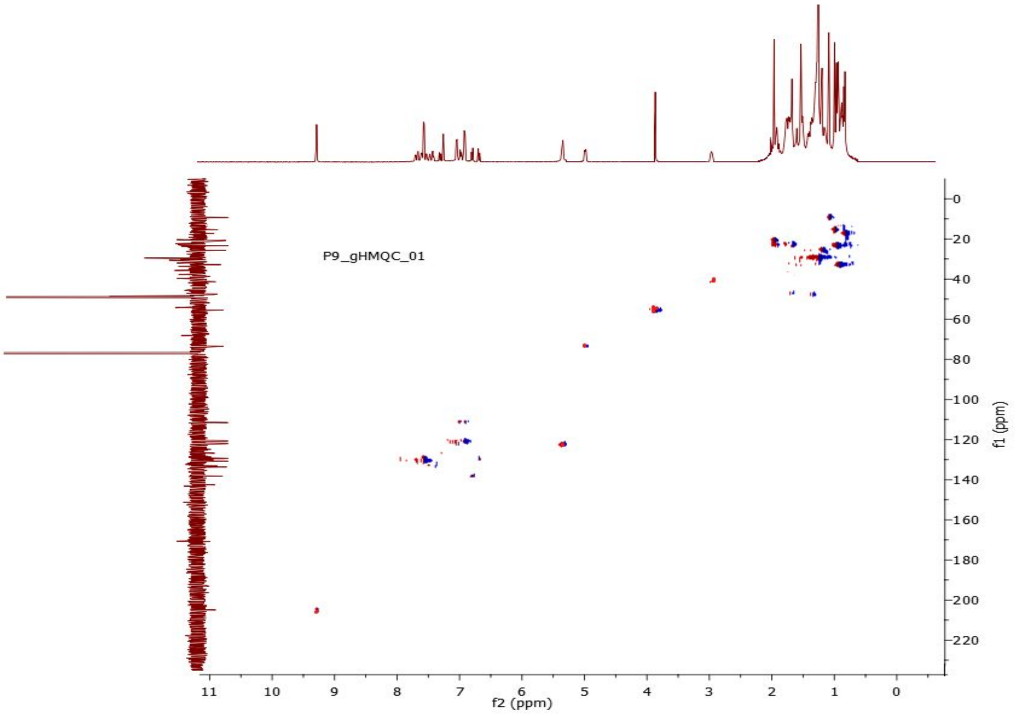

Figure S77. HMQC spectrum of compound **4i**

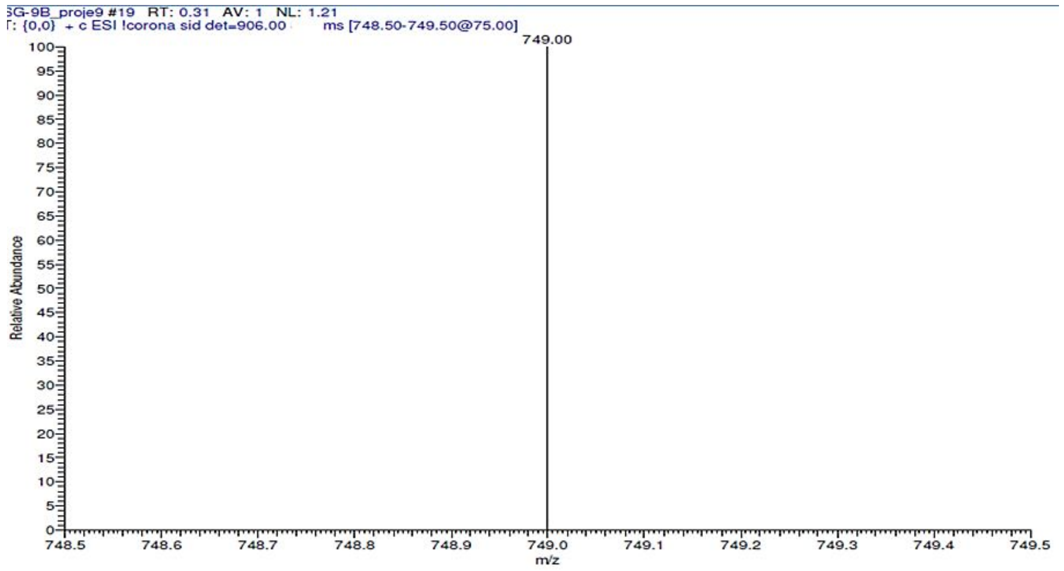

Figure S78. Mass spectrum of compound **4i** (Thermo Scientific/ Surveyor MSQ)

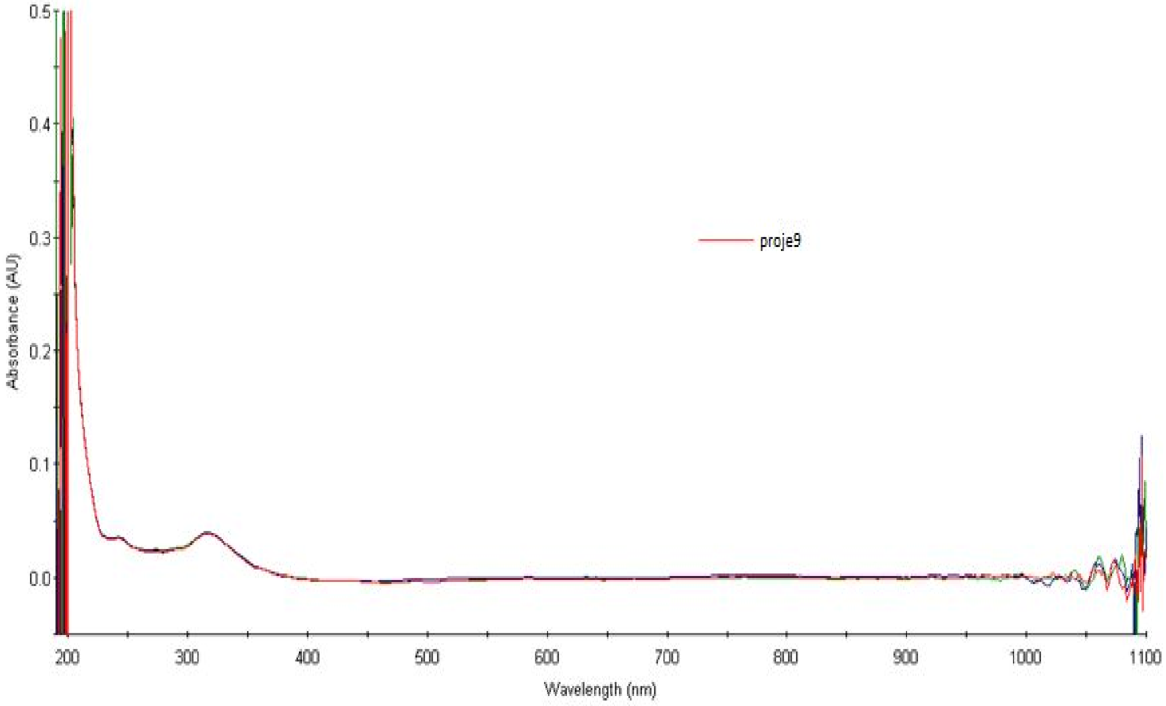

Figure S79. UV spectrum of compound **4i**
